# 2′-*O*-Trifluoromethylated RNA – a powerful modification for RNA chemistry and NMR spectroscopy†‡

**DOI:** 10.1039/d0sc04520a

**Published:** 2020-09-24

**Authors:** Maximilian Himmelstoß, Kevin Erharter, Eva Renard, Eric Ennifar, Christoph Kreutz, Ronald Micura

**Affiliations:** University of Innsbruck, Institute of Organic Chemistry, Center for Molecular Biosciences (CMBI) Innrain 80-82 6020 Innsbruck Austria ronald.micura@uibk.ac.at christoph.kreutz@uibk.ac.at; Université de Strasbourg, Architecture et Réactivité de l'ARN–CNRS UPR 9002, Institut de Biologie Moléculaire et Cellulaire 67000 Strasbourg France e.ennifar@ibmc-cnrs.unistra.fr

## Abstract

New RNA modifications are needed to advance our toolbox for targeted manipulation of RNA. In particular, the development of high-performance reporter groups facilitating spectroscopic analysis of RNA structure and dynamics, and of RNA–ligand interactions has attracted considerable interest. To this end, fluorine labeling in conjunction with ^19^F-NMR spectroscopy has emerged as a powerful strategy. Appropriate probes for RNA previously focused on single fluorine atoms attached to the 5-position of pyrimidine nucleobases or at the ribose 2′-position. To increase NMR sensitivity, trifluoromethyl labeling approaches have been developed, with the ribose 2′-SCF_3_ modification being the most prominent one. A major drawback of the 2′-SCF_3_ group, however, is its strong impact on RNA base pairing stability. Interestingly, RNA containing the structurally related 2′-OCF_3_ modification has not yet been reported. Therefore, we set out to overcome the synthetic challenges toward 2′-OCF_3_ labeled RNA and to investigate the impact of this modification. We present the syntheses of 2′-OCF_3_ adenosine and cytidine phosphoramidites and their incorporation into oligoribonucleotides by solid-phase synthesis. Importantly, it turns out that the 2′-OCF_3_ group has only a slight destabilizing effect when located in double helical regions which is consistent with the preferential C3′-endo conformation of the 2′-OCF_3_ ribose as reflected in the ^3^*J* (H1′–H2′) coupling constants. Furthermore, we demonstrate the exceptionally high sensitivity of the new label in ^19^F-NMR analysis of RNA structure equilibria and of RNA–small molecule interactions. The study is complemented by a crystal structure at 0.9 Å resolution of a 27 nt hairpin RNA containing a single 2′-OCF_3_ group that well integrates into the minor groove. The new label carries high potential to outcompete currently applied fluorine labels for nucleic acid NMR spectroscopy because of its significantly advanced performance.

## Abstract 2

The new 2′-OCF_3_ label for nucleic acid NMR spectroscopy carries high potential to outcompete currently applied fluorine labels because of significantly advanced performance.

## Graphical abstract

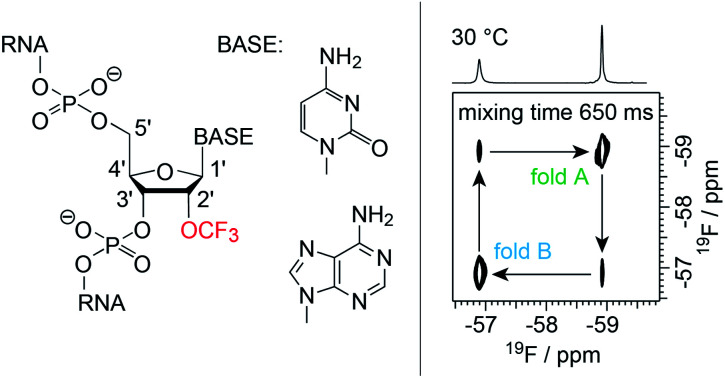

## Introduction

The attractiveness of fluorine labeling of biomolecules for ^19^F-NMR spectroscopic applications originates from its unique properties, namely a 100% natural abundance, high NMR sensitivity, and large chemical shift dispersion.^1–9^ Moreover, fluorine is bio-orthogonal, meaning that it is hardly encountered in native biomolecular systems.^10–15^ Appropriate probes for ribonucleic acids have mainly focused on single fluorine atoms attached to the 5-position of pyrimidine nucleobases^16–20^ or at the ribose 2′-position.^21–25^ To further increase sensitivity, trifluoromethyl labeling approaches have been sought after,^26,27^ one of them focuses on ribose 2′-trifluoromethylthio (2′-SCF_3_) modifications.^28–30^ A drawback of the 2′-SCF_3_ group, however, is its strong impact on RNA thermodynamic stability when located in base-paired regions.^29^ Interestingly, the structurally related 2′-*O*-trifluoromethyl (2′-OCF_3_) RNA has not been reported thus far. To the best of our knowledge, only one study is available that describes the thermodynamic stabilities of a short DNA containing a single 2′-OCF_3_ group that is paired to either a complementary DNA or RNA strand.^31^ We expected the 2′-OCF_3_ modification highly beneficial for RNA and ^19^F-NMR spectroscopic applications to analyze structural dynamics and ligand interactions, and therefore, we set out to overcome the underlying challenges in chemical synthesis. In this work, we present synthetic routes toward 2′-OCF_3_ nucleoside phosphoramidites and their incorporation into oligoribonucleotides by RNA solid-phase synthesis. Furthermore, we describe the impact of the 2′-OCF_3_ group on thermodynamic stability of RNA double helices which is only slightly destabilizing when located in double helical regions. This finding is consistent with a preferential C3′-endo conformation of the 2′-OCF_3_ ribose in short single stranded RNA as shown by measurements and interpretation of H1′–H2′ coupling constants. Moreover, we demonstrate the exceptionally high sensitivity of the new label in ^19^F-NMR analysis of RNA structure equilibria and of RNA–small molecule interactions. The study is complemented by crystal structures of an RNA hairpin including 2′-OCF_3_ modifications.

## Results and discussion

The syntheses of 2′-OCF_3_ nucleoside phosphoramidites followed the previously described route of a 2′-OCF_3_ adenosine derivative^31^ and involved a method by Hiyama *et al.* who reported that methyl xanthates R-OC(S)SMe are converted into trifluoromethyl ethers R-OCF_3_ by treatment with pyridinium poly(hydrogen fluoride) (HF/pyridine) in the presence of 1,3-di-bromo-5,5-dimethylhydantoin (DBH).^32^ A drawback however is the low yield of this transformation.

### Synthesis of 2′-OCF_3_ cytidine

**Scheme 1:**
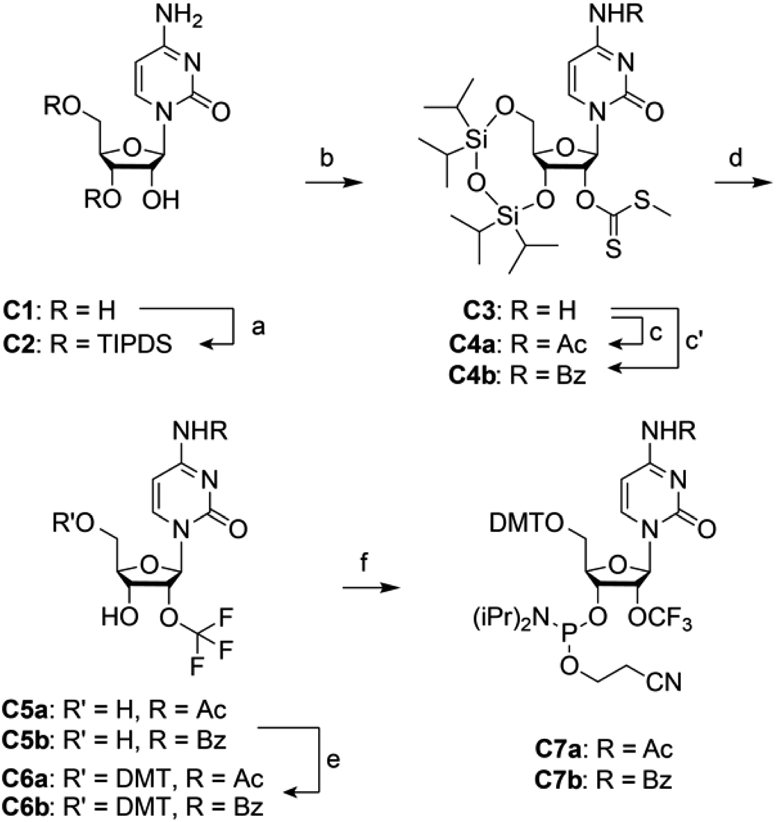
Synthesis of 2′-OCF_3_ cytidine building block **C7**. Reaction conditions: (a) 1.1 equiv. TIPDSCl_2_, in pyridine, room temperature, 5 h, 86%; (b) 1.2 equiv. ^*t*^BuLi, 9.0 equiv. CS_2_, 3.0 equiv. CH_3_I, in THF, −75 °C to room temperature, 18 h, 77%; (c) 3.0 equiv. acetic anhydride, in pyridine, room temperature, 16 h, 90%; (c′) 1.1 equiv. benzoic anhydride, in DMF, room temperature, 16 h, 90%; (d) 5.0 equiv. NBS, in HF pyridine and CH_2_Cl_2_, –75 °C to 0 °C, 3 h, 11% for **C5a**, 16% for **C5b**; (e) 1.4 equiv. DMTCl, 0.4 equiv. DMAP, in pyridine, room temperature, 16 h, 76% for **C6a**, 86% for **C6b**; (f) 2.5 equiv. 2-cyanoethyl *N*,*N*-diisopropylchlorophosphoramidite, 7.5 equiv. iPr_2_NEt, 0.5 equiv. 1-methylimidazole, in CH_2_Cl_2_, room temperature, 2 h, 74% for **C7a**, 92% for **C7b**.

For building block **C7** (Scheme 1), we started the synthesis from cytidine **C1**, which was simultaneously protected at the 3′ and 5′ oxygen atoms with the tetraisopropyldisiloxane (TIPDS) group (Scheme 1). Compound **C2** was then treated with *tert*-BuLi, carbon disulfide and methyl iodide in tetrahydrofuran at −78 °C to yield the 2′-*O*-[(methylthio)-thiocarbonyl]cytidine derivative **C3**. After acetylation of the exocyclic NH_2_ group to furnish **C4a**, the desired 2′-*O*-trifluoromethyl derivative **C5a** was obtained in low yields by treatment with pyridinium poly(hydrogen fluoride) (HF/pyridine) in the presence of *N*-bromosuccinimide (NBS) instead of DBH as mentioned above. Finally, **C5a** was transformed into the dimethoxytritylated compound **C6a**, and conversion into the corresponding phosphoramidite **C7a** was accomplished in good yields by reaction with 2-cyanoethyl *N*,*N*-diisopropylchlorophosphoramidite. Starting with cytidine **C1**, our route provides **C7a** in 4% overall yield in six steps with six chromatographic purifications; in total, 0.5 g of **C7a** was obtained in the course of this study. We mention that the yields of trifluoromethylation can be increased by switching from *N*^4^-acetyl to *N*^4^-benzoyl protection (Scheme 1). The higher stability of the latter (compound **4b**) against hydrolysis increased the yields by 50% comparing the transformation of **4a** into **5a** with **4b** into **5b**. However, one has to be aware that *N*^4^-benzoyl cytidine in synthetic RNA can be transaminated to some extent (<10%) when deprotection reagents containing methylamine are applied.^33,34^

### Synthesis of 2′-OCF_3_ adenosine

**Scheme 2:**
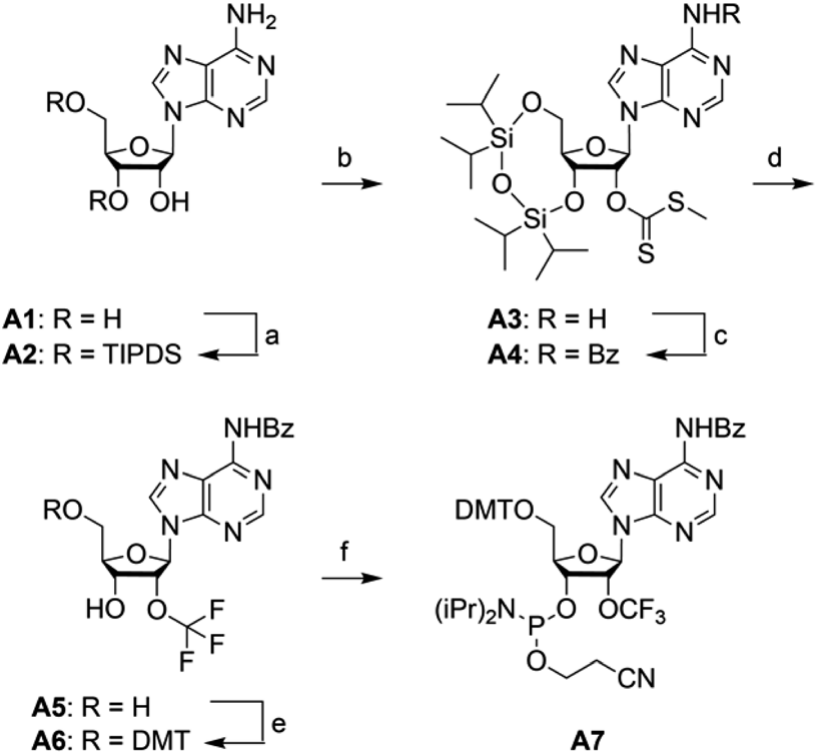
Synthesis of 2′-OCF_3_ adenosine building block **A7**. Reaction conditions: (a) 1.1 equiv. TIPDSCl_2_, in pyridine, room temperature, 5 h, 95%; (b) 1.2 equiv. ^*t*^BuLi, 9.0 equiv. CS_2_, 3.0 equiv. CH_3_I, in THF, −75 °C to room temperature, 18 h, 74%; (c) 2.5 equiv. benzoylchloride, 6 equiv. NH_3_, in pyridine, room temperature, 16 h, 89%; (d) 5.0 equiv. NBS, in HF pyridine and CH_2_Cl_2_, –75 °C to 0 °C, 3 h, 26%; (e) 1.5 equiv. DMTCl, 0.5 equiv. DMAP, in pyridine, room temperature, 16 h, 88%; (f) 2.5 equiv. 2-cyanoethyl *N*,*N*-diisopropylchlorophosphoramidite, 7.5 equiv. iPr_2_NEt, 0.5 equiv. 1-methylimidazole, in CH_2_Cl_2_, room temperature, 2 h, 82%.

For building block **A7** (Scheme 2), we started the synthesis from adenosine **A1**, which was simultaneously protected at the 3′ and 5′ oxygen atoms with the TIPDS group (Scheme 2). Compound **A2** was treated with *tert*-BuLi, carbon disulfide and methyl iodide in tetrahydrofuran at −78 °C to yield the 2′-*O*-[(methylthio)-thiocarbonyl]cytidine derivative **A3**. After benzoylation of the exocyclic NH_2_ group to furnish **A4**, the desired 2′-*O*-trifluoromethyl derivative **A5** was obtained by treatment with HF/pyridine in the presence of NBS. Yields were significantly higher compared to the same transformation on cytidine (**C4** into **C5**). Finally, **A5** was transformed into the dimethoxytritylated compound **A6**, and conversion into the corresponding phosphoramidite **A7** was accomplished in good yields by reaction with 2-cyanoethyl *N*,*N*-diisopropylchlorophosphoramidite. Starting with adenosine **A1**, our route provides **A7** in 12% overall yield in six steps with six chromatographic purifications; in total, 1.2 g of **A7** was obtained in the course of this study.

We mention that in an attempt to increase the yields for the 2′-*O* trifluoromethylation step, we tested silver-mediated oxidative *O*-trifluoromethylation of a 3′,5′-*O* protected adenosine derivative using TMSCF_3_, following a published protocol,^65^ but unfortunately failed. Currently, we are planning to elaborate routes for 2′-OCF_3_ uridine and guanosine building blocks *via* the here described methyl xanthate intermediates. This should be feasible provided proper protection concepts for N3-H and N1-H, respectively, can be identified.

### RNA solid-phase synthesis

**Fig. 1:**
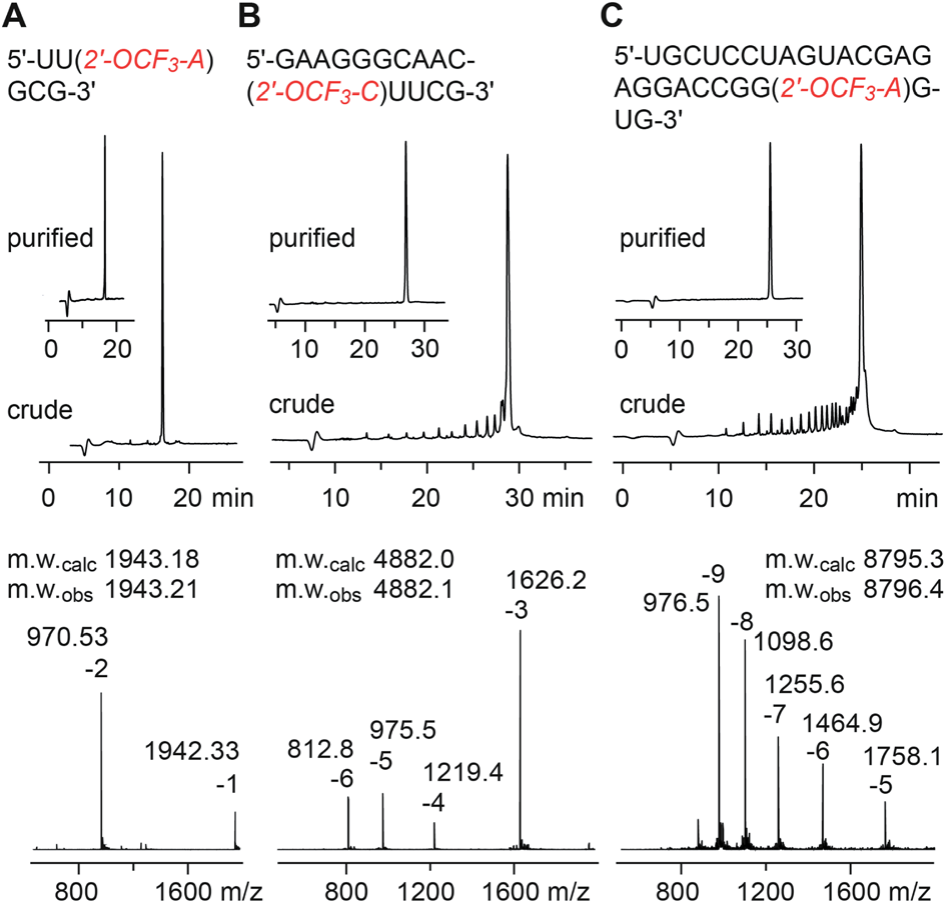
Characterization of 2′-OCF_3_ modified RNA. Anion-exchange HPLC traces (top) of 6 nt RNA (A), 15 nt RNA (B), and 27 nt RNA (C), and corresponding LC-ESI mass spectra (bottom). HPLC conditions: Dionex DNAPac column (4 × 250 mm), 80 °C, 1 mL min^−1^, 0–60% buffer B in 45 min; buffer A: Tris–HCl (25 mM), urea (6 M), pH 8.0; buffer B: Tris–HCl (25 mM), urea (6 M), NaClO_4_ (0.5 M), pH 8.0. For LC-ESI MS conditions, see the ESI.‡

The solid-phase synthesis of RNA with site-specific 2′-OCF_3_ modifications was performed following the 2′-*O*-[(triisopropylsilyl)oxy]methyl (TOM) approach.^35,36^ Coupling yields of the novel building blocks were higher than 98% according to the trityl assay. Cleavage of the oligonucleotides from the solid support and their deprotection were performed using methylamine in water/ethanol or methylamine/ammonia in water (AMA), followed by treatment with tetra-*n*-butylammonium fluoride (TBAF) in tetrahydrofuran. Salts were removed by size-exclusion chromatography, and RNAs were purified by anion-exchange chromatography under denaturating conditions (6 M urea, 80 °C; Fig. 1 and ESI Table 1‡). The molecular weights of the purified RNAs were confirmed by liquid-chromatography (LC) electrospray-ionization (ESI) mass spectrometry (MS). The sequences of 2′-OCF_3_ containing RNAs synthesized in the course of this study are listed in ESI Table 1.‡

### Thermodynamic stability of 2′-OCF_3_ modified RNA

**Fig. 2:**
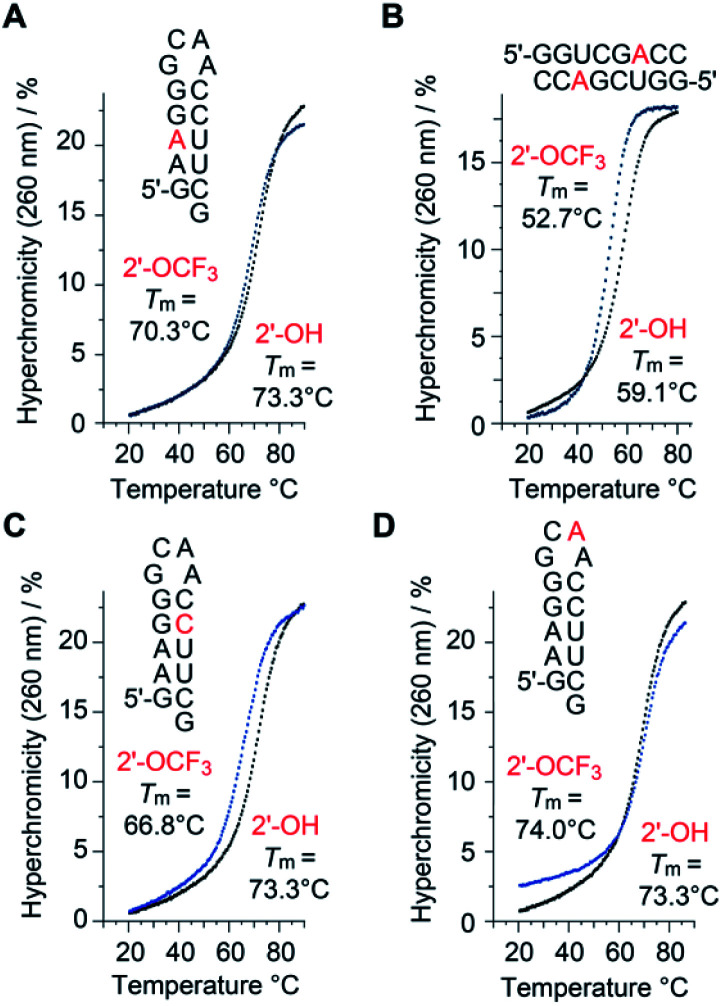
Thermal stabilities of unmodified *versus* 2′-OCF_3_ modified oligoribonucleotides. UV-melting profiles of hairpin and self-complementary duplex RNAs with the modification (either at adenosine or cytidine) located in the base-pairing region (A–C), and UV-melting profile of the same hairpin sequence with the modification in the GNRA loop motif (D). Conditions: c(RNA) = 12 μM; 10 mM Na_2_HPO_4_, 150 mM NaCl, pH 7.0. Nucleotides in red color indicate the positions for 2′-OCF_3_ modification.

A single 2′-OCF_3_ adenosine exhibited only moderate attenuation of RNA double helix stability if the modification was located in the Watson–Crick base-pairing region (Table 1). For instance, UV melting profile analysis^37,38^ of the hairpin-forming RNA 5′-GA(2′-OCF_3_-A)GG-GCAA-CCUUCG (Fig. 2A) revealed a decrease of the *T*_m_ value by 3 °C determined at micromolar RNA concentrations (*T*_m_ 70.3 °C), compared to the unmodified counterpart (*T*_m_ 73.3 °C). We remind that the same RNA but with a 2′-SCF_3_ modification at the same nucleotide position caused a much stronger drop in the *T*_m_ value, namely by 14 °C (Table 1).^29^ As a second example, the palindromic RNA 5′-GGUCG(2′-OCF_3_-A)CC (Fig. 2B) also suffered from an average *T*_m_ value decrease of 3 °C per 2′-OCF_3_-A modification (micromolar RNA concentrations), compared to the unmodified counterpart.

**Table 1:** Thermodynamic parameters of 2′-OCF_3_ modified RNA obtained by UV melting profile analysis (including unmodified and 2′-SCF_3_ modified reference RNAs)^a^

Sequence (5′ → 3′)	*T*_m_ [°C]	Δ*G*°_298_ [kcal mol^−1^]	Δ*H*° [kcal mol^−1^]	Δ*S*° [cal mol^−1^ K^−1^]
GGUCGACC	59.1	−14.3	−72.0	−193
GGUCG**A̲**CC	52.7	−12.6	−69.1	−189
GAAGG-GCAA-CCUUCG	73.3	−6.0	−46.0	−134
GA**A̲**GG-GCAA-CCUUCG	70.3	−5.8	−46.7	−137
GA 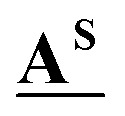 GG-GCAA-CCUUCG^30^	57.4	−5.5	−57.9	−176
GAAGG-GCAA-C**C̲**UUCG	66.8	−4.9	−41.7	−124
GAAGG-GCAA-C 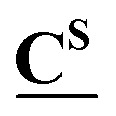 UUCG^29^	53.3	−4.4	−50.4	−154
GAAGG-GC**A̲**A-CCUUCG	74.0	−8.3	−60.9	−177

a A̲ 2′-OCF_3_ adenosine, C̲ 2′-OCF_3_ cytidine, 
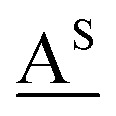
 2′-SCF_3_ adenosine,^30^
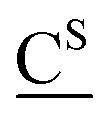
 2′-SCF_3_ cytidine.^29^ Buffer: 10 mM Na_2_HPO_4_, 150 mM NaCl, pH 7.0. Δ*H* and Δ*S* values were obtained by van't Hoff analysis according to ref. 37 and 38. Errors for Δ*H* and Δ*S*, arising from non-infinite cooperativity of two-state transitions and from the assumption of a temperature-independent enthalpy, are typically 10–15%. Additional error is introduced when free energies are extrapolated far from melting transitions; errors for Δ*G* are typically 3–5%.

Next, the same hairpin was analyzed with 2′-OCF_3_ at cytidine instead of adenosine, 5′-GAAGG-GCAA-C(2′-OCF_3_-C)UUCG (Fig. 2C). The destabilization was reflected by a 6.5 °C lower *T*_m_ value compared to the unmodified hairpin, still significantly less compared to the 2′-SCF_3_-C modification that caused a *T*_m_ reduction by 20 °C of this hairpin (Table 1).^29^ We point out that the significantly larger destabilization of 2′-SCF_3_ compared to the 2′-OCF_3_ modified double helices is of entropic origin (Table 1).

Notably, when the 2′-OCF_3_ group resides in a single-stranded region, the impact on thermodynamic stability is minor, and in the case of the extra-stable GNRA loop motif even modestly stabilizing, reflected by an increase of the *T*_m_ value and a favorable Δ*G* value for 5′-GAAGG-GC(2′-OCF_3_-A)A-CCUUCG (Fig. 2D and Table 1). The stabilization is rationalized by the fact that unmodified adenosine in GCA̲A loops preferentially adopts the C3′-endo ribose pucker (70%).^39–41^ The C3′-endo conformation in this loop becomes locked when the 2′-OCF_3_ group is attached, as verified by NMR spectroscopic analysis (ESI Fig. 1A‡). The very same hairpin was also used to demonstrate the impressive ^19^F NMR spectroscopic sensitivity of the 2′-OCF_3_ label compared to a 2′-F label (ESI Fig. 1B and C‡).

Taken together, our original expectation that the 2′-OCF_3_ modification compared to 2′-SCF_3_ is more potent for a broad scope of applications because of being less destabilizing and retaining the high sensitivity, turned out to be correct (Fig. 2, Table 1 and ESI Fig. 1‡).

### 2′-OCF_3_ ribose conformation

**Fig. 3:**
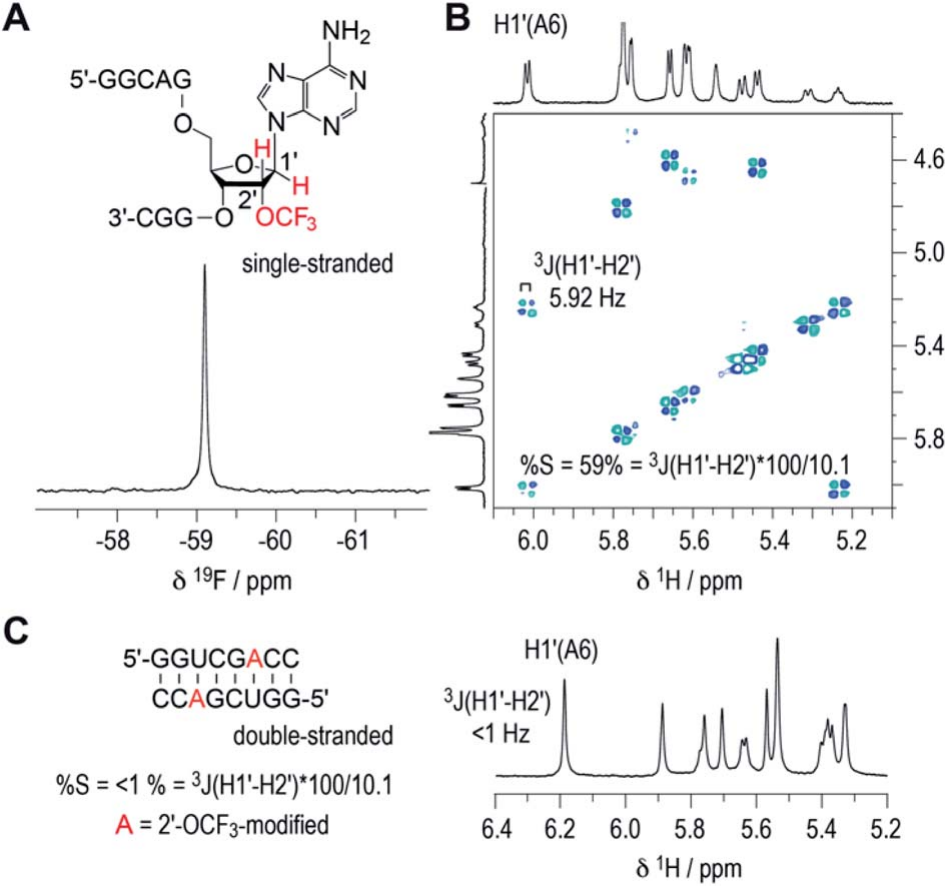
^19^F (A) and ^1^H/^1^H DQFCOSY (B) NMR spectra of single-stranded RNA 5′-GGCAG(2′-OCF_3_-A)GGC. For the 2′-OCF_3_ adenosine moiety, the 3-bond scalar coupling constant of H1′ and H2′ (^3^*J*H1′-H2′) was extracted from the corresponding crosspeak and amounted to 5.9 Hz. Assuming a pure C2′/C3′-endo equilibrium, this value is correlated to a C2′-endo (South) population of 58%.^38,40^ (C) ^1^H NMR spectrum of the self-complementary 8 nt RNA with 2′-OCF_3_-A group (red); ^3^*J*H1′–H2′ is smaller than 1 Hz, consistent with prevalent C3′-endo conformation in the double helix. Conditions: c(RNA) = 0.3 mM; 15 mM Na[AsO_2_(CH_3_)_2_]·3H_2_O, 25 mM NaCl, 3 mM NaN_3_, in D_2_O, pH 6.5, 296 K.

The inherent preference of a modified nucleoside to adopt either C2′-endo or C3′-endo conformation is crucial for its impact on thermodynamic base pairing stability.^42–45^ To analyze the ribose conformation of a 2′-OCF_3_ nucleoside in detail, we synthesized a short, single-stranded RNA, 5′-GGCAG(2′-OCF_3_-A)GGC, and determined the ^3^*J* (H1′–H2′) coupling constant from a 2D ^1^H,^1^H double quantum filtered COSY spectrum (DQFCOSY). The coupling constant amounted to 5.9 Hz, which translates into a population of about 60% of C2′-endo ribose conformation in the single strand (Fig. 3A and B; for ^1^H–^13^C HSQC spectrum see ESI Fig. 2‡). We then recorded the ^1^H NMR spectrum of a self-complementary, fully base-paired 8 nt RNA duplex with 2′-OCF_3_-A modification (Fig. 3C). The ^3^*J*H1′–H2′ was clearly smaller than 1 Hz, consistent with the requirement to adopt C3′-endo conformation in an RNA double helix to avoid steric clash. Notably, for the previously investigated 2′-SCF_3_ adenosine and -cytidine, the ^3^*J* (H1′–H2′) coupling constants amount to 9.7 and 10.4 Hz,^29,30^ respectively, provided they reside in a single-stranded short RNA. This in turn stands for a population of almost 100% of C2′-endo conformation. The strong C2′-endo preference of 2′-SCF_3_ modified nucleosides likely provides a rationale for the significantly higher thermodynamic destabilization if they are encountered in base paired regions.^29,30^ Further support for this hypothesis stems from the analysis of 5′-GGCAG(2′-F-A)GGC. For the 2′-F labeled nucleoside, the ^3^*J* (H1′–H2′) coupling constant was less than 1 Hz (ESI Fig. 3A‡) which corresponds to a population of almost 100% C3′-endo conformation in the single strand. This ‘pre-folding’ is a rationale for the slight stabilization when 2′-F becomes located in a double helix.^25^

### X-ray structures of 2′-OCF_3_ containing RNA

**Fig. 4:**
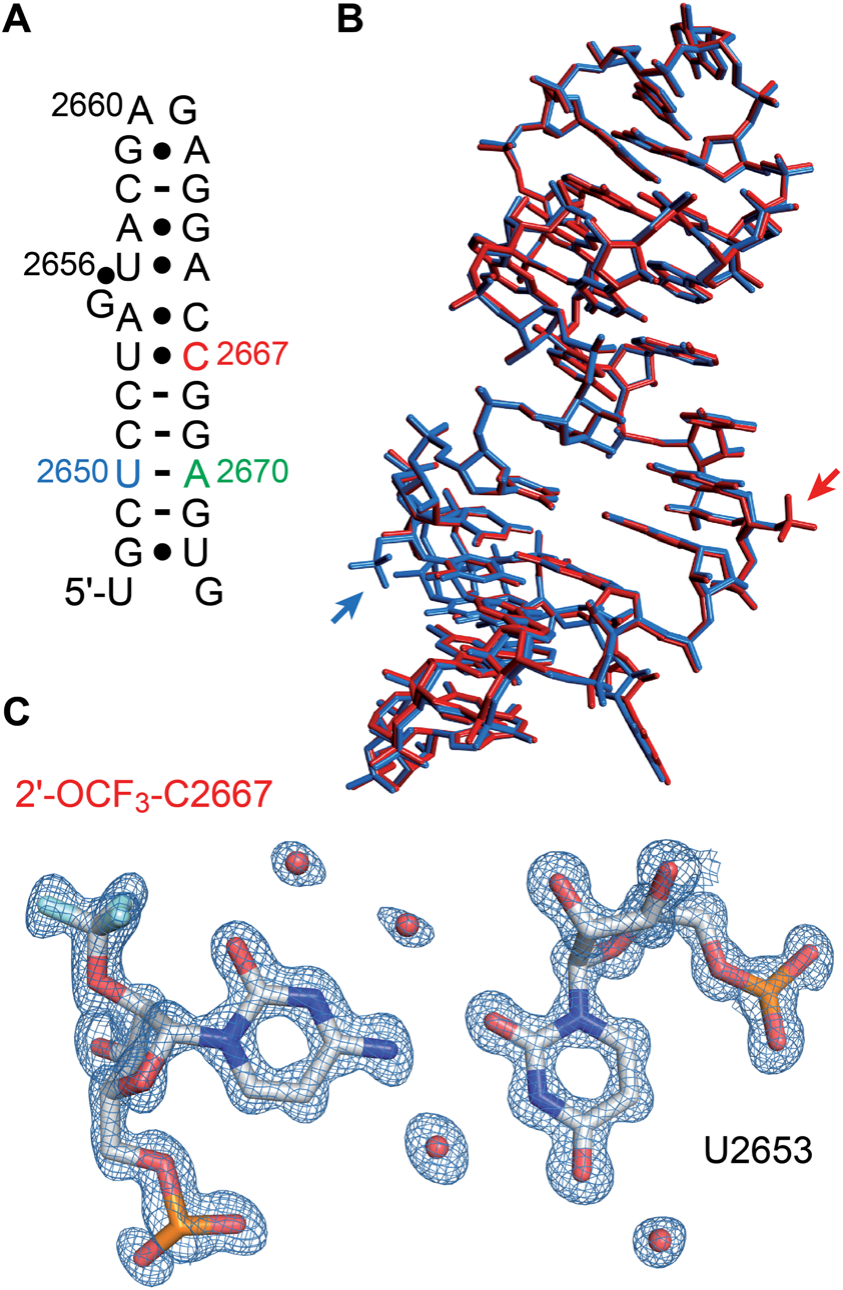
X-ray structure of 2′-OCF_3_ modified RNA at atomic resolution. (A) Secondary structure of the *E. coli* Sarcin-ricin stem-loop (SRL) RNA used for crystallization. The 2′-OCF_3_ modified cytidine is labeled in red. Adenosine in green highlights the second 2′-OCF_3_ modified RNA solved in this study (see ESI Fig. 4 and 5‡) and uridine in blue refers to a previously solved 2′-SCF_3_ modified SRL RNA for reason of comparison.^29^ (B) Superimposition of the 2′-OCF_3_–C2667 modified RNA (PDB ID 6ZYB red) and 2′-SCF_3_–U2650 modified RNA (PDB ID 4NHX, blue). Trifluoromethyl modifications are visible outside the RNA stem loop (on the center-right for OCF_3_ and in the bottom-left for SCF_3_). (C) 2*F*_obs_ − *F*_calc_ electron density map contoured at 1.5 *σ* level showing the U2653/2′-OCF_3_-C2667 base pair. Water molecules are shown as red spheres (PDB ID 6ZYB).

We set out for the X-ray analysis of a 2′-OCF_3_ modified RNA (Fig. 4). To this end, we used the 27 nt fragment of *E. coli* 23 S rRNA sarcin-ricin loop (SRL) region. This sequence is known to be a robust and well-behaved crystallization scaffold that can accommodate small modifications.^46,47^ For the installation of 2′-OCF_3_, we deemed nucleotide A2670 appropriate which forms a Watson−Crick base pair with U2650 in the regular A-form double helical region of this RNA, as well as nucleotide C2667 which forms a water-mediated mismatch with U2653. The latter RNA crystallized in the same tetragonal crystal form as the unmodified RNA^46^ and also diffracted to atomic resolution (0.9 Å). A new monoclinic crystal form was obtained with 2′-OCF_3_ A2670-modified SRL RNA (relative to former SRL RNA crystal forms), containing three molecules per asymmetric unit and diffracting to 2.4 Å resolution (ESI Table 2‡). The 2′-OCF_3_ modification did not affect the overall structure (r.m.s.d. ∼0.4 Å for 2′-OCF_3_–C2667 compared to the unmodified structure; PDB ID 3DVZ), including sugar puckers of modified positions (ESI Fig. 4‡). As previously observed for the 2′-SCF_3_–C2667 modification,^29^ a fluorine atom of the 2′-OCF_3_ group closely approached the oxygen atom of its cytosine nucleobase (O2). It is tempting to assume that the short distance observed (2.9 Å) is indicative of a halogen bond, however, fluorine (as opposed to chlorine, bromine, or iodine) usually retains a strongly electronegative electrostatic potential in biomolecules.^63,64^ Nevertheless, we note that the trifluoromethyl modification additionally comes also close the O4′ of the next G2668 residue (O–F distance is 3.2 Å, ESI Fig. 5‡) which further fuels speculations on possible stabilizing interactions.

### 2′-OCF_3_ NMR analysis of structure-ambivalent RNA

The biological function of RNA is determined by the secondary and tertiary structure, defining the RNA fold.^48^ The folding path usually proceeds *via* intermediates that represent local minima in the RNA folding free energy landscape. When these intermediates are separated by large energy barriers they constitute folding traps and, therefore, the timescale of the folding process may take up to minutes and longer. One of the most prominent examples is a single RNA sequence of 150 nucleotides in length that co-exists in two stable folds harboring distinct ribozyme activities.^49^ Even for shorter RNA, structure ambivalence is encountered, *e.g.* hairpin-duplex equilibria of palindromic RNAs or the occurrence of competing secondary structures of the same RNA.^50,51^ The latter plays a crucial role in bacterial gene regulation by so-called riboswitches which are located in the untranslated leader regions of nascent mRNA where terminator stem *versus* antiterminator formation during transcription signals ON or OFF.^52^

**Fig. 5:**
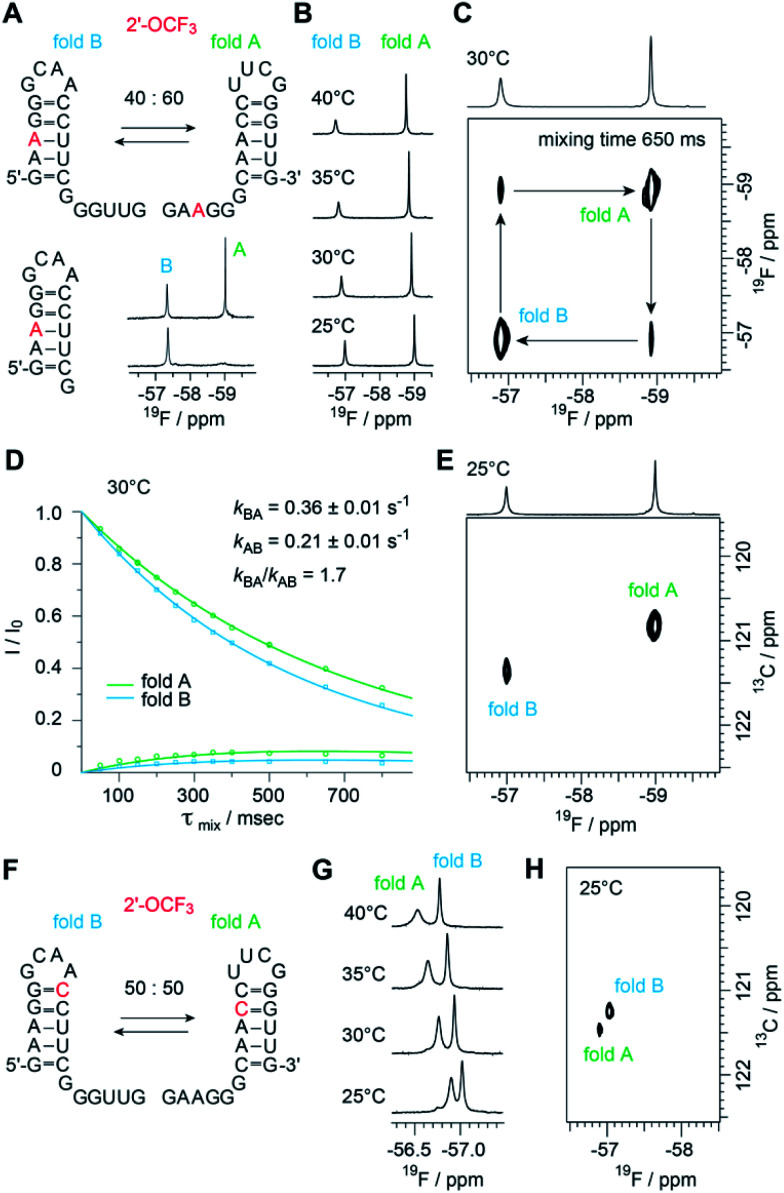
Structure probing of a bistable RNA with a single 2′-OCF_3_ label. (A) Secondary structure of full-length (top left) and reference RNA (bottom left), both with the 2′-OCF_3_ labeled A3, and ^19^F-NMR (470 MHz) spectra (bottom right). Conditions: c(RNA) = 0.3 mM, 15 mM sodium cacodylate, 25 mM NaCl, pH 6.5, H_2_O/D_2_O = 9 : 1, 298 K; (B) temperature-dependent series of ^19^F-NMR (564 MHz) spectra. (C) ^19^F/^19^F-EXSY (564 MHz) spectrum (650 ms mixing time, same conditions as in (A)). The crosspeaks between the ^19^F resonances assigned to the two folds originate from a chemical exchange process between the two folds at the millisecond time scale. (D) Exchange peaks build-up and *R*_1_ decay curves of the exchange experiment for residue A3 for the forward and backward folding event. Exchange rates are indicated, the following longitudinal relaxation rates were obtained: *R*_1A_ = 1.01 ± 0.11 s^−1^, *R*_1B_ = 1.56 ± 0.18 s^−1^. The experiments were run at mixing times ranging from 50 ms to 800 ms at 303 K. Dots are the experimental data, fits are shown as solid lines. Error rates were determined by Monte Carlo analysis with peak intensities randomly modulated according to noise levels exchange spectra. (E) ^19^F–^13^C HSQC spectrum of the RNA shown in panel A. (F) Same RNA but with 2′-OCF_3_ label at C10. (G) Temperature-dependent series of ^19^F-NMR spectra. (H) ^19^F–^13^C HSQC spectrum of the RNA shown in panel F.

Fig. 5 illustrates a short bistable RNA that competes between two defined secondary structures. The CF_3_ label is attached at the adenosine in sequence position 3 and hence resides in the double helical stem of one fold while it is found in the single stranded overhang of the alternative fold (Fig. 5A and ESI Fig. 6‡). At room temperature (and also at slightly increased temperatures), the two folds are in slow exchange with respect to the NMR time scale. Two distinct ^19^F resonances are obtained (Fig. 5A and B) and assigned by comparison to a short reference hairpin that matches one substructure and adopts a single fold only (Fig. 5A). Interestingly, the line-widths of the two ^19^F resonances arising from the 2′-OCF_3_ group in the two folding states exhibit a large difference. The half-height line-width is 7 Hz for the ‘single strand’–^19^F resonance while it is increased by a factor of almost three for the ‘double helix’–^19^F resonance (20 Hz). Likely, the rotation of the 2′-OCF_3_ group in the base-paired stem is sterically hindered leading to signal broadening due to exchange on the μs to ms time scale.

The sensitive 2′-OCF_3_ label was also used to analyze the underlying kinetics of refolding for the bistable RNA. For that purpose, we used ^19^F longitudinal exchange NMR spectroscopy.^53–55^ As expected we found two ^19^F correlation peaks (fold A and fold B) in the ^19^F NOESY exchange spectrum of the RNA (with a single 2′-OCF_3_ labeled adenosine) for which exchange peaks could be identified (Fig. 5C). The forward and backward folding rates of the secondary structure equilibrium were determined at 30 °C (Fig. 5D). We found a forward rate constant *k*_BA_ of 0.36 ± 0.01 s^−1^ and a rate constant *k*_AB_ for the folding process from state A to state B of 0.21 ± 0.01 s^−1^, giving a good agreement with the equilibrium constant obtained from peak integration (*K*^Int,30°C^_AB_ = 1.9 and *K*^30°C^_AB_ = *k*_BA_/*k*_AB_ = 1.7). Using our ^19^F labeling approach, we were thus able to characterize the refolding kinetics of the bistable RNA under near physiological conditions.

Furthermore, to analyze the impact of the labeling position we synthesized the same bistable RNA but with 2′-OCF_3_ at C10. As expected, we observed two distinct ^19^F NMR resonances for the two folds in slow exchange, albeit the chemical shift difference was smaller, consistent with the label located in double helical regions in both folds (Fig. 5F). The equilibrium position (50 : 50) (Fig. 5G and ESI Fig. 7‡) was only slightly shifted compared the 2′-OCF_3_ A3 labeled counterpart (40 : 60) and confirmed the flexibility for 2′-OCF_3_ positioning despite of being a non-isosteric label. At this point, we also mention that the native (unmodified) RNA exists in a 50 : 50 equilibrium as was determined earlier.^23^

With respect to increasing temperatures, the ^19^F NMR resonances shifted to lower magnetic field. This shift was more pronounced for the 2′-OCF_3_–C labeled RNA (Fig. 5G) compared to the 2′-OCF_3_–A labeled RNA (Fig. 5B), which might be due to fraying of the loop closing base pair of which 2′-OCF_3_–C is part of (Fig. 5F, fold B).

Finally, we characterized both RNAs by recording ^19^F–^13^C HMQC spectra (Fig. 5E and H). We were able to obtain high quality natural abundance ^19^F ^13^C correlation spectra for rather dilute samples (*ca.* 150 μM each fold) overnight. By adding the carbon dimension, better spectral dispersion is obtained, which in principle would allow to apply ^13^C ZZ exchange spectroscopy in 2′-O^13^CF_3_ labeled systems. It will be further highly interesting to explore the TROSY properties of the 2′-O^13^CF_3_ methyl group in RNA by combining ^19^F and ^13^C stable isotope labeling. In a recent work by Sykes and co-workers no favorable TROSY effect in proteins was found using 3-bromo-1,1,1-trifluoroacetone as labeling reagents. The absence of the TROSY effect was attributed to the dominating CSA relaxation mechanism in the alpha CF_3_ group suggesting that gains from CF_3_-HMQC experiments should be only observable at low magnetic fields.^62^ Nevertheless, it remains to be clarified if the 2′-O^13^CF_3_ shows a more beneficial TROSY effect due to the different chemical environment.

### 2′-OCF_3_ NMR analysis of RNA-ligand binding

**Fig. 6:**
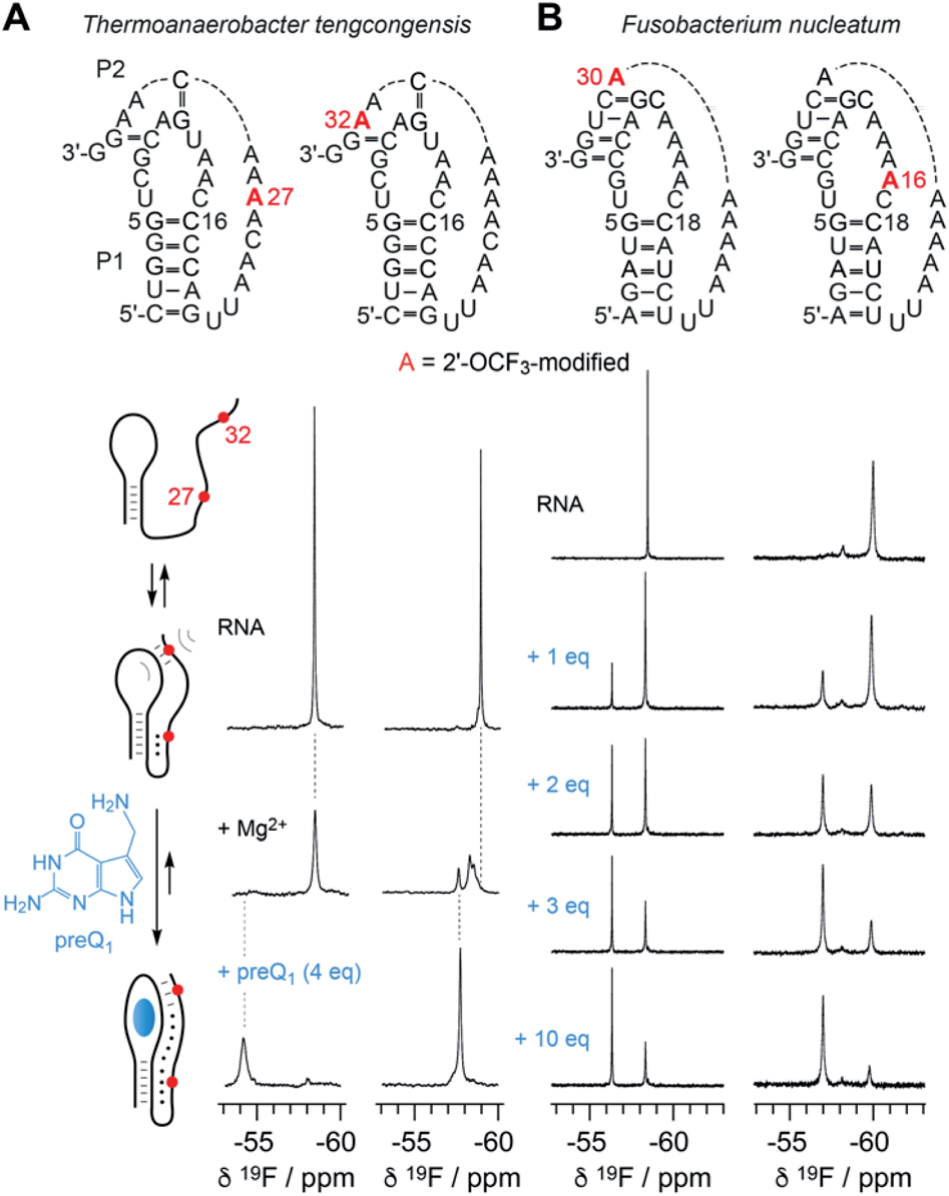
NMR spectroscopic analysis of RNA-ligand binding. (A) Secondary structure of *Thermoanaerobacter tengcongensis* preQ_1_-I riboswitch with 2′-OCF_3_ position indicated in red (top); schematic folding model (left) of aptamer-assisted RNA pseudoknot formation, and stabilization through binding of the small molecule 7-aminomethyl-7-deazaguanine (preQ_1_) indicated in blue; ^19^F NMR (564 MHz) spectra of RNA in response to Mg^2+^ and preQ_1_ addition (right). Conditions: c(RNA) = 0.14 mM, 15 mM sodium cacodylate, 25 mM NaCl, pH 6.5, 297 K; additions: c(Mg^2+^) = 2.0 mM; followed by c(preQ_1_) equivalents as indicated. (B) Secondary structure of *Fusobacterium nucleatum* preQ_1_-I riboswitch with two distinct 2′-OCF_3_ positions in red (top). ^19^F NMR (564 MHz) spectra of RNA in response to preQ_1_ titrations (right). Conditions: c(RNA) = 0.3 mM, 25 mM sodium cacodylate, pH 6.5, 298 K; additions: c(Mg^2+^) = 2.0 mM; followed by c(preQ_1_) equivalents as indicated.

The new 2′-OCF_3_–label is highly practical for NMR studies of RNA–ligand interactions. In Fig. 6, a comparison of the 7-aminomethyl-7-deazaguanine (preQ_1_) sensing class-I riboswitches from *Thermoanaerobacter tengcongensis* (*Tte*) (Fig. 6A) and *Fusobacterium nucleatum* (*Fn*) is illustrated (Fig. 6B).^56–58^ These RNAs adopt the same overall fold but differ in sequence and ligand affinity, the latter by about an order of magnitude (*K*_d_(*Tte*) ∼20 nM *versus K*_d_(*Fn*) ∼260 nmol).^57,58^ Moreover, for the *Tte* riboswitch, crystal structures at high resolution exist of both, the free and the preQ_1_-bound RNA,^57^ and therefore a solid foundation to explore the underlying conformational adaptions during ligand-induced folding is available.

At the left side of Fig. 6A, a secondary structure folding model for the preQ_1_ riboswitch is shown. The two RNAs with a single 2′-OCF_3_ label at either position 27 or 32 give a single ^19^F resonance in the absence of Mg^2+^ (Fig. 6A, spectra at top) which is consistent with a stem-loop fold and a conformationally flexible 3′-tail. In the presence of physiological concentrations of Mg^2+^ and a 3-fold excess of preQ_1_ ligand, both RNAs again provide a single – albeit broadened – resonance that is significantly shifted to low-field (Fig. 6A, spectra at bottom). This is consistent with a defined conformation of the preQ_1_–bound state. When only Mg^2+^ (and no preQ_1_) is added, the RNA becomes partially preorganized into a pseudoknot. Interestingly, the label at position 32 indicates heterogeneities within (or close to) the loop–tail interaction, reflected by the occurrence of three, partly overlapping ^19^F-resonances (Fig. 6A, spectrum at middle right). This suggests three distinct conformations in slow exchange, one of them closely resembling the ligand-bound state according to chemical shift comparison. We note that the observation of three states is consistent with a previous NMR study where the formation of individual (^15^N-labeled) base pairs of the same riboswitch system was tracked.^59^ We furthermore believe that two of the three folding states likely correspond to the conformational distinction seen in high-resolution crystal structures of free *versus* preQ_1_-bound *Tte* RNA. In the ligand-free pseudoknot form, A14 takes the position of the preQ_1_ ligand and is stacked between the two base pairs of G11–C30 and G5–C16.^57,59^ This observation can be rationalized by a conformational rearrangement from a solvent-exposed base C15 to a flipped-in conformation of C15 that becomes the Watson–Crick pairing partner of preQ_1_ in the ligand-bound pseudoknot RNA.^60^

Fig. 6B depicts NMR spectra of the titration of the *Fn* preQ_1_ riboswitch which binds preQ_1_ 13-fold weaker compared to the *Tte* counterpart. Furthermore, the loop size of the *Fn* RNA contains two additional nucleotides and the pseudoknot interaction allows formation of a continuous 4 bp double helix.^61^ We placed the 2′-OCF_3_ labels in different positions, one in the tail to sense pseudoknot formation, and the other one in the loop next to the cytosine that can form a Watson–Crick base pair with preQ_1_. Both labels allow to monitor the binding process. By increasing the concentration of preQ_1_ stepwise, an increasing fraction of the preQ_1_-RNA complex is observed, reflected in a second ^19^F resonance that is shifted to lower field. In contrast to the *Tte* RNA, the *Fn* riboswitch shows a significant population of unbound RNA, even at a ten-fold excess of ligand over RNA. Furthermore, a folding intermediate is also indicated by the appearance of a third resonance for the 2′-OCF_3_ A16 labeled RNA, although existing in a minor population only. Another interesting feature is that the line width of the ^19^F resonance of the label next to the pseudoknot double helix (A30) is rather small and the OCF_3_ group seems to be hardly hindered in rotational freedom compared to the other three labeling positions in the two riboswitch systems.

## Conclusions

Over the last decades, several ^19^F labels for nucleic acids NMR spectroscopy have been introduced, among them are single fluorine labels (such as pyrimidine 5-F,^16–20^ ribose 2′-F,^21–25^ and 4′-F,^3^), trifluoromethyl labels (such as pyrimidine 5-CF_3_,^26^ 4′-*C*-[(4-trifluoromethyl-1*H*-1,2,3-triazol-1-yl)methyl] ribose,^27^ 2′-SCF_3_,^28–30^ and very recently 8-CF_3_ guanine,^66^), and a nine-fluorine-atom label (in form of 5-[4,4,4-trifluoro-3,3-bis(trifluoromethyl)but-1-ynyl] 2′-deoxyuridine).^4^ Comparing them, the first subgroup is superior with respect to steric demands but suffers from low sensitivity and the need for proton decoupling;^21–25^ the opposite is true for the nine-fluorine-atom label which is highly sensitive but sterically demanding.^4^ Therefore, trifluoromethyl labels appear to be a good compromise.^26–30^ The pyrimidine 5-CF_3_ group, however, has been described to be chemically unstable during deprotection of synthetic nucleic acids, resulting in low yields and length limitation.^26^ Differently, the 2′-SCF_3_ group – although synthetically well accessible – strongly affects thermodynamic base pairing strength which is a drawback for applications that aim at the elucidation of folding pathways.^28–30^ All critical aspects and requirements are very well satisfied by the new 2′-OCF_3_ label as shown in this study; it therefore has potential to advance to the most broadly applicable fluorine label in NMR spectroscopy of nucleic acids. The 2′-OCF_3_ group possesses pronounced sensitivity and exhibits large chemical shift dispersion so that it is possible to distinguish distinct base sequences in double helical regions. Moreover, the ribose 2′ position guarantees principal synthetic accessibility to all of the four nucleosides. Additionally, the size of the OCF_3_ label matches the naturally occurring and widespread 2′-OCH_3_ group. For the investigation of naturally occurring 2′-OCH_3_ modification patterns in biologically meaningful settings, the 2′-OCF_3_ group will be an ideal candidate.

## Conflicts of interest

There are no conflicts to declare.

## Supplementary Material

SC-011-D0SC04520A-s001
