# Supplementary material for: 2′-O-Trifluoromethylated RNA – a powerful modification for RNA chemistry and NMR spectroscopy
Source: Chem Sci. 2020 Sep 24;11(41):11322–30. doi: 10.1039/d0sc04520a (PMC8162808; doi:10.1039/d0sc04520a)
Supplement: SC-011-D0SC04520A-s001 [file SC-011-D0SC04520A-s001.pdf]

Electronic Supplementary Material (ESI) for Chemical Science.
This journal is © The Royal Society of Chemistry 2020

Supporting Information

2′-*O*-Trifluoromethylated RNA – A powerful modification for RNA chemistry and NMR spectroscopy

Maximilian Himmelstoß,† Kevin Erharter,† Eva Renard,‡ Eric Ennifar,‡,* Christoph Kreutz,†,* Ronald Micura†,*

† University of Innsbruck, Institute of Organic Chemistry and Center for Molecular Biosciences (CMBI), Innrain 80-82,6020 Innsbruck, Austria
‡ Université de Strasbourg, Architecture et Réactivité de l'ARN - CNRS UPR 9002, Institut de Biologie Moléculaire et Cellulaire, 67000 Strasbourg, France

Contents

Supporting Methods

General procedures S02
Synthesis of 2'-OCF$_3$ adenosine phosphoramidite **A7** S05
Synthesis of 2'-OCF$_3$ cytidine phosphoramidite **C7a** S27
Synthesis of 2'-OCF$_3$ cytidine phosphoramidite **C7b** S49

Supporting Tables

Supporting Table 1 S65
Supporting Table 2 S66

Supporting Figures

Supporting Figure 1 S67
Supporting Figure 2 S68
Supporting Figure 3 S69
Supporting Figure 4 S70
Supporting Figure 5 S71
Supporting Figure 6 S72
Supporting Figure 7 S73

References S74

General procedures

Materials

Reagents were purchased in the highest available quality from commercial suppliers (Merck / Sigma-Aldrich, ABCR, VWR, ChemGenes, CarboSynth) and used without further purification. All reactions were carried out under argon atmosphere, unless otherwise noted. Analytical thin-layer chromatography (TLC) was performed on Macherey-Nagel Polygram® SIL G/UV_{254} plates. Silica gel 60 (0.04 – 0.06 mm) for column chromatography was purchased from Macherey-Nagel.

NMR measurements of compounds

^{1}H, ^{13}C, ^{19}F and ^{31}P spectra were recorded on a Bruker Ultrashield™ 400 Plus spectrometer. Chemical shifts (δ) are reported relative to tetramethylsilane (TMS), referenced to the residual solvent signal (DMSO-d_6: 2.50 ppm for ^{1}H and 39.52 ppm for ^{13}C spectra; $CDCl_3$: 7.26 ppm for ^{1}H and 77.16 ppm for ^{13}C spectra). The following abbreviations were used to denote multiplicities: s = singulet, d = doublet, t = triplet, q = quadruplet, m = multiplet, b = broad. Signal assignments are based on ^{1}H-^{1}H-COSY, ^{1}H-^{13}C-HSQC, ^{1}H-^{13}C-HMBC experiments.

High-resolution mass spectrometry of compounds

High resolution mass spectra were recorded in positive ion mode on a Thermo Scientific Q Exactive Orbitrap, ionized via electrospray at 3.7 kV spray voltage.

RNA solid-phase synthesis

Standard phosphoramidite chemistry was applied for RNA strand elongation and incorporation of 2'-OCF_3 modified nucleoside phosphoramidites: 2'-*O*-TOM standard RNA nucleoside phosphoramidite building blocks and 2'-*O*-TBDMS 1000 Å CPG solid support were purchased from ChemGenes. All oligonucleotides were synthesized on an ABI 391 or ABI 392 Nucleic Acid Synthesizer following standard methods: detritylation (90 sec) with dichloroacetic acid/1,2-dichloroethane (4/96); coupling (5.0 min) with phosphoramidites/acetonitrile (100 mM, 200 µL) and benzylthiotetrazole/acetonitrile (300 mM, 500 µL); capping (2 x 25 sec) with Cap A/Cap B (1/1), Cap A: 4-(dimethylamino)pyridine/acetonitrile (500 mM), Cap B: acetic anhydride/*sym*-collidine/acetonitrile (2/3/5); oxidation (60 sec) with iodine (20mM) in tetrahydrofuran/pyridine/H_2O (35/10/5). Solutions of phosphoramidites and tetrazole were dried over activated molecular sieves (3 Å) overnight.

Deprotection of natural and 2'-OCF_3 modified RNA

Solid support was treated with either methylamine/ethanol (33 %, 0.7 mL) and methylamine/H_2O (40 %, 0.7 mL) for 6 hours at 37 °C (EMAM) or ammonia/H_2O (28 %, 0.7 mL) and methylamine/H_2O (40 %, 0.7 mL) for 30 minutes at 65 °C (AMA). Supernatant was removed and solid support was washed thrice with tetrahydrofuran/H_2O (1/1). Combined supernatant and washings were evaporated to dryness and the residue was dissolved in a solution of tetrabutylammonium fluoride in tetrahydrofuran (1.0 M, 1.5 mL) and incubated for 16 hours at 37 °C for removal of 2'-O-silyl protecting groups. The reaction was quenched by addition of tetraethylammonium acetate/H_2O (1.0 M, 1.5 mL, pH 7.4). The solution was

reduced to one third of the original volume and desalted with size-exclusion column chromatography (GE Healthcare, HiPrep™ 26/10 Desalting; Sephadex G25) eluting with H_2O; collected fractions were evaporated and the RNA dissolved in H_2O (1 mL) for immediate use or storage at –20 °C.

Purification of natural and 2'-OCF$_3$ modified RNA

Crude RNA was purified by anion exchange chromatography on a semipreparative Dionex DNAPac® PA-100 column (9 mm x 250 mm) at 80 °C with 1 mL/min flow rate (Eluent A: 6 M urea, 25 mM Tris·HCl, pH 8.0; Eluent B: 500 mM $NaClO_4$, 6 M urea, 25 mM Tris·HCl, pH 8.0). Fractions containing RNA were diluted with 0.1 M triethylammonium bicarbonate solution, loaded on a C18 SepPak Plus® cartridge (Waters/Millipore), washed with H_2O and eluted with acetonitrile/H_2O (1/1).

HPLC analysis and quantification of natural and 2'-OCF$_3$ modified RNA

Analysis of crude and purified RNA was performed by anion exchange chromatography on a Dionex DNAPac® PA-100 column (4 mm x 250 mm) at 80 °C with flow rate of 1 mL/min. For RNA shorter or equal to 15 nucleotides, a gradient of 0 – 40 % B in 30 minutes and for RNA longer than 15 nucleotides a gradient of 0 – 60 % B was used; Eluent A: 6 M urea, 25 mM Tris·HCl, pH 8.0; Eluent B: 500 mM $NaClO_4$, 6 M urea, 25 mM Tris·HCl, pH 8.0. HPLC traces were recorded at UV absorption at 260 nm. The RNA was quantified on an Implen P300 Nanophotometer.

Mass spectrometry of natural and 2'-OCF$_3$ modified RNA

RNA samples (3 µL) were diluted with 40 mM Na_2H_2(EDTA)/H_2O (5/4) for a total volume of 30 µL, injected onto C18 XBridge 2.5 µm (2.1 mm x 50 mm) at a flow rate of 0.1 mL/min and eluted with 0 – 100 % B gradient at 30 °C (Eluent A: 8.6 mM triethylamine, 100 mM 1,1,1,3,3,3-hexafluoroisopropanol in H_2O; Eluent B: methanol). RNA traces were analyzed on a Finnigan LCQ Advantage Max electrospray ionization mass spectrometer with 4.0 kV spray voltage in negative mode.

NMR measurements of natural and 2'-OCF$_3$ modified RNA

RNA samples were lyophilized as triethylammonium salts and dissolved either in 280 µL or 400 µL NMR buffer (15 mM $Na[AsO_2(CH_3)_2]\cdot 3H_2O$, 25 mM NaCl, 3 mM NaN_3, in D_2O or 9/1 H_2O/D_2O, pH 6.5) and transferred into restricted volume Shigemi tubes or standard 5 mm NMR tubes. Sample concentrations varied between 0.1 and 1 mM and experiments were run at 298 K unless otherwise stated. All NMR experiments were conducted on a Bruker 600 MHz Avance II+ NMR or a 700 MHz Avance Neo NMR both equipped with a Prodigy TCI probe.

1D ^{19}F-NMR spectra were typically acquired using the following parameters: spectral width 10 ppm, o1p -60 ppm, 32k complex data points. 128 scans were collected with a recycling delay of 1 s resulting in an experimental time of 4 minutes.

For the 2D ^{19}F-^{13}C HMQC experiments at natural ^{13}C abundance the following parameters were used: spectral width in the indirect ^{13}C dimension was set to 10 ppm, and the spectral width in the direct ^{19}F dimension was set to 10 ppm. A total of 64 complex points was collected

in the indirect ^{13}C dimension (acquisition time = 21 ms) and 1024 complex points were collected in the direct ^{19}F dimension (acquisition time = 91 ms). 768 scans were collected with a recycling delay of 1 s resulting in an experimental time of 16 h. The carrier frequency was placed at -58 ppm in the ^{19}F dimension and in the ^{13}C dimension at 120 ppm. The $^{1}J_{CF}$ coupling constant was set to 270 Hz.

The ^{19}F-^{19}F-EXSY experiment was conducted using a Bruker standard NOESY pulse sequence (*noesygpph*). The following parameters were used: spectral width in the indirect ^{19}F dimension was set to 10 ppm, and the spectral width in the direct ^{19}F dimension was set to 10 ppm. A total of 32 complex points was collected in the indirect ^{19}F dimension (acquisition time = 3 ms) and 1024 complex points were collected in the direct ^{19}F dimension (acquisition time = 91 ms). The carrier frequency was placed at -58 ppm in both ^{19}F dimensions. 64 scans were collected with a recycling delay of 1 s resulting in an experimental time of 1 h for each EXSY spectrum. The experiment was run at 11 mixing times ranging from 50 to 800 ms with repeat experiments at 150 and 500 ms. Spectral processing and peak integration were performed using Topspin 4.0.8. All subsequent steps were performed using in-house written software written in Matlab (The MathWorks, www.mathworks.com) according to an earlier published protocol.[1] Errors in the extracted rate constants were determined by Monte Carlo analysis where peak intensities were randomly modulated according to the signal to noise levels in the 2D correlation maps.

Crystallization of SRL-OCF$_3$ RNA

27 nt RNA fragments corresponding to *E. coli* 23 S rRNA sarcin-ricin loop (SRL) modified at position A2670 or C2667 with 2'-*O*-trifluoromethyl were used for crystallization. RNA was dissolved at a 190 µM concentration in a buffer made of Na EDTA (1 mM, pH 8.0) and Tris-HCl (10 mM, pH 8.0). The dissolved RNA was heated at 55 °C and cooled down to 10 °C using a temperature-controlled device equipped with a Peltier element. Crystals of A2670-modified RNA were grown as very fragile needles (monoclinic form) for about one month at 4 °C using vapor diffusion method by mixing one volume of RNA sample and one volume of reservoir buffer composed of NaCl (80 mM), KCl (12 mM), $MgCl_2$ (20 mM), $Na[AsO_2(CH_3)_2]\cdot 3H_2O$ (40 mM, pH 7.0), MPD (35 %), and spermine tetrahydrochloride (12 mM). Crystals were flash-frozen in liquid ethane without further cryoprotection. Crystals of C2667-modified RNA were grown at 20 °C as platelets chunks (tetragonal form) and cryoprotected as described previously,[2] and finally flash-frozen in liquid ethane for data collection. The collection of X-ray diffraction data has been done on the X06DA beamline at the SLS synchrotron, Villigen, Switzerland. Processing of the data was done with the XDS Package[3] and the structure was solved by molecular replacement with MOLREP[4] using the related PDB ID 3DVZ unmodified SRL RNA model. Several crystals from the tetragonal form were discarded due to strong merohedral twinning. Both structures were refined with the PHENIX package.[5] Models were built using Coot.[6] A slight kink in the RNA backbone is observed close to the modification in all three molecules from the asymmetric unit of the monoclinic form (Supporting Figure 4). Coordinates have been deposited with the PDB database (PDB ID 6ZYB for C2667-modified SRL and 6ZXZ for A2670-modified SRL).

Synthesis of 2'-OCF_3 adenosine phosphoramidite A7

3',5'-*O*-(1,1,3,3-Tetraisopropyldisiloxane-1,3-diyl)adenosine

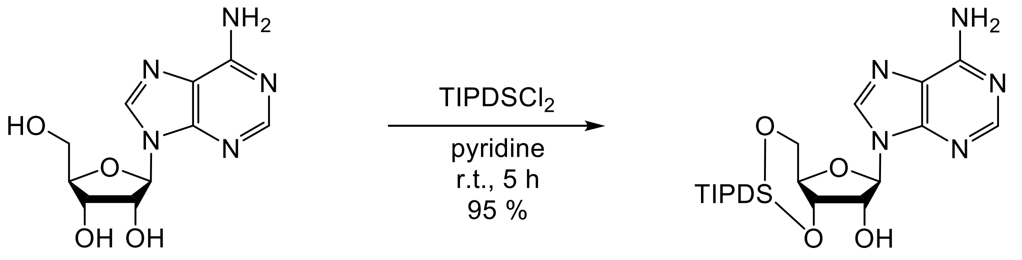

A1 **A2**

Adenosine (2.50 g, 9.35 mmol) was coevaporated two times with pyridine, suspended in dry pyridine (30 mL) and treated dropwise with 1,3-dichloro-1,1,3,3-tetraisopropyl disiloxane ($TIPDSCl_2$, 3.29 mL, 3.25 g, 10.3 mmol). After 5 hours, TLC showed complete conversion of starting material and the reaction was quenched with methanol (1 mL), evaporated under reduced pressure and coevaporated two times with toluene. The residue was dissolved in dichloromethane, washed with citric acid solution (5 %) and saturated bicarbonate solution, dried over sodium sulfate and evaporated. Crude product was purified by column chromatography on silica gel and eluted with 2 - 6 % methanol in dichloromethane. Yield: 4.54 g of **A2** (95 %) as white foam. TLC: (CH_2Cl_2/methanol, 9/1) R_F = 0.47. ESI-MS: (m/z) $[M+H]^+$ calcd. 510.2562; found: 510.2540. ^{1}H-NMR (400 MHz, DMSO-*d*$_6$, 25 °C): δ 1.03 (28H, s, ***H$_3$***C-, ***H***-C(TIPDS)), 3.92 (1H, q, J=4.97 Hz, ***H(a)***-C(5')), 4.00 (1H, m, J=2.82 Hz, ***H***-C(4')), 4.06 (1H, q, J=5.28 Hz, ***H(b)***-C(5')), 4.52 (1H, t, J=4.82 Hz, ***H***-C(2')), 4.79 (1H, q, J=4.51 Hz, ***H***-C(3')), 5.61 (1H, d, J=4.60 Hz, ***H***O-C(2')), 5.87 (1H, s, ***H***-C(1')), 7.32 (1H, s, ***H$_2$***N-C(6)), 8.07 (1H, s, ***H***-C(2)), 8.21 (1H, s, ***H***-C(8)). ^{13}C-NMR (100 MHz, DMSO-*d*$_6$, 25 °C): δ 12.87 (4C, q, J=22.37 Hz, -***C***H-(TIPDS)), 17.55 (8C, m, J=8.91 Hz,***C***H$_3$-(TIPDS)), 61.28 (1C, s, ***C***(5')), 70.30 (1C, s, ***C***(3')), 74.13 (1C, s, ***C***(2')), 81.23 (1C, s, ***C***(4')), 89.81 (1C, s, ***C***(1')), 119.74 (1C, s, ***C***(5)), 139.67 (1C, s, ***C***(8)), 149.09 (1C, s, ***C***(4)), 152.95 (1C, s, ***C***(2)), 156.57 (1C, s, ***C***(6)).

^{1}H-NMR (400 MHz, DMSO-d_6, 25 °C) of compound **A2**

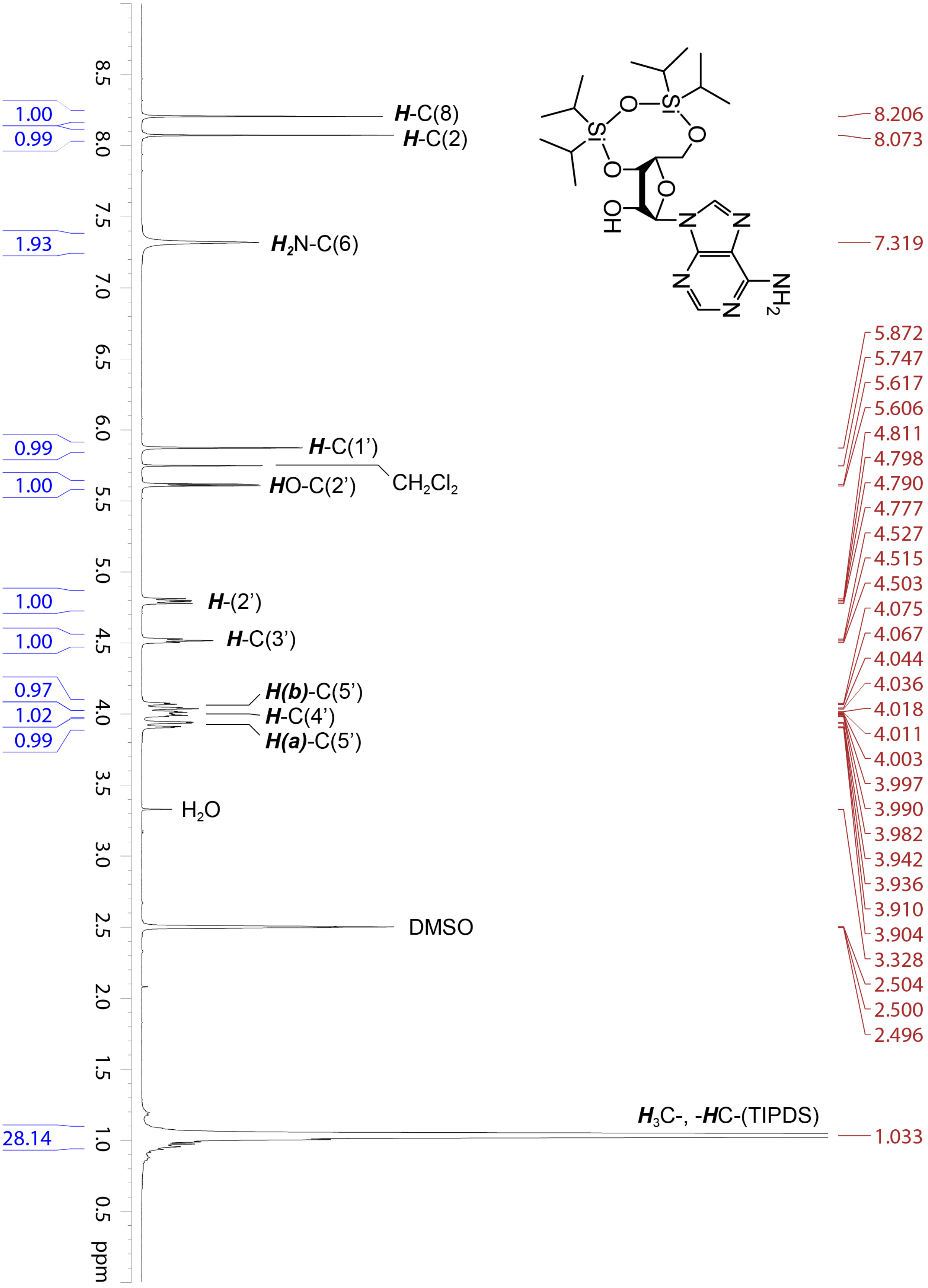

^{13}C- NMR (100 MHz, DMSO-d_6, 25 °C) of compound **A2**

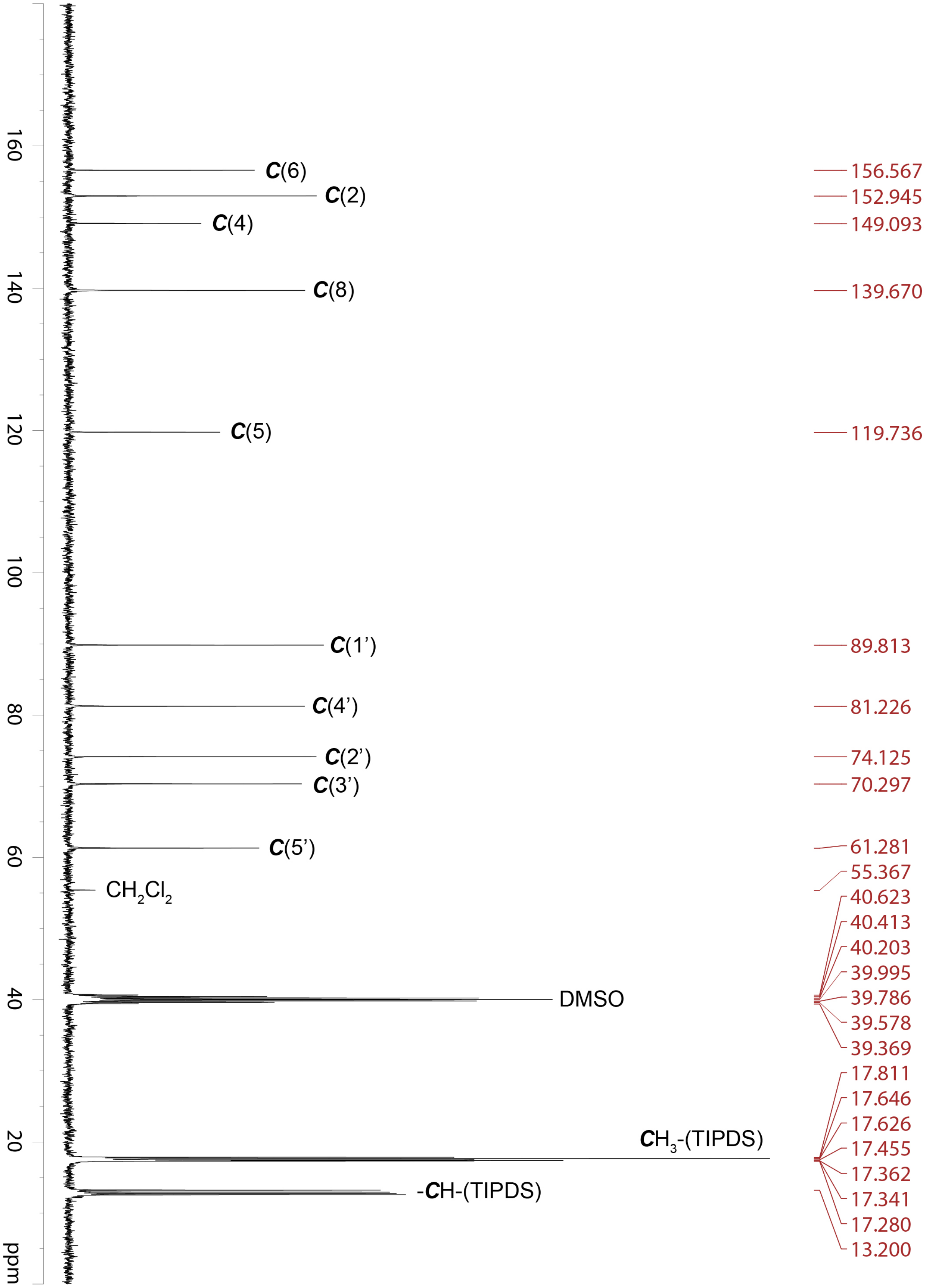

2'-*O*-[(Methylthio)thiocarbonyl]-3',5'-*O*-(1,1,3,3-tetraisopropyldisiloxane-1,3-diyl)adenosine

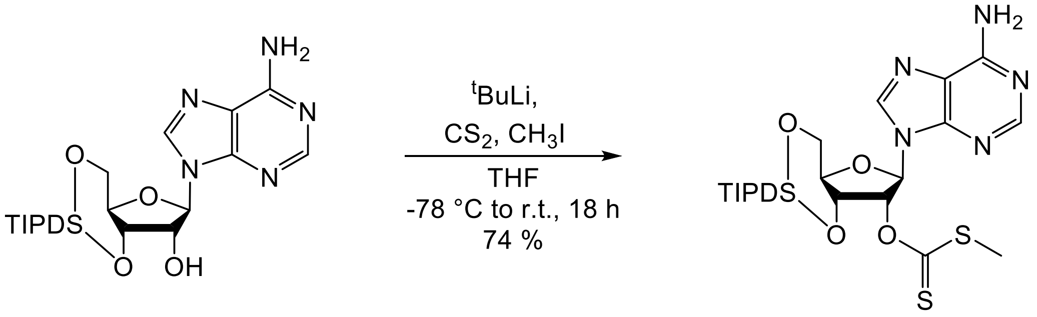

A2 **A3**

Compound **A2** (4.46 g, 8.74 mmol) was dissolved in anhydrous THF (35 mL), cooled to -78 °C and treated with *tert*-butyllithium in pentane solution (1.7 M, 6.43 mL, 700 mg, 10.9 mmol). After ten minutes, carbon disulfide (4.73 mL, 5.99 g, 78.7 mmol) was added, resulting in a dark colored mixture. After stirring for 1 h at -78 °C, methyl iodide (1.63 mL, 3.72 g, 26.2 mmol) was added and the reaction mixture was allowed to warm to room temperature overnight. Upon completion, dichloromethane (200 mL) was added, organic phases were washed with saturated bicarbonate solution, water and brine, dried over sodium sulfate and evaporated. Crude product was purified by column chromatography on silica gel using 1 - 4 % methanol in dichloromethane as eluent. Yield: 3.89 g of **A3** (74 %) as yellow foam. TLC: (CH_2Cl_2/methanol, 19/1) R_F = 0.26. ESI-MS (m/z) $[M+H]^+$ calcd. 600.2160; found: 600.2136. ^{1}H-NMR (400 MHz, DMSO-d_6, 25 °C): δ 1.04 (28H, m, J=5.31 Hz, ***H**₃*C-, ***H***-C(TIPDS)), 2.62 (3H, s, ***H***$_3$C-S), 4.00 (3H, m, J=5.91 Hz, ***H***-C(4'), ***H***$_2$-C(5')), 5.48 (1H, q, J=4.55 Hz, ***H***-C(3')), 6.25 (1H, d, J=1.20 Hz, ***H***-C(1')), 6.67 (1H, q, J=2.28 Hz, ***H***-C(2')), 7.39 (2H, s, ***H***$_2$N-C(6)), 8.04 (1H, s, ***H***-C(2)), 8.26 (1H, s, ***H***-C(8)). ^{13}C-NMR (100 MHz, DMSO-d_6, 25 °C): δ 12.34 (4C, q, J=14.98 Hz, -***C***H-(TIPDS)), 16.98 (8C, m, J=9.18 Hz, ***C***H_3-(TIPDS)), 18.44 (1C, s, ***C***H_3-S), 60.86 (1C, s, ***C***(5')), 69.85 (1C, s, ***C***(3')), 81.22 (1C, s, ***C***(4')), 82.90 (1C, s, ***C***(2')), 86.29 (1C, s, ***C***(1')), 119.21 (1C, s, ***C***(5)), 140.56 (1C, s, ***C***(8)), 148.53 (1C, s, ***C***(4)), 152.51 (1C, s, ***C***(2)), 156.22 (1C, s, ***C***(6)), 214.80 (1C, s, -***C***S_2-).

^{1}H-NMR (400 MHz, DMSO-d_6, 25 °C) of compound **A3**

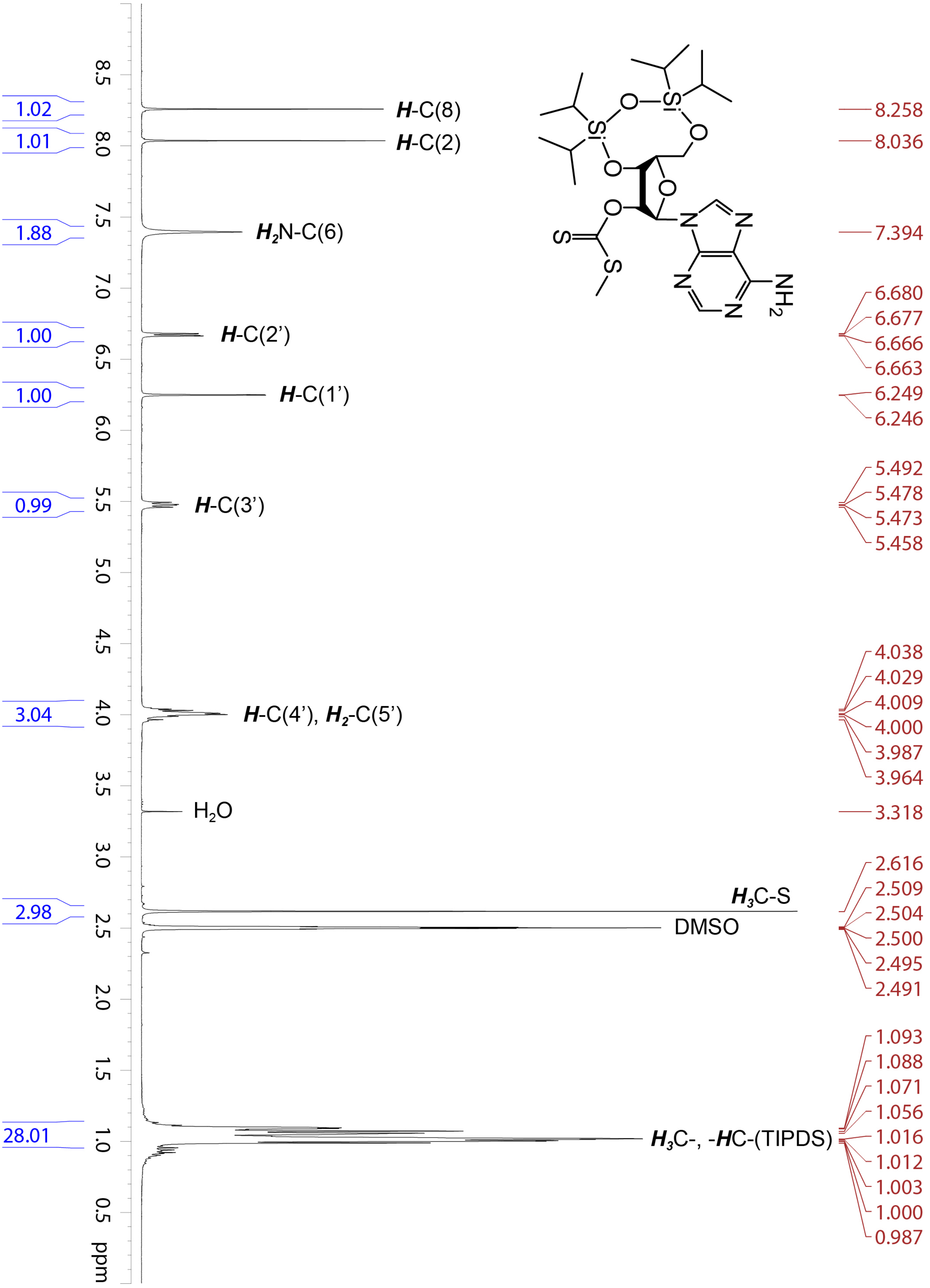

^{13}C-NMR (100 MHz, DMSO-d_6, 25 °C) of compound **A3**

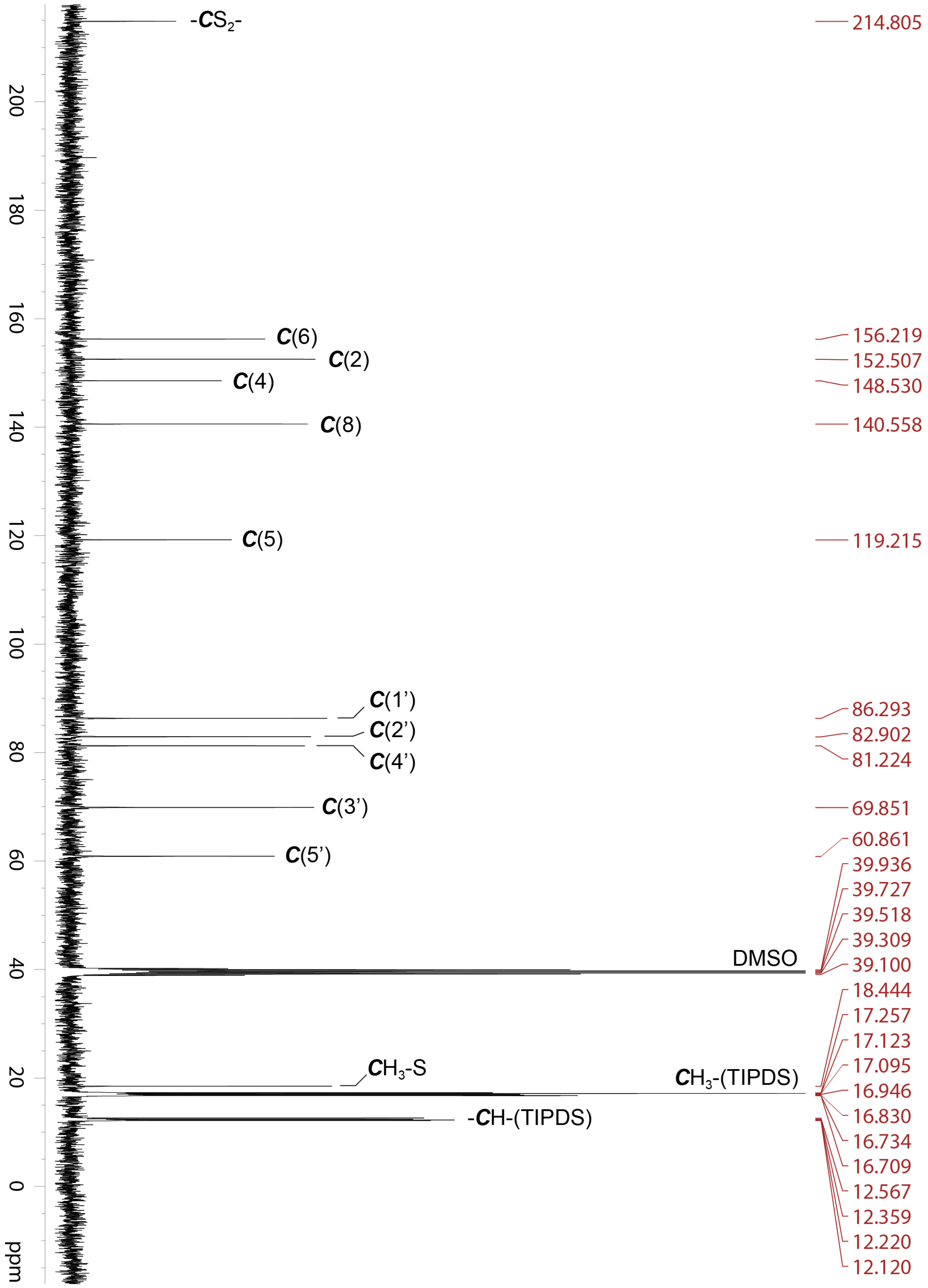

N^6-Benzoyl-2'-*O*-[(methylthio)thiocarbonyl]-3',5'-*O*-(1,1,3,3-tetraisopropyldisiloxane-1,3-diyl)adenosine

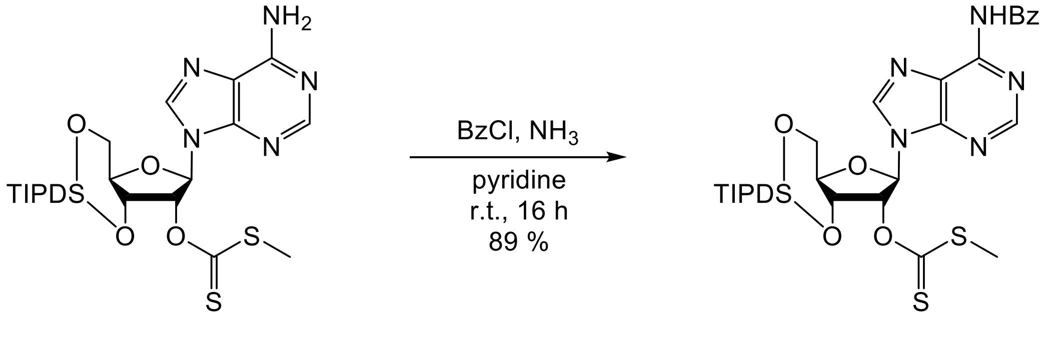

A3 **A4**

Compound **A3** (3.67 g, 6.11 mmol) was dissolved in dry pyridine (37 mL), treated with benzoyl chloride (BzCl, 1.77 mL, 2.15 g, 15.3 mmol) and stirred at ambient temperatures. After 16 hours, complete conversion of starting material was observable and the reaction was quenched with methanol (1 mL). To hydrolyze N^6,N^6-dibenzoylated byproducts back to the desired N^6-benzoylated derivative, tetrahydrofuran (25 mL) and ammonia in methanol (1.5 M, 25 mL) were added and stirring continued for 10 minutes. Upon completion, solvents were evaporated and the residue was coevaporated with toluene, dissolved in diethyl ether and washed with citric acid solution (5 %), saturated bicarbonate solution and water. Organic phases were combined, dried over sodium sulfate and concentrated under reduced pressure. Crude product was purified by column chromatography on silica gel, eluting with 1 - 3 % methanol in dichloromethane. Yield: 3.82 g of **A4** (89 %) as slight yellow foam. TLC: (CH_2Cl_2/methanol, 19/1): R_F = 0.50. ESI-MS: (m/z) $[M+H]^+$ calcd. 704.2423; found: 704.2395. ^{1}H-NMR (400 MHz, $CDCl_3$, 25 °C): δ 1.08 (28H, m, J=5.59 Hz, ***H**₃*C-, ***H***-C(TIPDS)), 2.61 (3H, s, ***H***$_3$C-S), 4.07 (1H, q, J=4.87 Hz, ***H(a)***-C(5')), 4.15 (1H, t, J=2.78 Hz, ***H***-(C4')), 4.18 (1H, dd, J=3.34, 10.78 Hz, ***H(b)***-C(5')), 5.38 (1H, q, J=4.73 Hz, ***H***-C(3')), 6.15 (1H, d, J=1.04 Hz, ***H***-C(1')), 6.56 (1H, q, J=2.09 Hz, ***H***-C(2')), 7.52 (2H, t, J=7.56 Hz, ***H***-C(benzoyl)), 7.59 (1H, m, J=5.83 Hz, ***H***-C(benzoyl)), 8.05 (2H, d, J=7.12 Hz, ***H***-C(benzoyl)), 8.15 (1H, s, ***H***-C(8)), 8.74 (1H, s, ***H***-C(2)), 9.37 (1H, b, ***H***N-C(6)). ^{13}C-NMR (100 MHz, $CDCl_3$, 25 °C): δ 13.02 (4C, q, J=15.17 Hz, -***C***H-(TIPDS)), 17.17 (8C, m, J=8.03 Hz, ***C***H_3-(TIPDS)), 19.18 (1C, s, ***C***H_3-S), 60.64 (1C, s, ***C***(5')), 69.66 (1C, s, ***C***(3')), 82.38 (1C, s, ***C***(4')), 83.09 (1C, s, ***C***(2')), 87.69 (1C, s, ***C***(1')), 123.63 (1C, s, ***C***(5)), 128.05 (1C, s, ***C***(benzoyl)), 128.39 (1C, s, ***C***(benzoyl)), 128.82 (1C, s, ***C***(benzoyl)), 130.06 (1C, s, ***C***(benzoyl)), 132.80 (1C, s, ***C***(benzoyl)), 133.34 (1C, s, ***C***(benzoyl)), 142.21 (1C, s, ***C***(8)), 149.88 (1C, s, ***C***(6)), 152.70 (1C, s, ***C***(2)), 215.18 (1C, s, -***C***S_2-).

^{1}H-NMR (400 MHz, $CDCl_3$, 25 °C) of compound **A4**

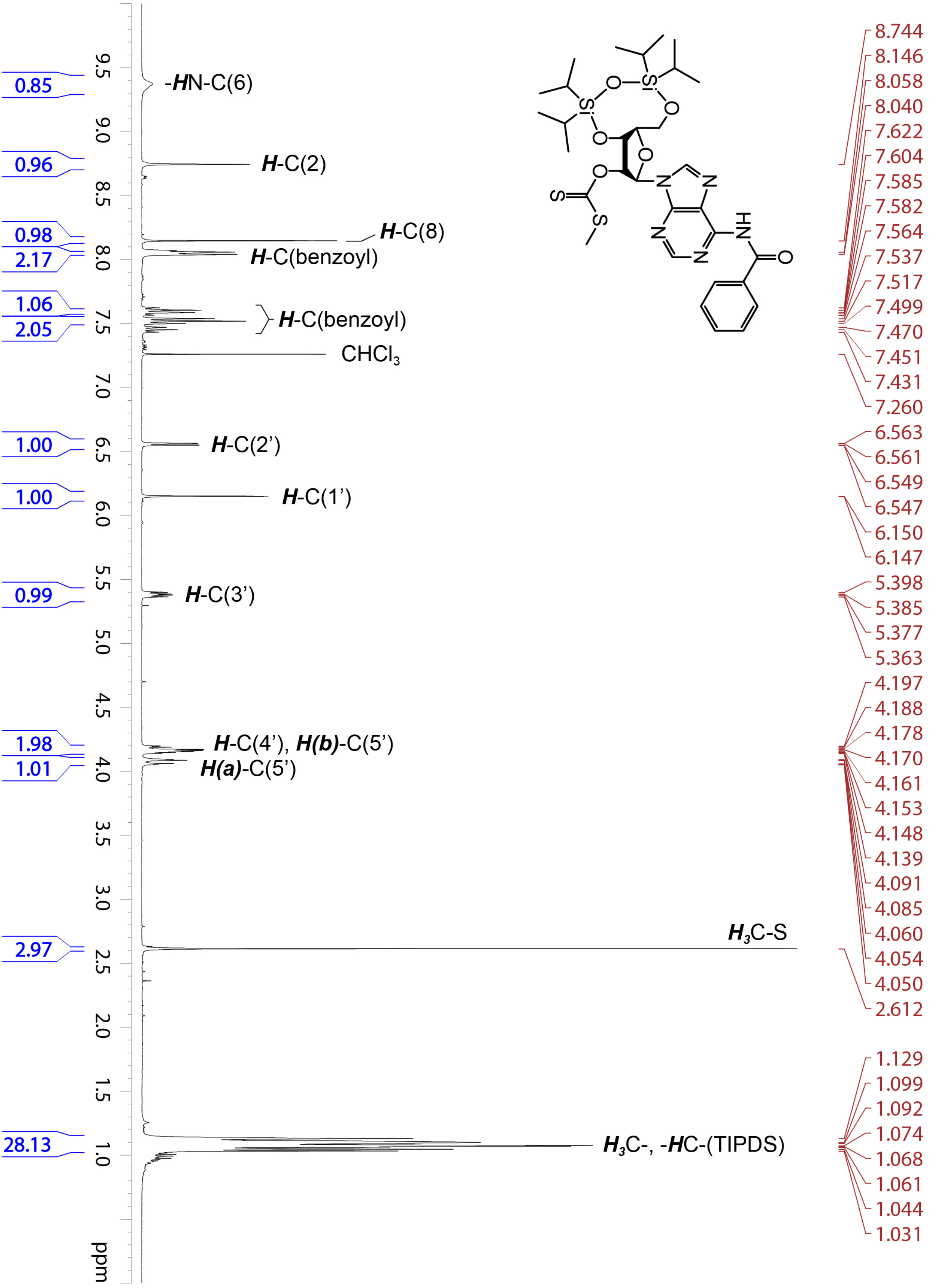

^{13}C-NMR (100 MHz, $CDCl_3$, 25 °C) of compound **A4**

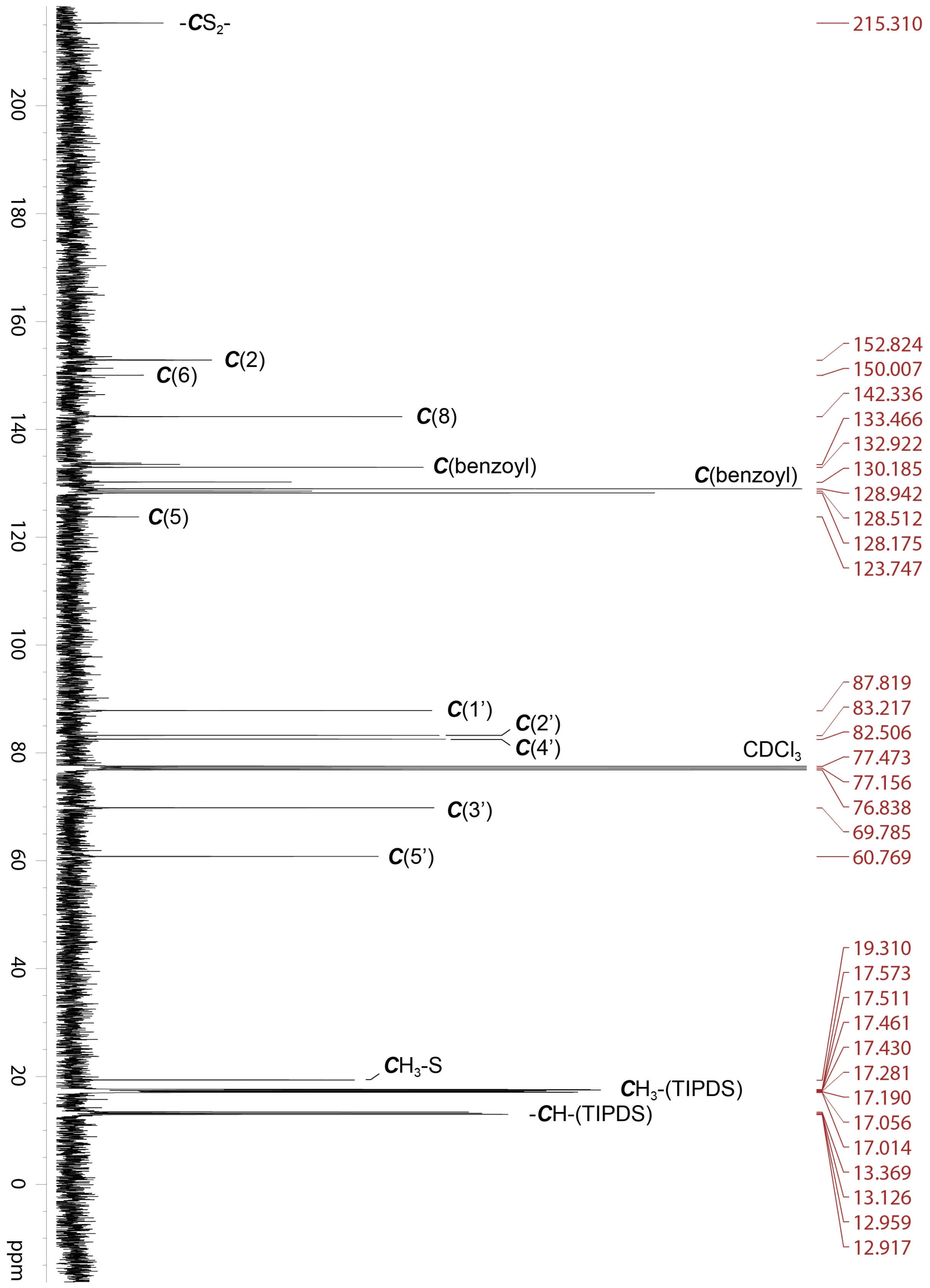

***N*[6]-Benzoyl-2'-*O*-(trifluoromethyl)adenosine**

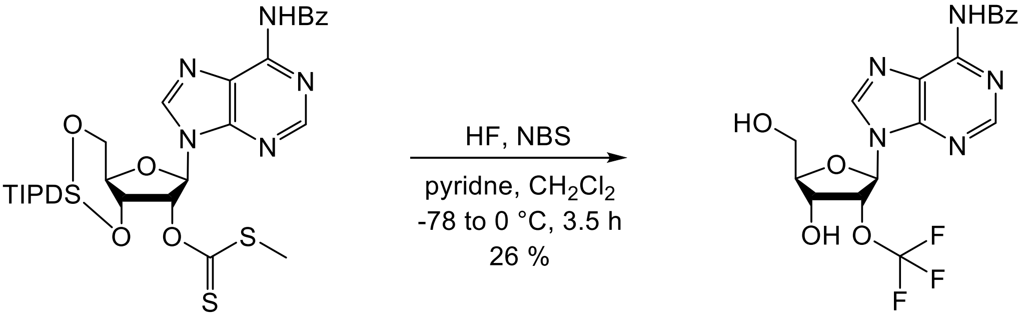

A4 **A5**

To a suspension of *N*-bromosuccinimide (NBS, 3.41 g, 19.2 mmol) in dry CH_2Cl_2 (20 mL) was added hydrogen fluoride in pyridine complex (70 % HF, 25.3 g, 23 mL) at -78 °C and a solution of compound **A4** (2.70 g, 3.84 mmol) in dry CH_2Cl_2 (30 mL). The reaction mixture was stirred at 0 °C for 3 hours and poured carefully onto a mixture of saturated sodium bicarbonate solution (250 mL) and sodium thiosulfate solution (10 %, 180 mL). Further bicarbonate solution was added until no more carbon dioxide evolution was observable and neutral pH was obtained. The mixture was extracted several times with chloroform/2-propanol (v/v, 3/1), organic phases were combined, dried over sodium sulfate and evaporated. Crude product was purified by column chromatography on silica gel using 1 - 5 % methanol in dichloromethane as eluent. Yield: 435 mg of **A5** (26 %) as white foam. TLC: (CH_2Cl_2/methanol, 9/1) R_F = 0.32. ESI-MS: (m/z) $[M+H]^+$ calcd. 440.1176; found: 440.1151. ^{1}H-NMR (400 MHz, DMSO-d_6, 25 °C): δ 3.69 (2H, md, J=4.32, 49.99 Hz, ***H*₂**-C(5')), 4.08 (1H, q, J=3.83 Hz, ***H***-C(4')), 4.50 (1H, q, J=4.77 Hz, ***H***-C(3')), 5.27 (1H, t, J=5.48 Hz, ***H***O-C(5')), 5.55 (1H, q, J=3.48 Hz, ***H***-C(2')), 6.02 (1H, d, J=5.60 Hz, ***H***O-C(3')), 6.40 (1H, d, J=5.48 Hz, ***H***-C(1')), 7.56 (2H, t, J=7.56 Hz, ***H***-C(benzoyl)), 7.65 (1H, t, J=7.36 Hz, ***H***-C(benzoyl)), 8.05 (2H, d, J=7.28 Hz, ***H***-C(benzoyl)), 8.77 (1H, s, ***H***-C(8)), 8.79 (1H, s, ***H***-C(2)), 11.26 (1H, b, ***H***N-C(6)). ^{13}C-NMR (100 MHz, DMSO-d_6, 25 °C): δ 60.57 (1C, s, ***C***(5')), 68.61 (1C, s, ***C***(3')), 78.65 (1C, s, ***C***(2')), 84.76 (1C, s, ***C***(1')), 85.79 (1C, s, ***C***(4')), 122.40 (1C, s, ***C***(5)), 125.68 (1C, s, -***C***F_3), 128.49 (4C, d, J=2.56 Hz, ***C***(benzoyl)), 132.51 (1C, s, ***C***(benzoyl)), 133.25 (1C, s, ***C***(benzoyl)), 142.87 (1C, s, ***C***(8)), 150.64 (1C, s, ***C***(4)), 151.96 (2C, d, J=4.38 Hz, ***C***(2), ***C***(6)), 165.61 (1C, s, ***C***O(benzoyl)). ^{19}F-NMR (376 MHz, DMSO-d_6, 25 °C): δ -57.51 (3F, s, $\boldsymbol{F_3}$C).

^{1}H-NMR (400 MHz, DMSO-d_6, 25 °C) of compound **A5**

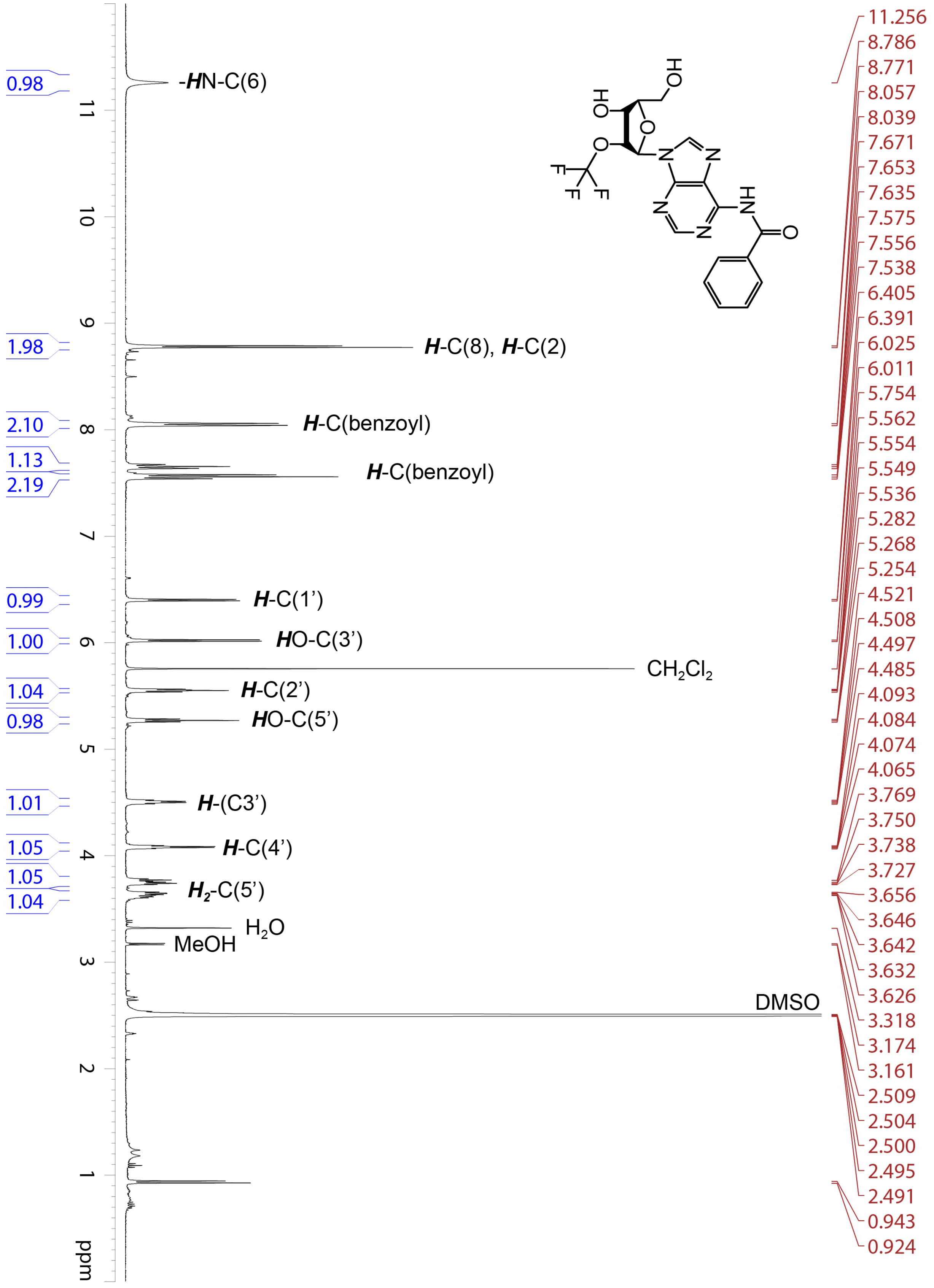

^{13}C-NMR (100 MHz, DMSO-d_6, 25 °C) of compound **A5**

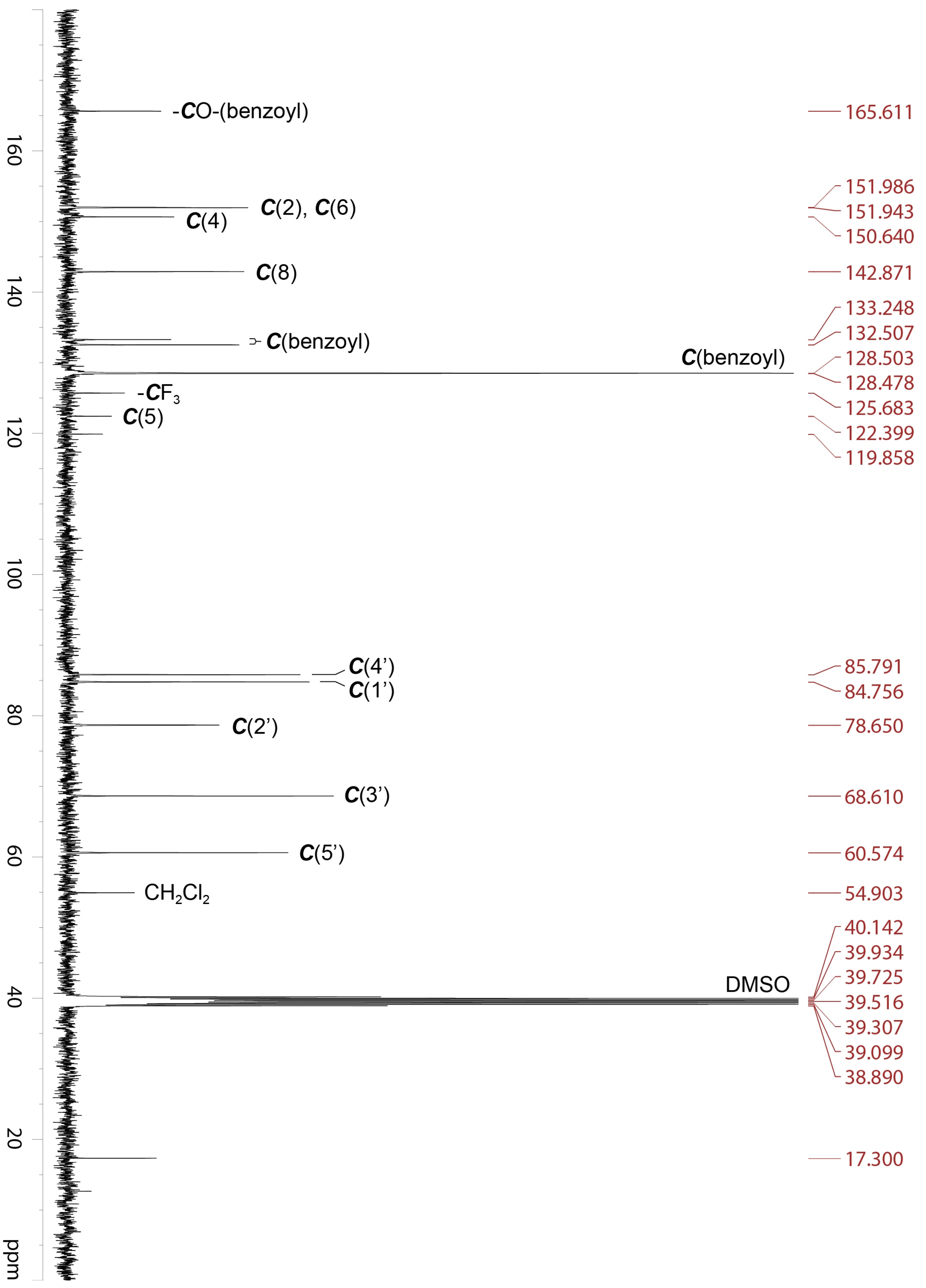

^{19}F-NMR (376 MHz, DMSO-d_6, 25 °C) of compound **A5**

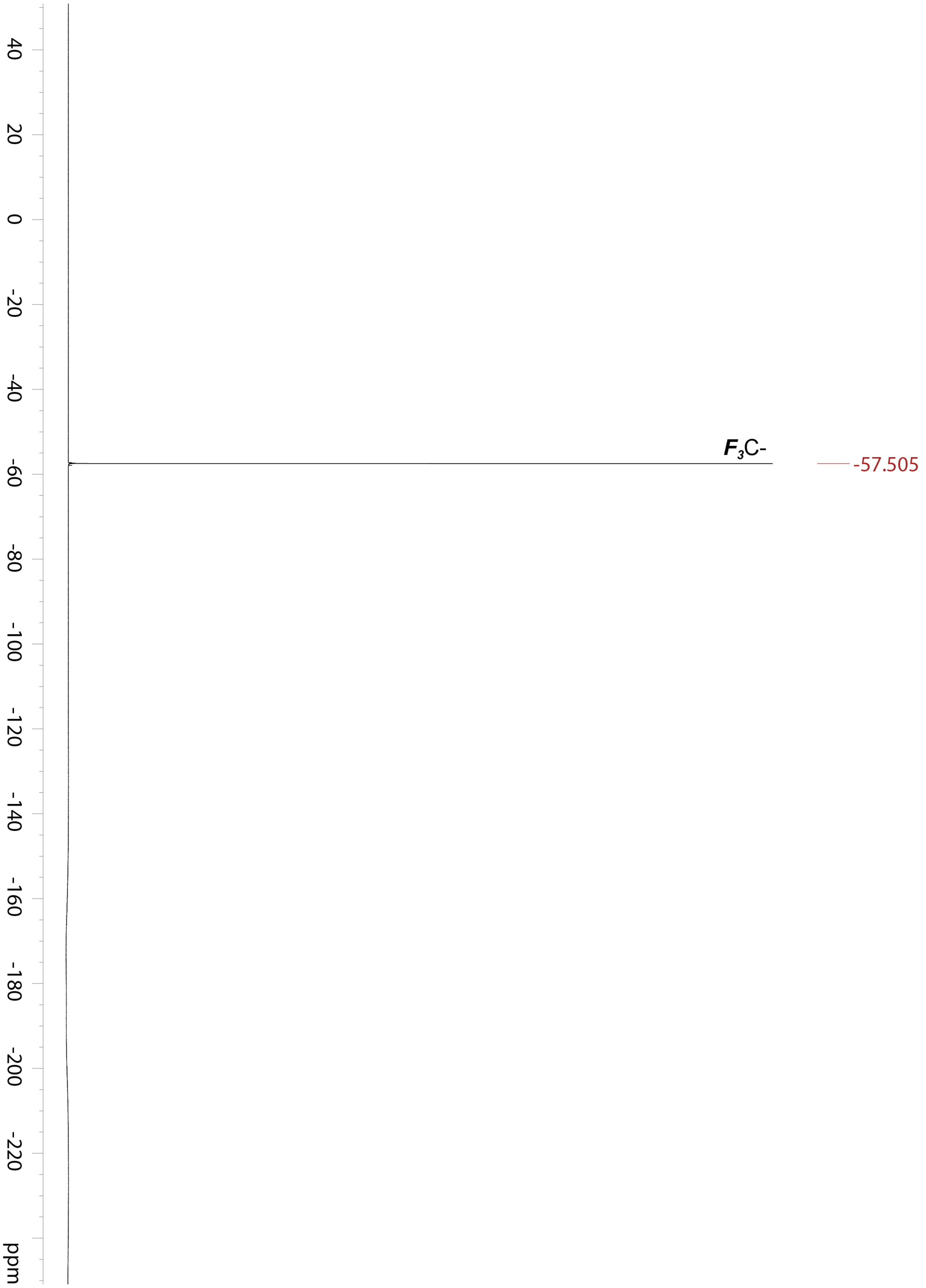

N^6-Benzoyl-5'-*O*-(4,4-dimethoxytrityl)-2'-*O*-(trifluoromethyl)adenosine

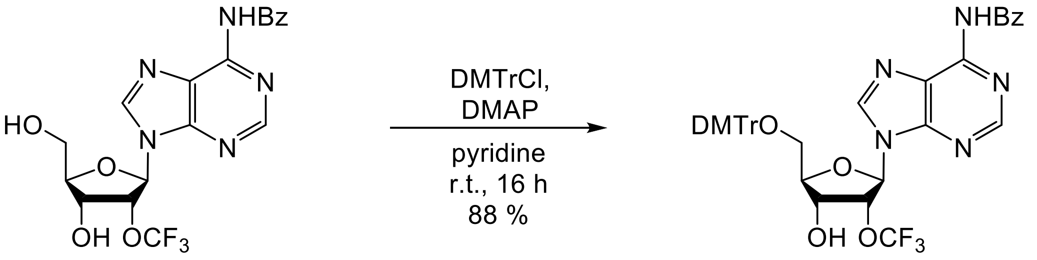

A5 **A6**

Compound **A5** (370 mg, 0.84 mmol) was dissolved in dry pyridine (5 mL) and treated with 4,4'-dimethoxytrityl chloride (DMTrCl, 428 mg, 1.26 mmol) and 4-dimethyl-aminopyridine (DMAP, 51.4 mg, 0.42 mmol) in four portions at ambient temperatures. The reaction mixture was stirred overnight, quenched with methanol (0.5 mL) and partitioned between diethyl ether and water. Organic phases were washed with citric acid solution (5 %), saturated sodium bicarbonate solution and brine, dried over sodium sulfate and concentrated. Crude product was purified by column chromatography using 1 - 4 % methanol in dichloromethane as eluent. Yield: 552 mg of **A6** (88 %) as white foam. TLC: (CH_2Cl_2/methanol, 9/1) R_F = 0.39. ESI-MS: (m/z) $[M+H]^+$ calcd. 742.2483; found: 742.2447. ^{1}H-NMR (400 MHz, $CDCl_3$, 25 °C): δ 3.52 (2H, ddd, J=3.65, 10.71, 57.49 Hz, ***H₂***-C(5')), 3.80 (6H, d, J=0.48 Hz, ***H₃***C-O(DMTr), 4.32 (1H, q, J=3.45 Hz, ***H***-C(4')), 4.76 (1H, t, J=3.66 Hz, ***H***-C(3')), 5.83 (1H, t, J=5.34 Hz, ***H***-C(2')), 6.33 (1H, d, J=5.88 Hz, ***H***-C(1')), 6.83 (4H, d, J=8.80 Hz, ***H***-C(DMTr)), 7.30 (7H, m, J=5.44 Hz, ***H***-C(DMTr)), 7.42 (2H, q, J=3.17 Hz, ***H***-C(DMTr)), 7.55 (2H, t, J=7.52 Hz, ***H***-C(benzoyl)), 7.64 (1H, t, J=7.38 Hz, ***H***-C(benzoyl)), 8.04 (2H, d, J=7.32 Hz, ***H***-C(benzoyl)), 8.13 (1H, s, ***H***-C(8)), 8.70 (1H, s, ***H***-C(2)), 9.04 (1H, s, ***H***N-C(6)). ^{13}C-NMR (100 MHz, $CDCl_3$, 25 °C): δ 55.38 (2C, s, ***C***H_3-O(DMTr)), 63.01 (1C, s, ***C***(5')), 70.73 (1C, s, ***C***(3')), 77.88 (1C, s, ***C***(2')), 84.11 (1C, s, ***C***(4')), 85.62 (1C, s, ***C***(1')), 87.11 (1C, s, ***C***(DMTr)), 113.38 (4C, s, ***C***(DMTr)), 123.52 (1C, s, ***C***(5)), 127.25 (1C, s, ***C***F_3), 127.99 (2C, s, ***C***(DMTr)), 128.10 (2C, s, ***C***(DMTr)), 128.26 (2C, s, ***C***(benzoyl)), 129.05 (2C, s, ***C***(benzoyl)), 130.20 (2C, d, J=2.53 Hz, ***C***(DMTr)), 133.01 (1C, s, ***C***(benzoyl)), 133.72 (1C, s, ***C***(benzoyl)), 135.47 (2C, d, J=3.11 Hz, ***C***(DMTr)), 142.04 (1C, s, ***C***(8)), 144.37 (1C, s, ***C***(DMTr)), 149.86 (1C, s, ***C***(4)), 151.82 (1C, s, ***C***(6)), 153.07 (1C, s, ***C***(2)), 158.81 (2C, s, ***C***(DMTr)), 164.65 (1C, s, ***C***O(benzoyl)). ^{19}F-NMR (376 MHz, $CDCl_3$, 25 °C): δ -59.09 (3F, s, ***F₃***C).

^{1}H-NMR (400 MHz, $CDCl_3$, 25 °C) of compound **A6**

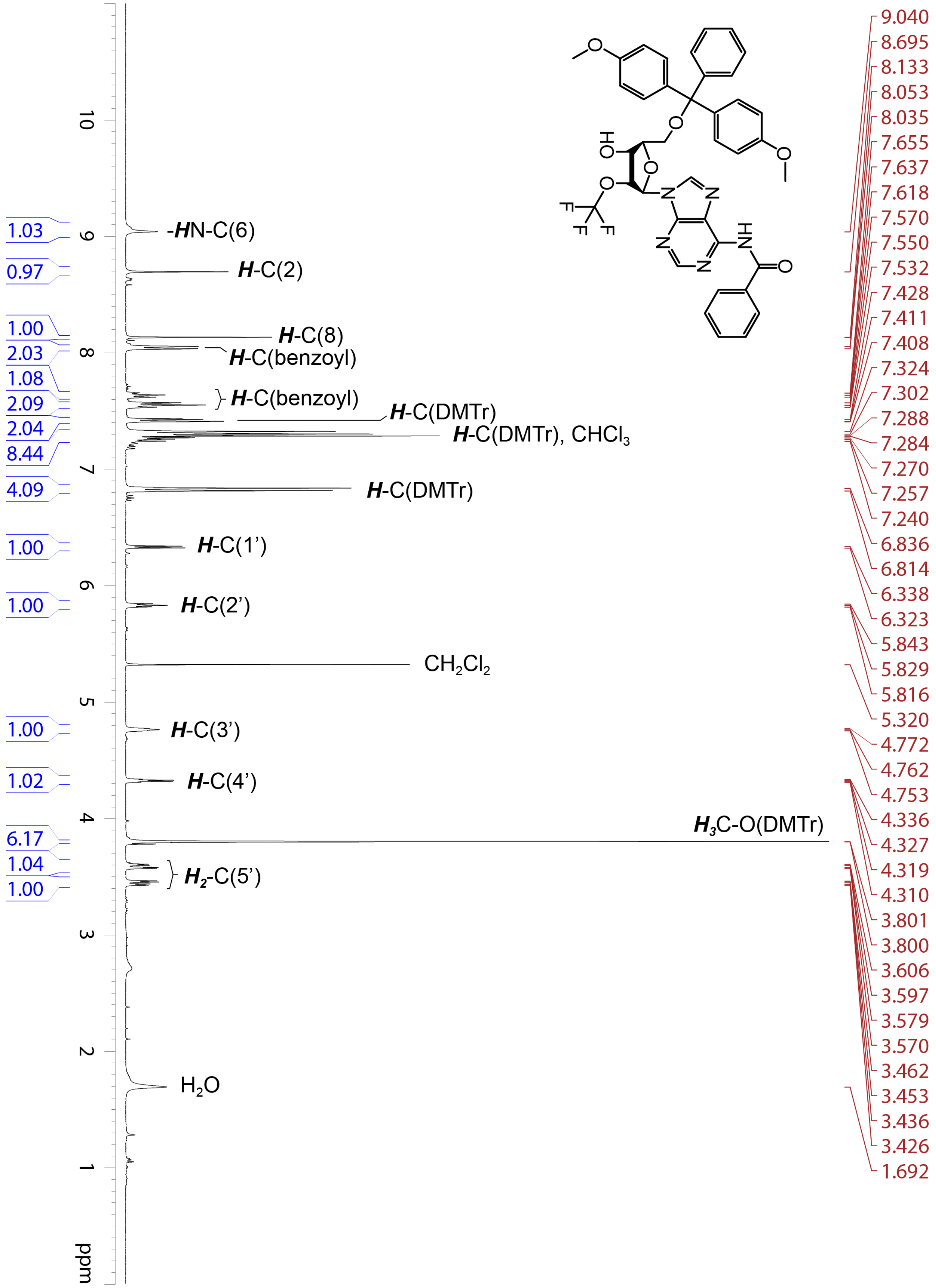

^{13}C-NMR (100 MHz, $CDCl_3$, 25 °C) of compound **A6**

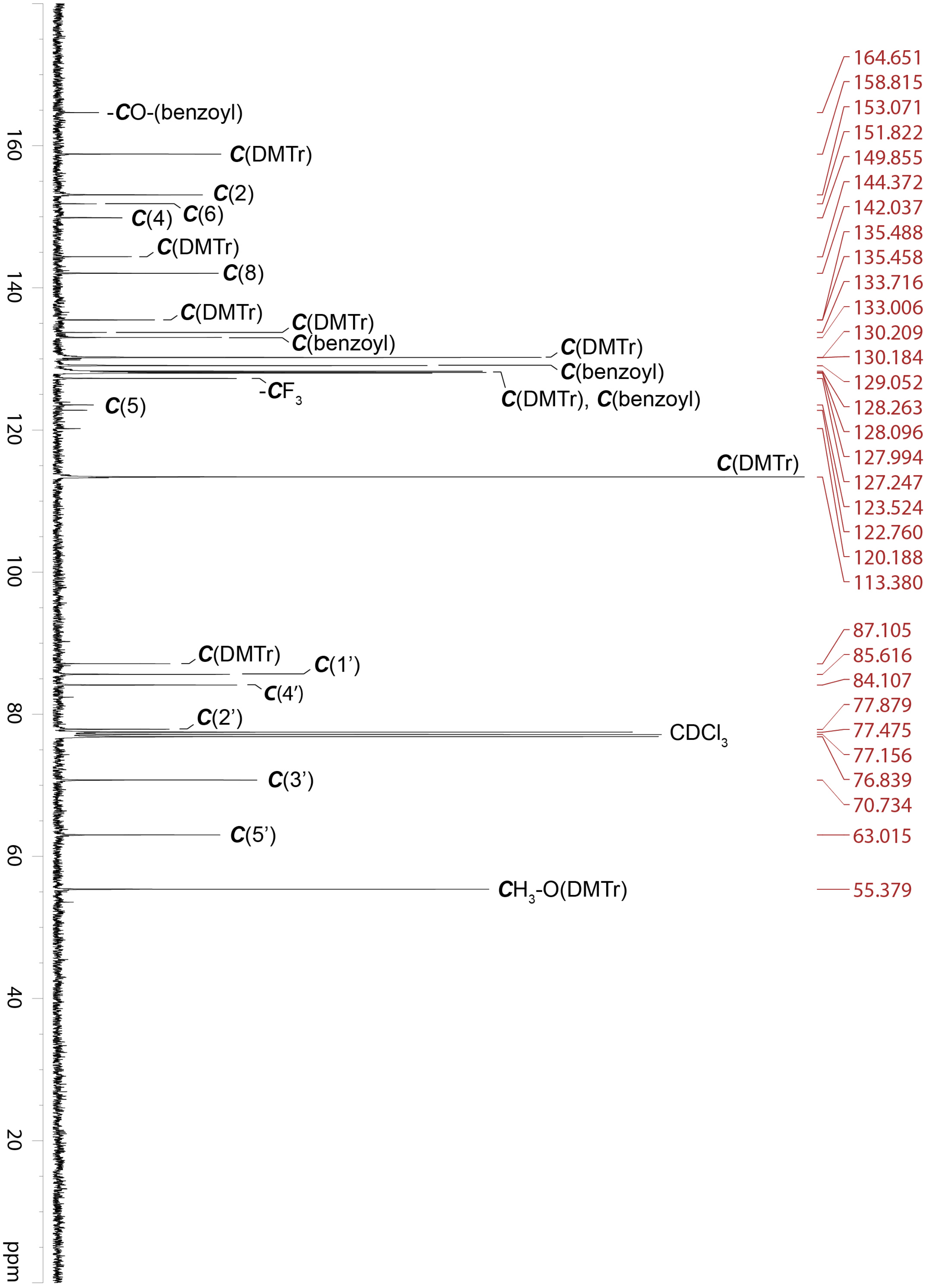

^{19}F-NMR (376 MHz, $CDCl_3$, 25 °C) of compound **A6**

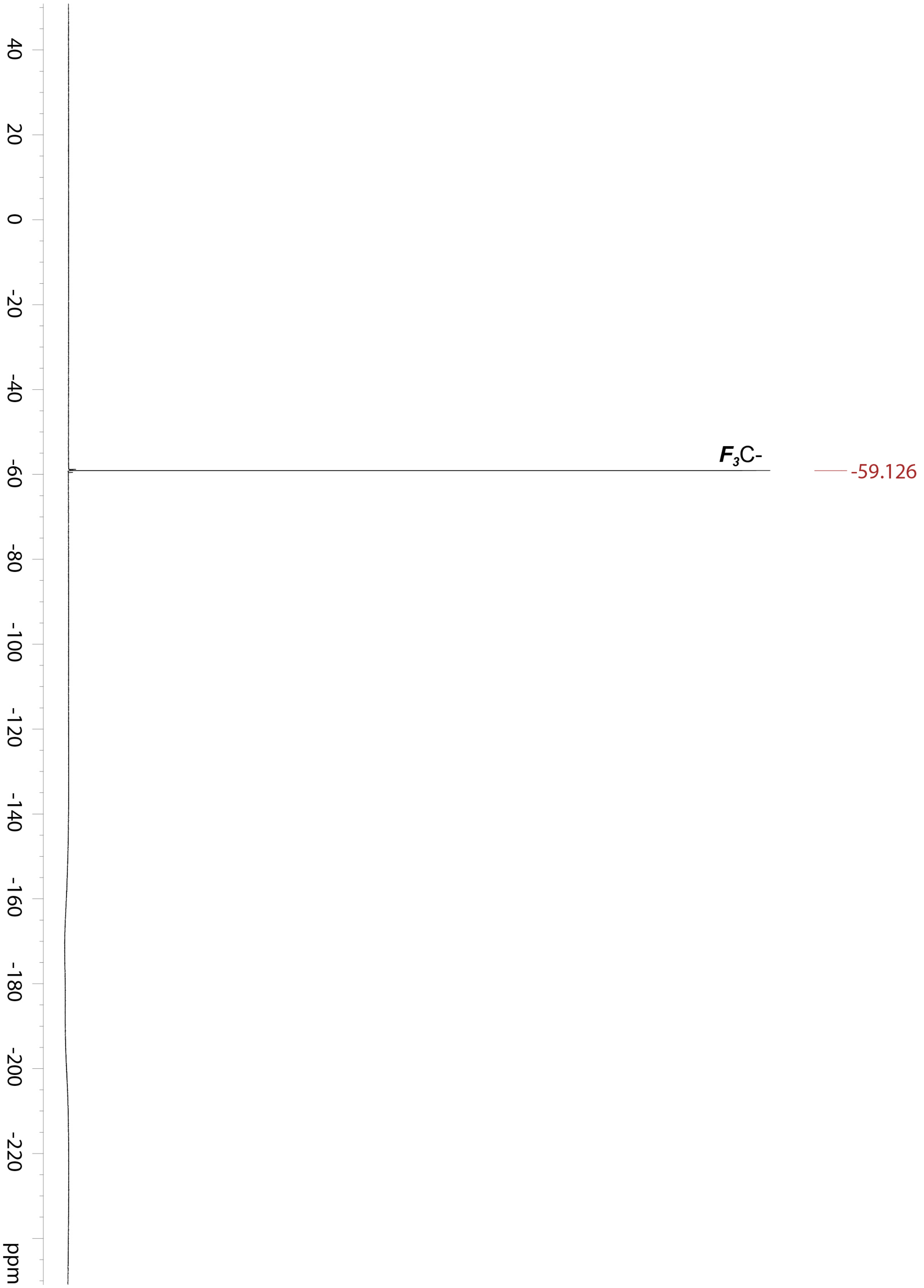

***N*^{6}-Benzoyl-2'-*O*-trifluoromethyl-5'-*O*-(4,4-dimethoxytrityl)adenosine 3'-(2-cyanoethyl)-*N,N*-diisopropylphosphoramidite**

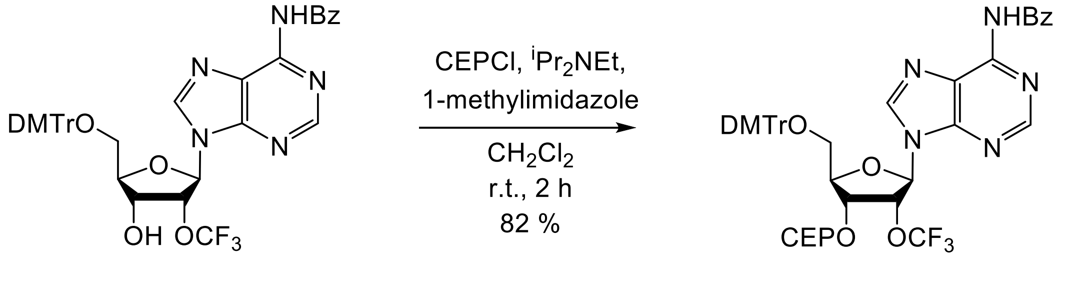

A6 **A7**

Compound **A6** (437 mg, 0.59 mmol) was dissolved in dry dichloromethane (3.8 mL) and treated with diisopropylethylamine (686 µL, 509 mg, 4.42 mmol), 2-cyanoethyl-*N,N*-diisopropylchlorophosphoramidite (CEPCl, 329 µL, 349 mg, 1.47 mmol) and 1-methylimidazole (23.5 µL, 24.2 mg, 0.30 mmol). The reaction mixture was stirred for 2 hours at ambient temperatures until TLC showed complete conversion of starting material. The reaction was quenched with methanol (0.5 mL) and all volatiles were evaporated. Crude product was purified by column chromatography on silica gel, eluting with 20 - 50 % ethyl acetate in hexanes + 2 % triethylamine. Yield: 453 mg of compound **A7** (82 %) as white foam and in a 3:5 ratio of diastereomers. TLC: (ethyl acetate/hexanes, 2/1) RF = 0.60. ESI-MS: (m/z) $[M+H]^+$ calcd. 942.3562; found: 942.3490. ^{1}H-NMR (600 MHz, $CDCl_3$, 25 °C): δ 1.15 (12H, dt, J=6.79, 40.08 Hz, ***H*$_3$**C-CNP), 2.52 (2H, dq, J=6.78, 129.37 Hz, -***H*$_2$**C-COP), 3.46 (2H, qd, J=4.71, 121.33 Hz, ***H*$_2$**-C(5')), 3.63 (2H, m, J=5.57 Hz, -***H***C-NP), 3.78 (6H, q, J=2.00 Hz, ***H*$_3$**C-O(DMTr)), 3.88 (2H, m, J=5.53 Hz, -***H*$_2$**C-OP), 4.40 (1H, dd, J=3.24, 53.60 Hz, ***H***-C(4')), 4.81 (1H, md, J=4.43, 43.00 Hz, ***H***-C(3')), 5.86 (1H, m, J=5.88 Hz, ***H***-C(2')), 6.29 (1H, dd, J=6.12, 32.65 Hz, ***H***-C(1')), 6.80 (4H, t, J=8.58 Hz, ***H***-C(DMTr)), 7.26 (7H, m, J=5.99 Hz, ***H***-C(DMTr)), 7.40 (2H, t, J=3.87 Hz, ***H***-C(DMTr)), 7.52 (2H, t, J=7.65 Hz, ***H***-C(benzoyl)), 7.61 (1H, t, J=7.35 Hz, ***H***-C(benzoyl)), 8.02 (2H, d, J=7.56 Hz, ***H***-C(benzoyl)), 8.14 (1H, d, J=8.94 Hz, ***H***-C(8)), 8.67 (1H, s, ***H***-C(2)), 9.01 (1H, b, ***H***N-C(6)). ^{13}C-NMR (100 MHz, $CDCl_3$, 25 °C): δ 20.27 (1C, dd, J=6.97, 20.24 Hz, -***C***H$_2$-COP), 24.55 (4C, q, J=6.16 Hz, ***C***H$_3$-CNP), 43.38 (2C, t, J=11.21 Hz, -***C***H-NP), 55.24 (2C, s, ***C***H$_3$-O(DMTr)), 58.44 (1C, dd, J=19.26, 88.84 Hz, -***C***H$_2$-OP), 62.57 (1C, s, ***C***(5')), 71.43 (1C, dd, J=17.05, 101.88 Hz, ***C***(3')), 76.46 (1C, d, J=82.00 Hz, ***C***(2')), 84.14 (1C, d, J=64.78 Hz, ***C***(4')), 85.75 (1C, d, J=16.74 Hz, ***C***(1')), 86.94 (1C, s, ***C***(DMTr)), 113.22 (4C, s, ***C***(DMTr)), 117.38 (1C, d, J=20.73 Hz, -***C***N), 123.49 (1C, s, ***C***(5)), 127.06 (1C, s, -***C***F$_3$), 127.89 (6C, d, J=8.46 Hz, ***C***(DMTr), ***C***(benzoyl)), 128.92 (2C, s, ***C***(DMTr)), 130.11 (4C, d, J=5.63 Hz, ***C***(DMTr), ***C***(benzoyl)), 132.84 (1C, s, ***C***(benzoyl)), 133.64 (1C, s, ***C***(benzoyl)), 135.38 (2C, d, J=10.10 Hz, ***C***(DMTr)), 142.05 (1C, s, ***C***(8)), 144.26 (1C, d, J=6.83 Hz, ***C***(DMTr)), 149.69 (1C, s, ***C***(4)), 151.70 (1C, s, ***C***(6)), 152.89 (1C, s, ***C***(2)), 158.66 (2C, s, ***C***(DMTr)), 164.49 (1C, s, -***C***O-(benzoyl). ^{19}F-NMR (376 MHz, $CDCl_3$, 25 °C): δ -59.09 (3F, dd, J=4.85, 104.01 Hz, ***F*$_3$**C-). ^{31}P-NMR (162 MHz, $CDCl_3$, 25 °C): δ 152.01 (1P, qd, J=4.74, 32.84 Hz, ***P***OC(3')).

^{1}H-NMR (600 MHz, $CDCl_3$, 25 °C) of compound **A7**

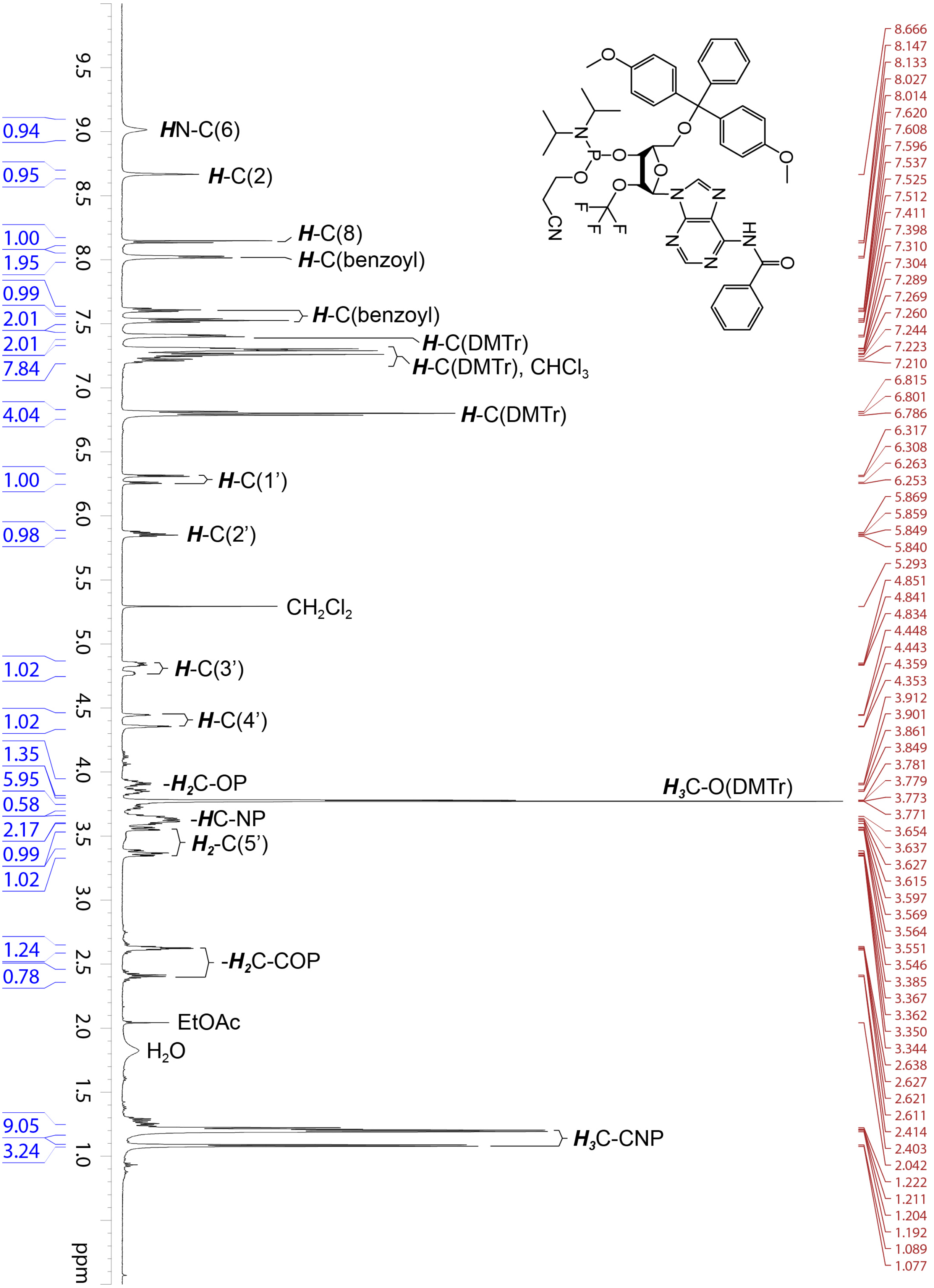

^{13}C-NMR (100 MHz, $CDCl_3$, 25 °C) of compound **A7**

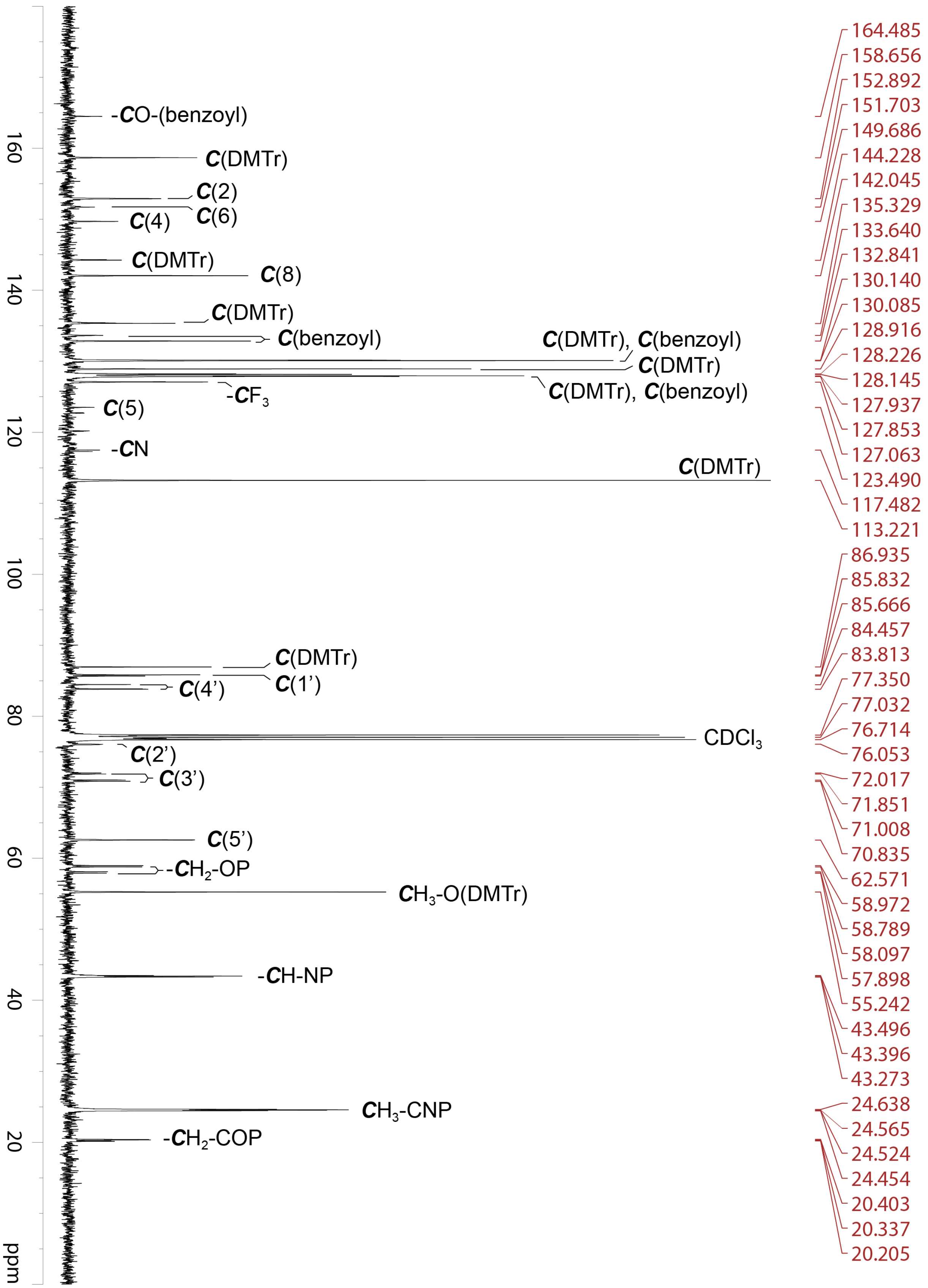

^{19}F-NMR (376 MHz, $CDCl_3$, 25 °C) of compound **A7**

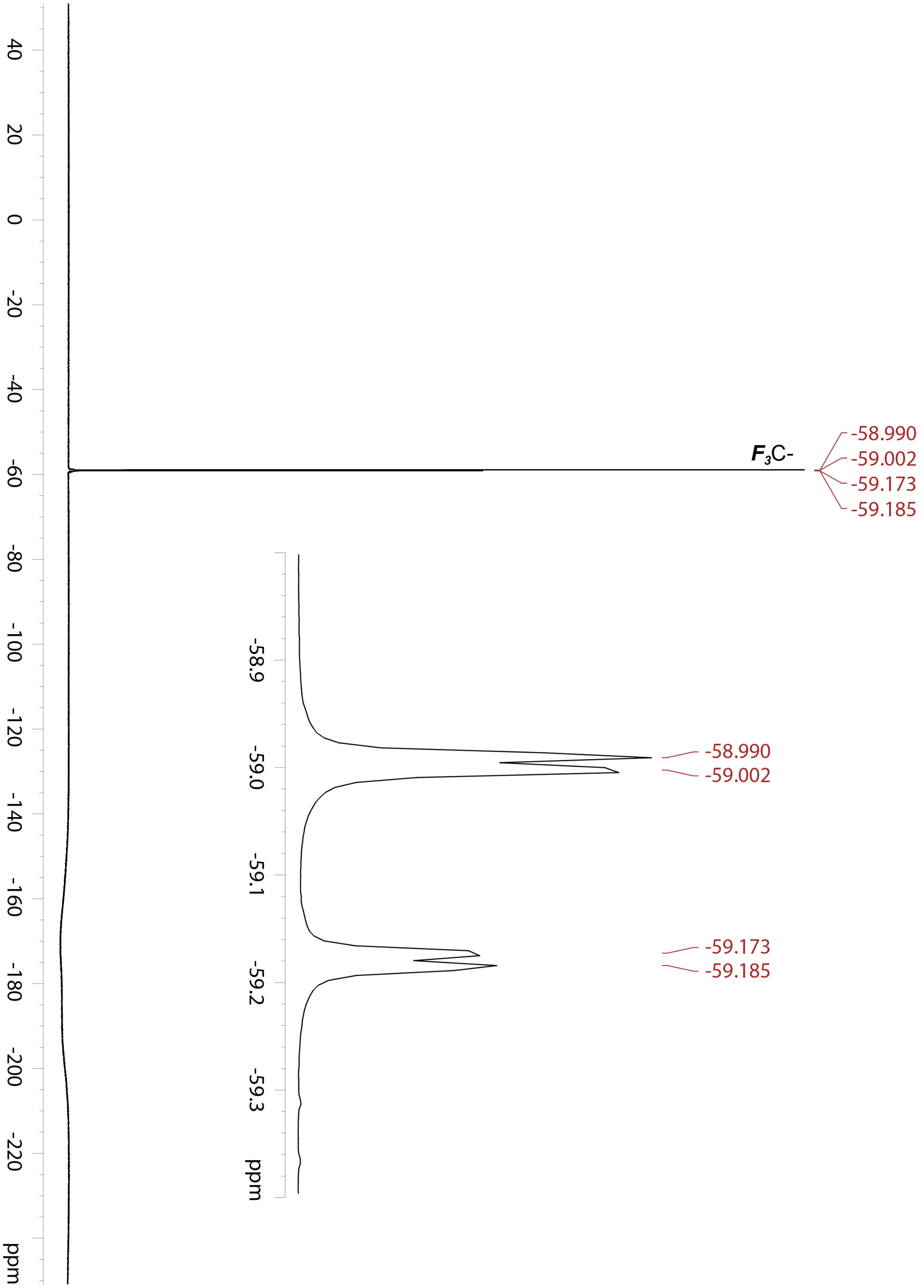

^{31}P-NMR (162 MHz, $CDCl_3$, 25 °C) of compound **A7**

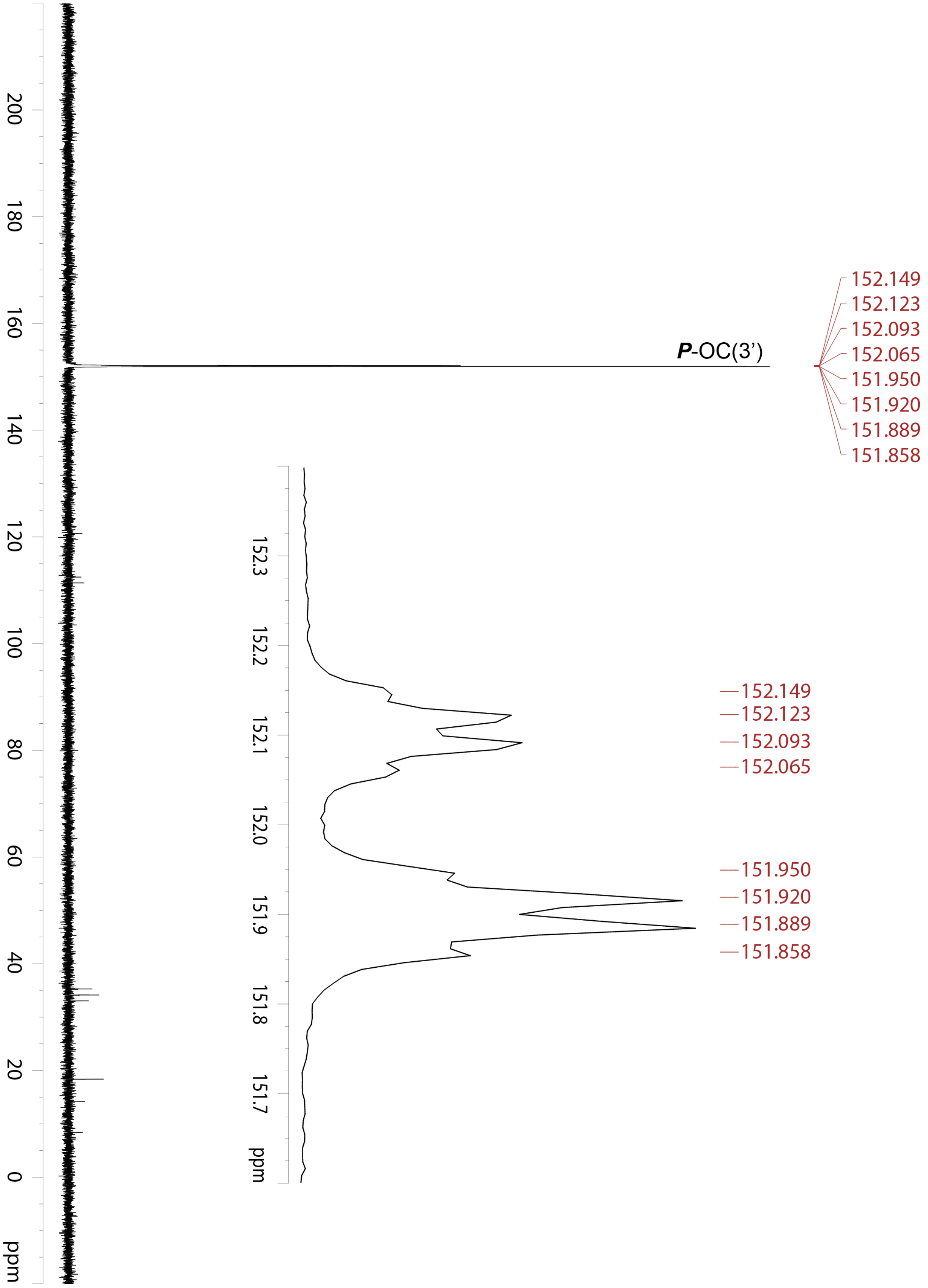

Synthesis of 2'-OCF$_3$ cytidine phosphoramidite C7a

3',5'-*O*-(1,1,3,3-Tetraisopropyldisiloxane-1,3-diyl)cytidine

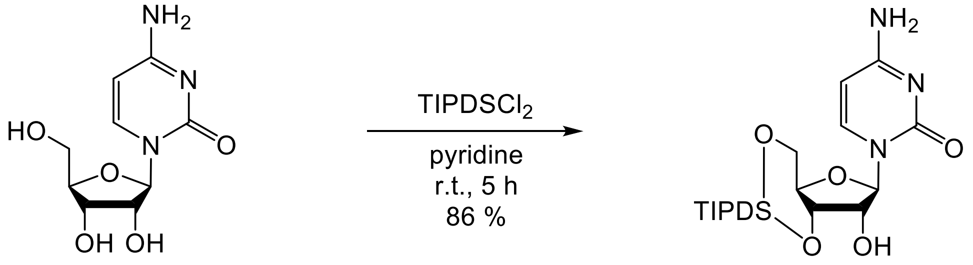

C1 **C2**

Cytidine (2.0 g, 8.22 mmol) was coevaporated two times with pyridine, suspended in dry pyridine (26.5 mL) and treated dropwise with 1,3-dichloro-1,1,3,3-tetraisopropyl disiloxane (TIPDSCl$_2$, 2.76 mL, 2.72 g, 8.63 mmol). After 5 hours, TLC showed complete conversion of starting material and the reaction was quenched with methanol (1 mL), evaporated under reduced pressure and coevaporated two times with toluene. The residue was dissolved in ethyl acetate, washed with citric acid solution (5 %), saturated sodium bicarbonate solution and brine, dried over sodium sulfate and concentrated. Crude product was purified by column chromatography on silica gel using 2 - 8 % methanol in dichloromethane as eluent. Yield: 3.46 g of compound **C2** (87 %) as white solid. TLC: (dichloromethane/methanol, 9/1) R_F = 0.36. ESI-MS: (m/z) $[M+H]^+$ calcd. 486.2450 found: 486.2432. ^{1}H-NMR (400 MHz, DMSO-*d$_6$*, 25 °C): δ 1.01 (28H, m, J=5.67 Hz, ***H$_3$***C-, -***H***C-(TIPDS)), 3.90 (2H, t, J=11.93 Hz, ***H(a)***-C(5')), 3.94 (1H, d, J=4.48 Hz, ***H***-C(2')), 4.00 (1H, d, J=9.08 Hz, ***H***-C(4')), 4.08 (1H, q, J=4.49 Hz, ***H***-C(3')), 4.15 (1H, d, J=13.20 Hz, ***H(b)***-C(5')), 5.55 (1H, s, ***H***O-C(2')), 5.60 (1H, d, J=4.52 Hz, ***H***-C(1')), 5.67 (1H, d, J=7.44 Hz, ***H***-C(5)), 7.14 (2H, d, J=23.69 Hz, ***H$_2$***N-C(4)), 7.71 (1H, d, J=7.44 Hz, ***H***-C(6)). ^{13}C-NMR (100 MHz, DMSO-*d$_6$*, 25 °C): δ 12.34 (4C, q, J=27.42 Hz, -***C***H-(TIPDS)), 17.05 (8C, m, J=9.38 Hz, ***C***H$_3$-(TIPDS)), 60.01 (1C, s, ***C***(5')), 68.44 (1C, s, ***C***(3')), 74.08 (1C, s, ***C***(2')), 80.45 (1C, s, ***C***(4')), 90.66 (1C, s, ***C***(1')), 93.18 (1C, s, ***C***(5)), 139.84 (1C, s, ***C***(6)), 154.75 (1C, s, ***C***(2)), 165.66 (1C, s, ***C***(4)).

^{1}H-NMR (400 MHz, DMSO-d_6, 25 °C) of compound **C2**

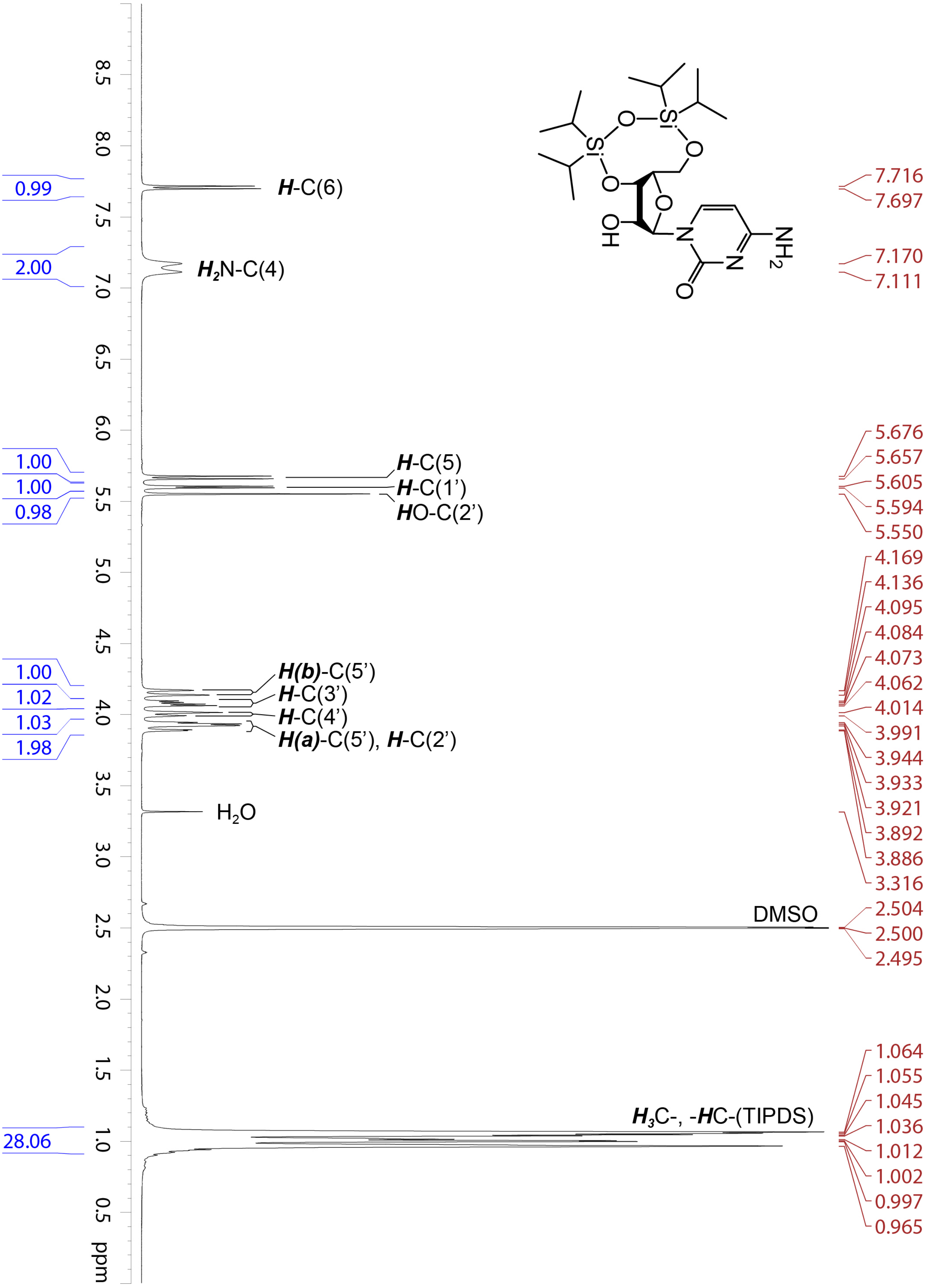

^{13}C-NMR (100 MHz, DMSO-d_6, 25 °C) of compound **C2**

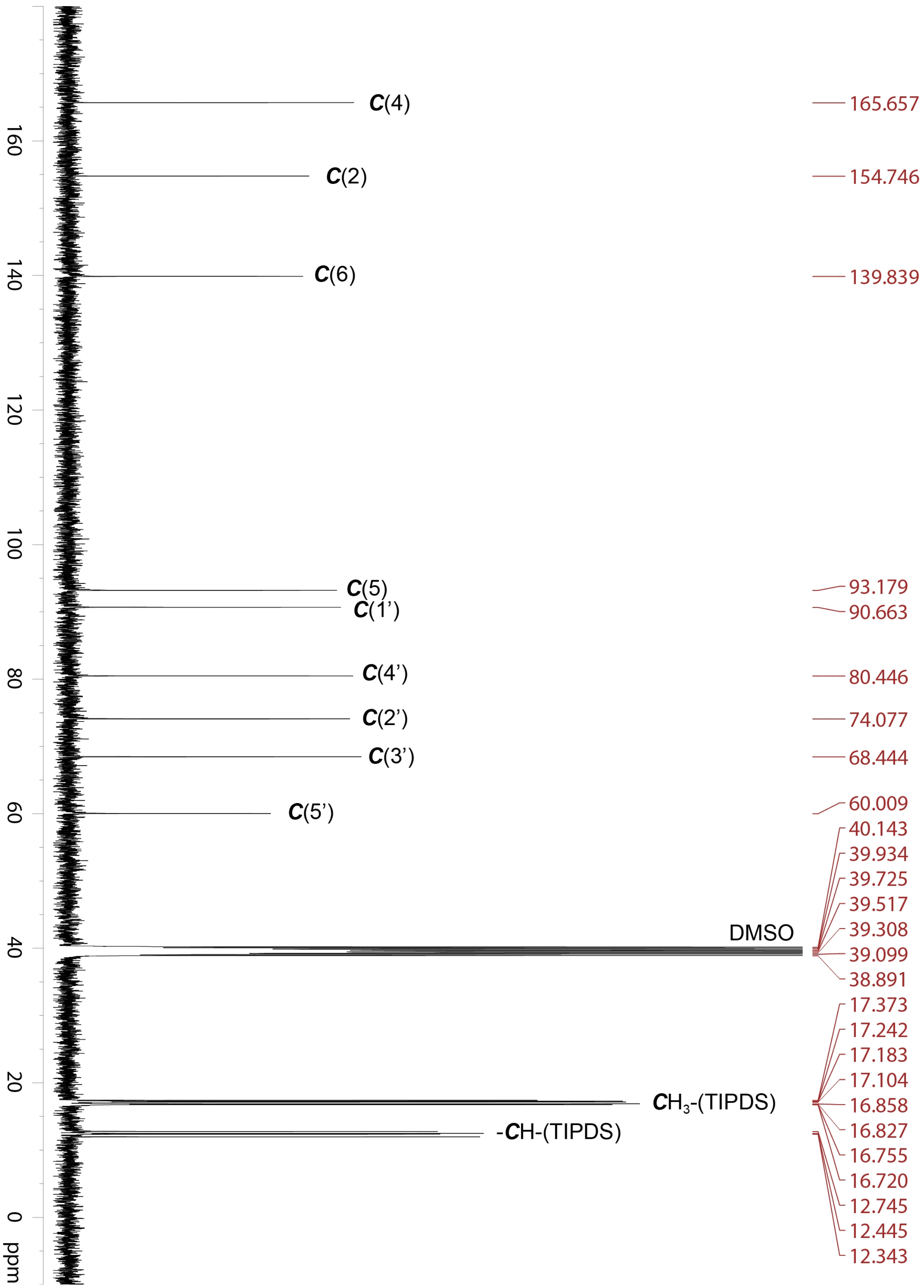

2'-*O*-[(Methylthio)thiocarbonyl]-3',5'-*O*-(1,1,3,3-tetraisopropyldisiloxane-1,3-diyl)cytidine

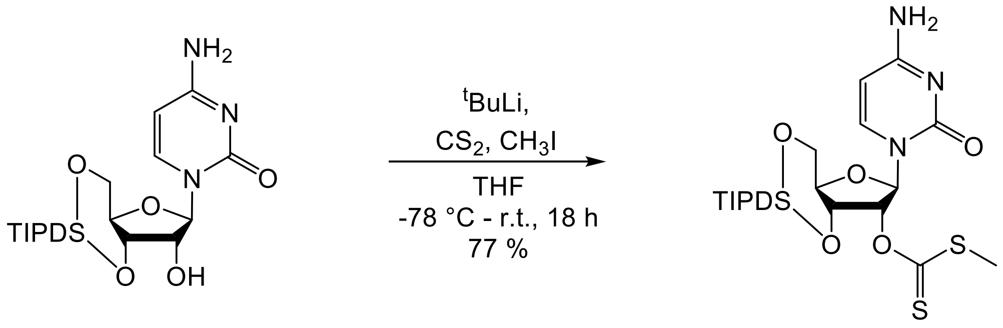

C2 **C3**

Compound **C2** (3.45 g, 7.10 mmol) was suspended in anhydrous THF (57 mL), cooled to -78 °C and treated with *tert*-butyllithium in pentane solution (1.7 M, 5.2 mL, 566 mg, 8.88 mmol). After ten minutes carbon disulfide (3.84 mL, 4.87 g, 63.9 mmol) was added and after stirring for one hour at -78 °C methyl iodide (1.33 mL, 3.02 g, 21.3 mmol) was added. The solution was allowed to warm to room temperatures overnight and upon completion, dichloromethane (200 mL) was added. Organic phases were washed with saturated sodium bicarbonate solution and brine, dried over sodium sulfate and evaporated. Crude product was purified by column chromatography on silica gel, eluting with 2 - 6 % methanol in dichloromethane. Yield: 3.13 g of compound **C3** (77 %) as slight yellow solid. TLC: (dichloromethane/methanol, 9/1): R_F = 0.41. ESI-MS: (m/z) $[M+H]^+$ calcd. 576.2048 found: 576.2023. ^{1}H-NMR (400 MHz, DMSO-d_6, 25 °C) δ 1.01 (28H, m, J=5.14 Hz, ***H*₃**C-, -***H***C-(TIPDS), 2.59 (3H, s, $\boldsymbol{H_3}$C-S), 3.91 (1H, m, J=3.11 Hz, ***H***-C(4')), 3.97 (1H, dd, J=2.82, 12.66 Hz, ***H(a)***-C(5')), 4.08 (1H, dd, J=4.44, 12.68 Hz, ***H(b)***-C(5')), 4.79 (1H, dd, J=5.68, 8.40 Hz, ***H***-C(3')), 5.71 (2H, d, J=7.36 Hz, ***H***-C(1'), ***H***-C(5)), 6.28 (1H, dd, J=1.22, 5.62 Hz, ***H***-C(2')), 7.28 (2H, d, J=18.45 Hz, $\boldsymbol{H_2}$N-C(4)), 7.64 (1H, d, J=7.44 Hz, ***H***-C(6)). ^{13}C-NMR (100 MHz, DMSO-d_6, 25 °C) δ 12.38 (4C, q, J=16.18 Hz, -***C***H-(TIPDS)), 16.99 (8C, m, J=10.08 Hz, ***C***H_3-(TIPDS)), 18.40 (1C, s, ***C***H_3-S), 61.24 (1C, s, ***C***(5')), 69.90 (1C, s, ***C***(3')), 81.50 (1C, s, ***C***(4')), 83.06 (1C, s, ***C***(2')), 90.14 (1C, s, ***C***(1')), 94.19 (1C, s, ***C***(5)), 142.46 (1C, s, ***C***(6)), 154.44 (1C, s, ***C***(2)), 165.94 (1C, s, ***C***(4)), 214.60 (1C, s, -***C***S_2-).

^{1}H-NMR (400 MHz, DMSO-d_6, 25 °C) of compound **C3**

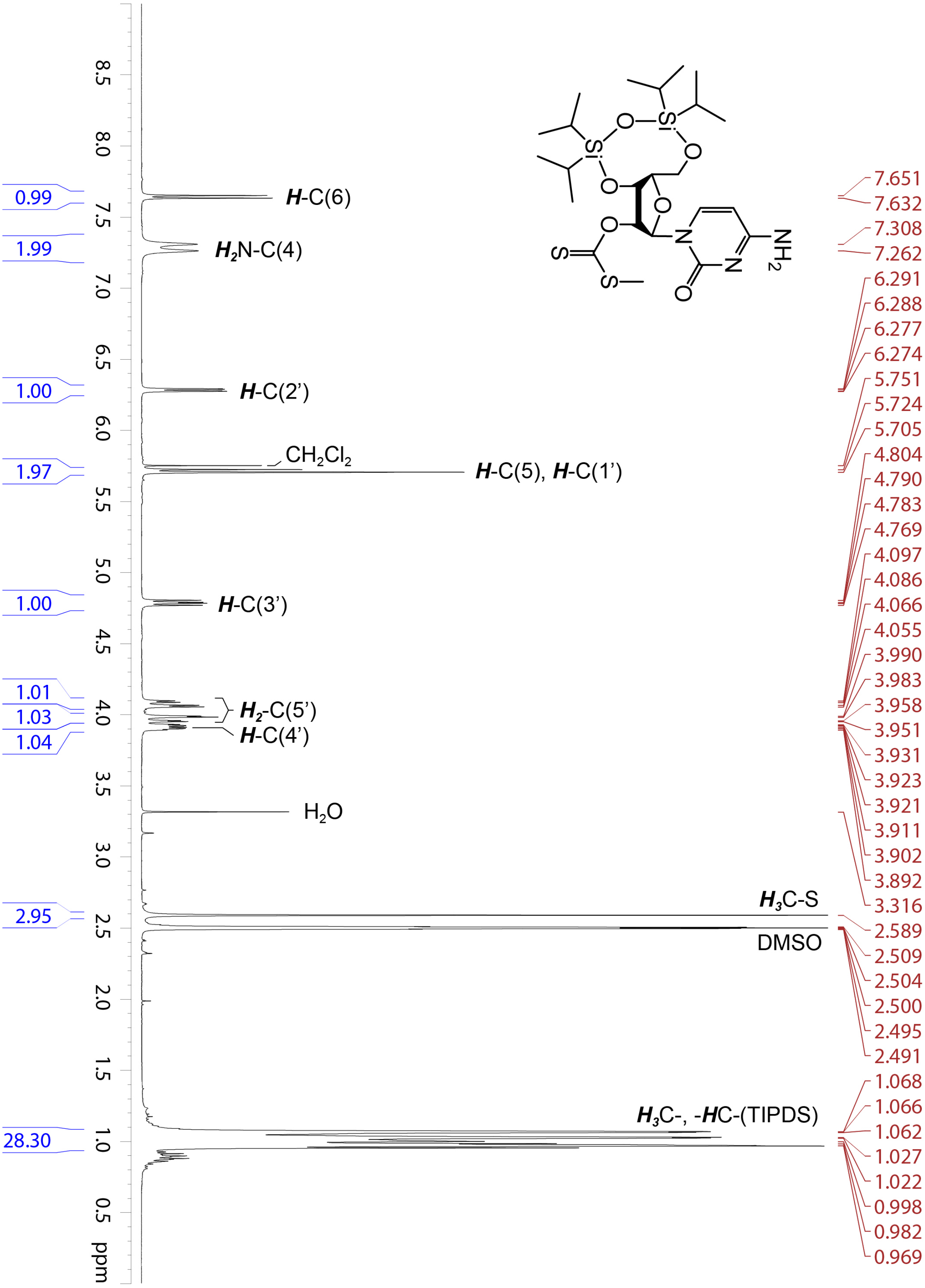

^{13}C-NMR (100 MHz, DMSO-d_6, 25 °C) of compound **C3**

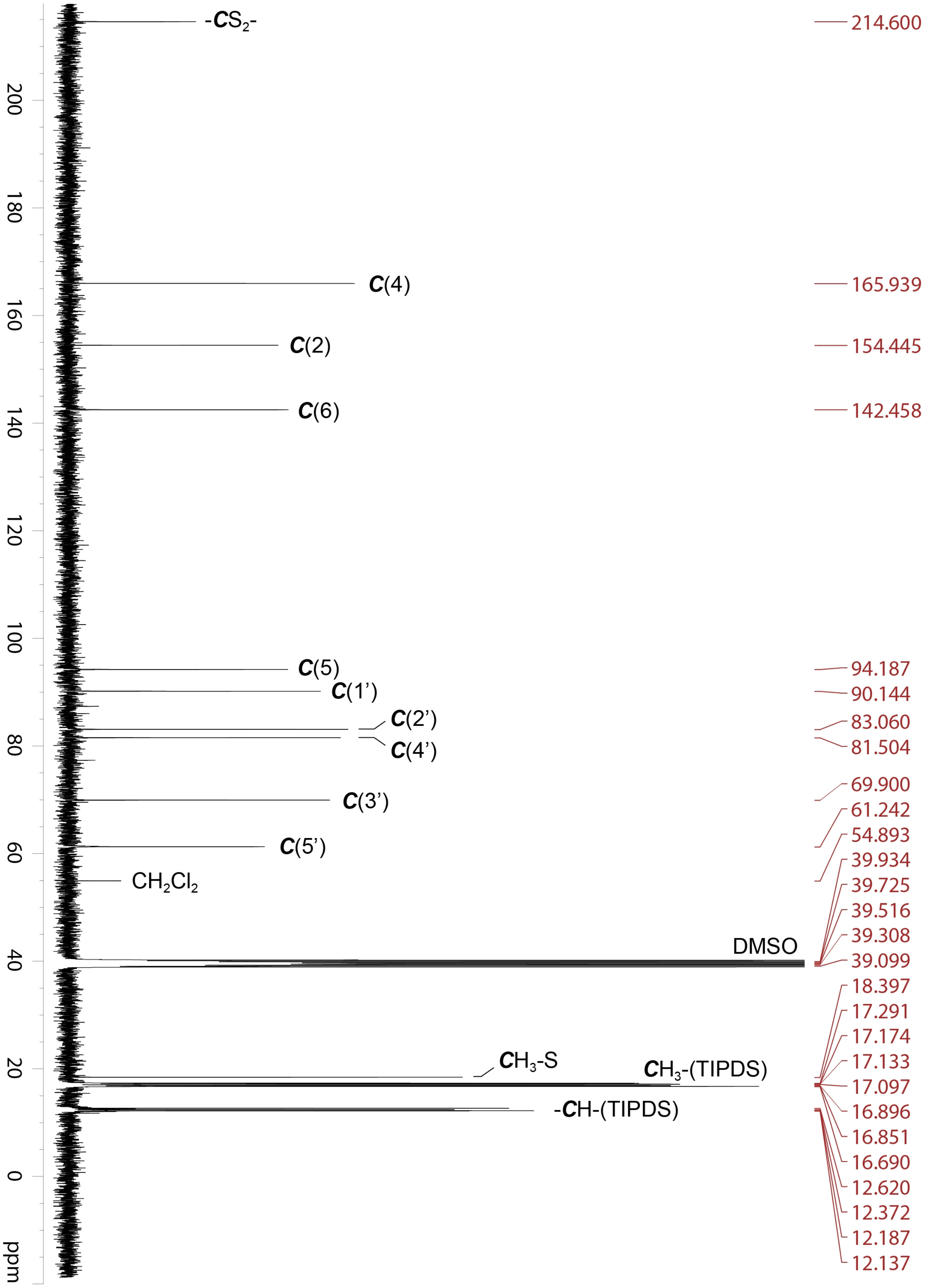

***N*[4]-Acetyl-2'-*O*-[(methylthio)thiocarbonyl]-3',5'-*O*-(1,1,3,3-tetraisopropyldisiloxane-1,3-diyl)cytidine**

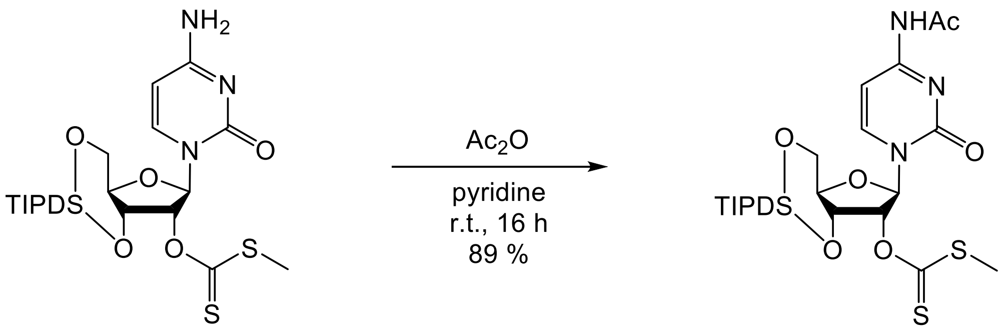

C3 **C4a**

Compound **C3** (1.23 g, 1.14 mmol) was dissolved in dry pyridine (8.6 mL), treated with acetic anhydride (Ac_2O, 606 µL, 654 mg, 6.41 mmol) and stirred for 16 hours at ambient temperatures. Upon completion, solvents were evaporated and residue was coevaporated twice with toluene. Crude product was purified by column chromatography on silica gel, eluting with 1 – 4 % methanol in dichloromethane. Yield: 1.17 g of compound **C4a** (89 %) as white foam. TLC: (dichloromethane/methanol, 9/1): R_F = 0.72. ESI-MS: (m/z) $[M+H]^+$ calcd. 618.2154 found: 618.2177. ^{1}H-NMR (400 MHz, $CDCl_3$, 25 °C) δ 1.03 (28H, m, J=9.15 Hz, ***H*3**C-, -***H***C-(TIPDS)), 2.24 (3H, s, ***H*3**C-(Ac)), 2.58 (3H, s, ***H*3**C-S), 4.02 (1H, dd, J=2.60, 13.40 Hz, ***H(a)***-C(5')), 4.14 (1H, d, J=9.28 Hz, ***H***-C(4')), 4.27 (1H, d, J=13.24 Hz, ***H(b)***-C(5')), 4.48 (1H, q, J=4.65 Hz, ***H***-C(3')), 5.93 (1H, s, ***H***-C(1')), 6.35 (1H, d, J=4.68 Hz, ***H***-C(2')), 7.43 (1H, d, J=7.52 Hz, ***H***-C(5)), 8.14 (1H, d, J=7.56 Hz, ***H***-C(6)), 9.50 (1H, b, ***H***N-C(4)). ^{13}C-NMR (100 MHz, $CDCl_3$, 25 °C) δ 13.14 (4C, q, J=21.36 Hz, -***C***H-(TIPDS)), 17.25 (8C, m, J=10.14 Hz, ***C***H_3-(TIPDS)), 19.38 (1C, s, ***C***H_3-S), 25.12 (1C, s, ***C***H_3-(Ac)), 59.83 (1C, s, ***C***(5')), 68.50 (1C, s, ***C***(3')), 82.68 (2C, d, J=20.19 Hz, ***C***(2'), ***C***(4')), 89.80 (1C, s, ***C***(1')), 96.71 (1C, s, ***C***(5)), 144.42 (1C, s, ***C***(6)), 154.65 (1C, s, ***C***(2)), 163.15 (1C, s, ***C***(4)), 170.82 (1C, s, -***C***O-(Ac)), 214.69 (1C, s, -***C***S_2-).

^{1}H-NMR (400 MHz, $CDCl_3$, 25 °C) of compound **C4a**

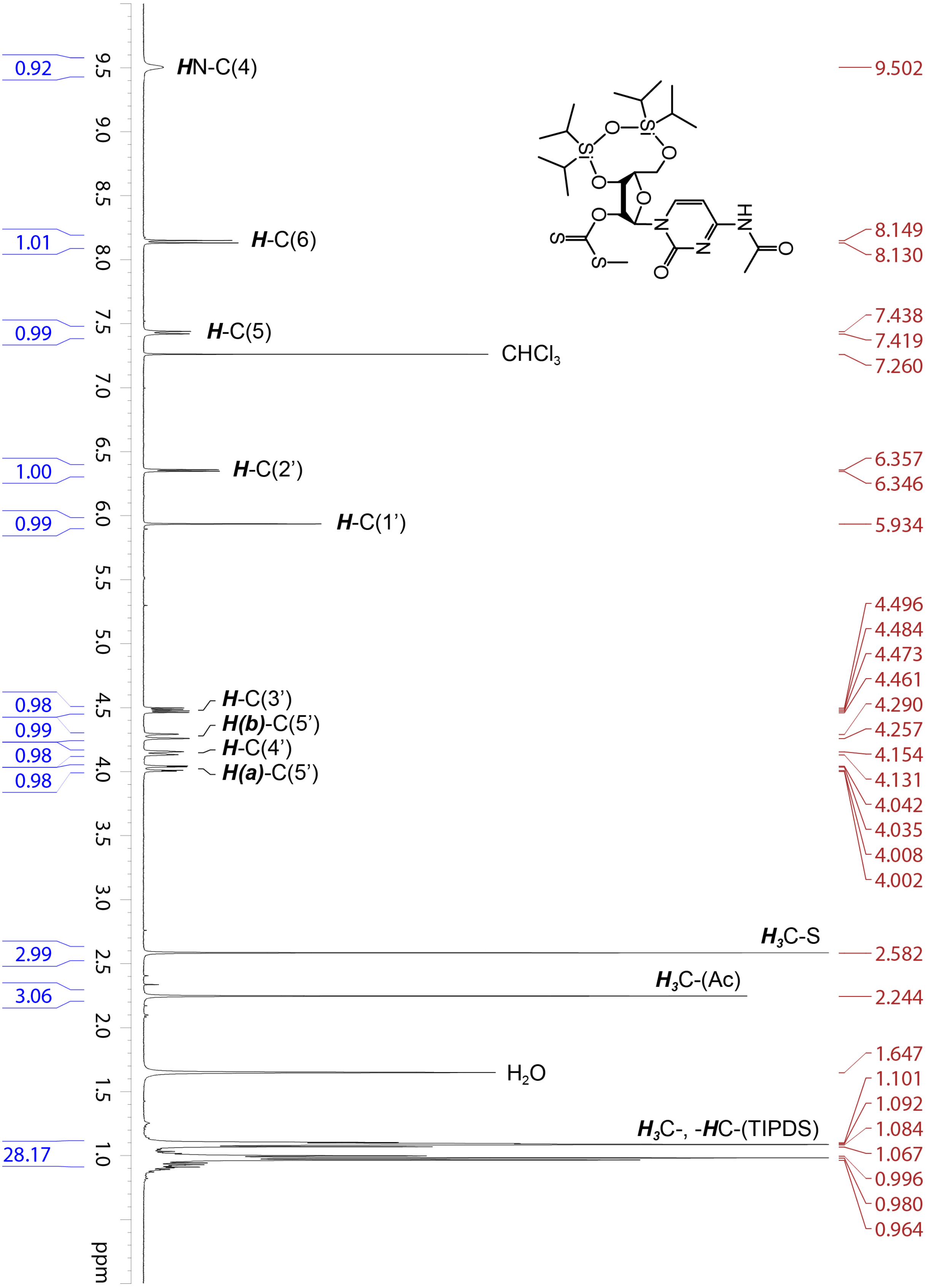

^{13}C-NMR (100 MHz, $CDCl_3$, 25 °C) of compound **C4a**

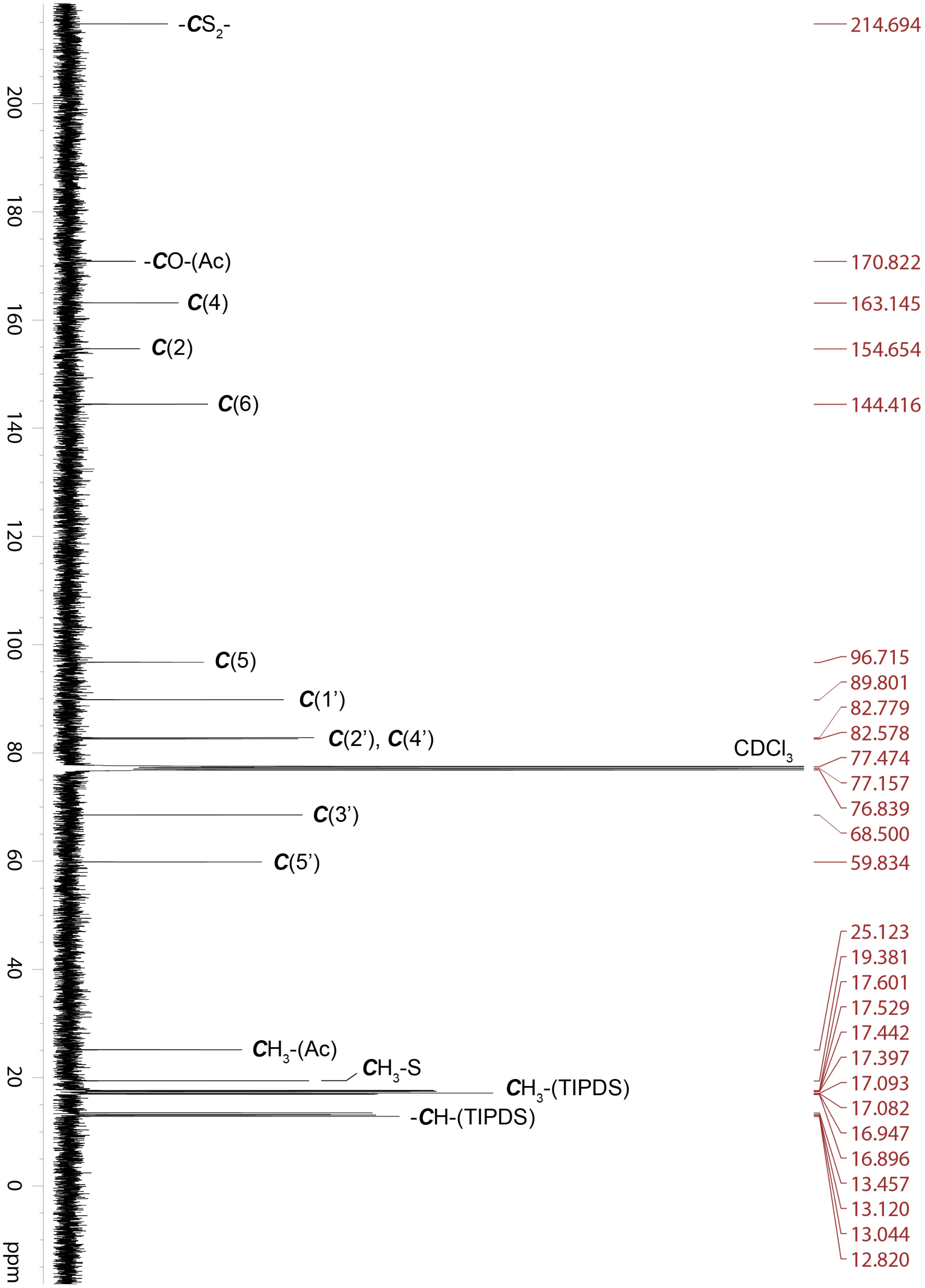

***N*[4]-Acetyl-2'-*O*-(trifluoromethyl)cytidine**

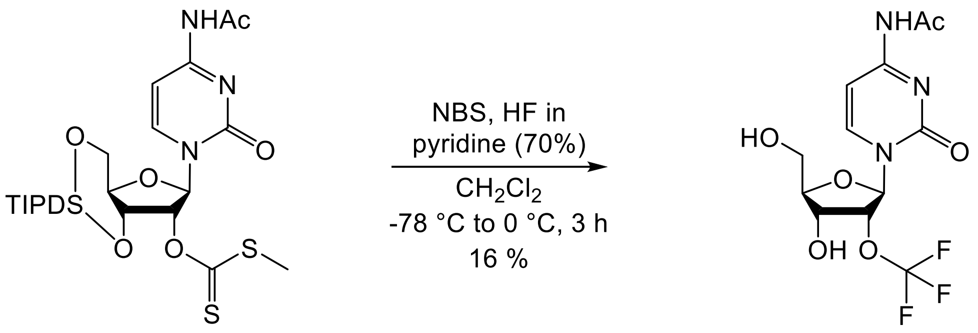

C4a **C5a**

To a suspension of *N*-bromosuccinimide (NBS, 2.0 g, 11.3 mmol) in dichloromethane (14.5 mL), hydrogen fluoride in pyridine complex (70 % HF, 14.6 g, 13.3 mL) was added at -78 °C, and subsequently, a solution of compound **C4a** (1.39 g, 2.25 mmol) in dichloromethane (14.5 mL) was added to the mixture. The reaction mixture was allowed to warm to 0 °C and stirred for three hours. Upon completion, the reaction mixture was poured carefully into a flask containing saturated sodium bicarbonate solution (250 mL) and sodium thiosulfate solution (10 %, 180 mL) while vigorously stirring. Further bicarbonate solution was added until neutral pH was obtained. The aqueous layer was extracted several times with chloroform/isopropanol (3/1). Organic phases were combined, dried over sodium sulfate and evaporated. Crude product was purified by column chromatography on silica gel, using 2 - 6 % methanol in dichloromethane as eluent. Yield: 91.0 mg of compound **C5a** (11 %) as white foam. TLC: (dichloromethane/methanol, 9/1): R_F = 0.31. ESI-MS: (m/z) $[M+H]^+$ calcd. 354.0907 found: 354.0881. ^{1}H-NMR (400 MHz, DMSO-*d*$_6$, 25 °C) δ 2.10 (3H, s, ***H*$_3$**C-(Ac)), 3.70 (2H, qdd, J=2.63, 12.36, 62.49 Hz, ***H*$_2$**-C(5')), 3.96 (1H, t, J=3.07 Hz, ***H***-C(4')), 4.23 (1H, q, J=5.65 Hz, ***H***-C(3')), 4.89 (1H, t, J=4.15 Hz, ***H***-C(2')), 5.30 (1H, t, J=4.93 Hz, ***H***O-C(5')), 5.78 (1H, d, J=6.05 Hz, ***H***O-C(3')), 6.03 (1H, d, J=3.45 Hz, ***H***-C(1')), 7.21 (1H, d, J=7.51 Hz, ***H***-C(5)), 8.41 (1H, d, J=7.55 Hz, ***H***-C(6)), 10.95 (1H, s, ***H***N-C(4)). ^{13}C-NMR (100 MHz, DMSO-*d*$_6$, 25 °C) δ 24.37 (1C, s, ***C***H_3-(Ac)), 59.40 (1C, s, ***C***(5')), 67.15 (1C, s, ***C***(3')), 80.30 (1C, s, ***C***(2')), 84.26 (1C, s, ***C***(4')), 87.30 (1C, s, ***C***(1')), 95.66 (1C, s, ***C***(5)), 145.09 (1C, s, ***C***(6)), 155.10 (1C, s, ***C***(2)), 162.73 (1C, s, ***C***(4)), 171.10 (1C, s, -***C***O-(Ac)). ^{19}F-NMR (376 MHz, DMSO-*d*$_6$, 25 °C) δ -56.76 (3F, s, ***F*$_3$**C-).

^{1}H-NMR (400 MHz, DMSO-d_6, 25 °C) of compound **C5a**

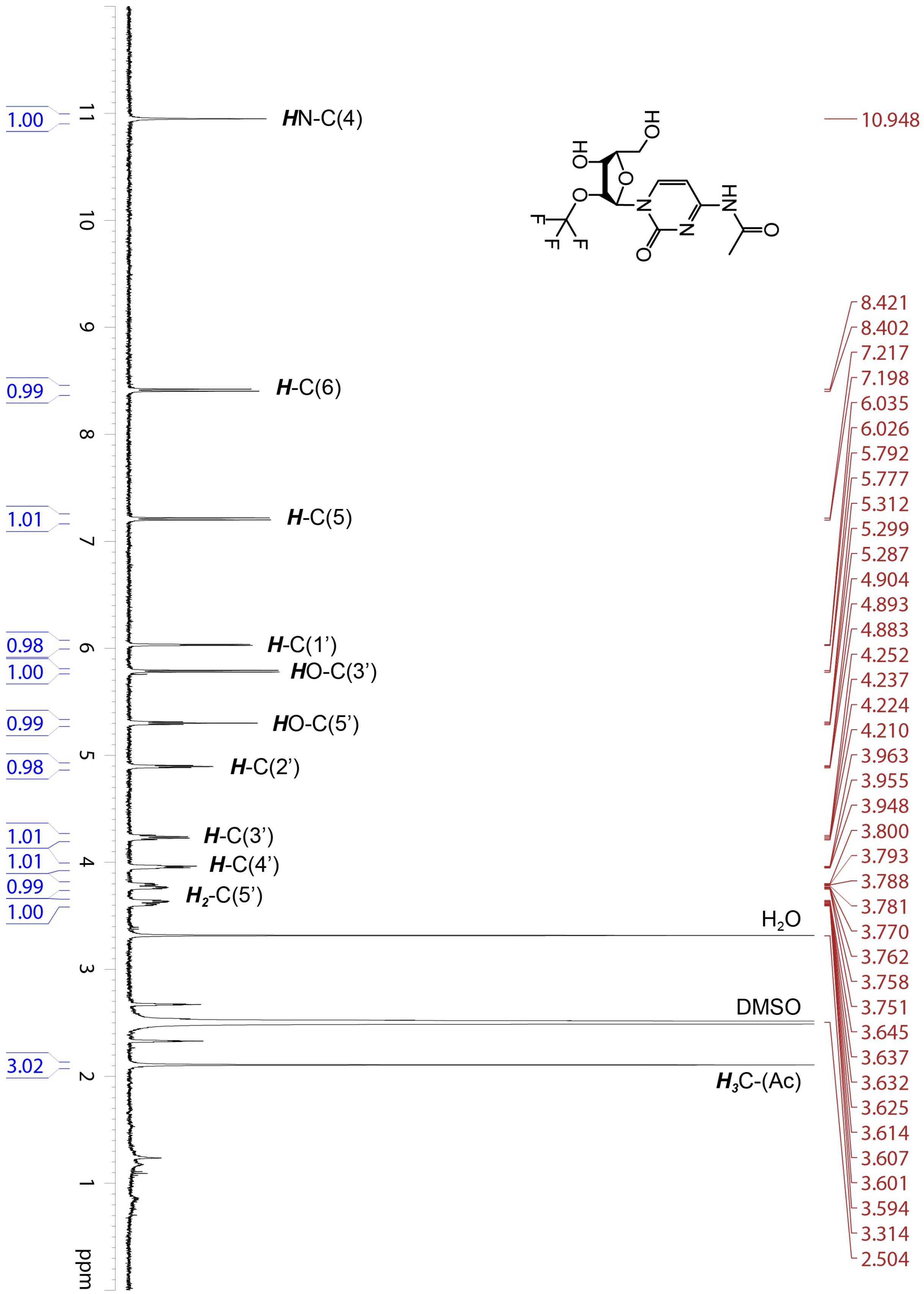

^{13}C-NMR (100 MHz, DMSO-d_6, 25 °C) of compound **C5a**

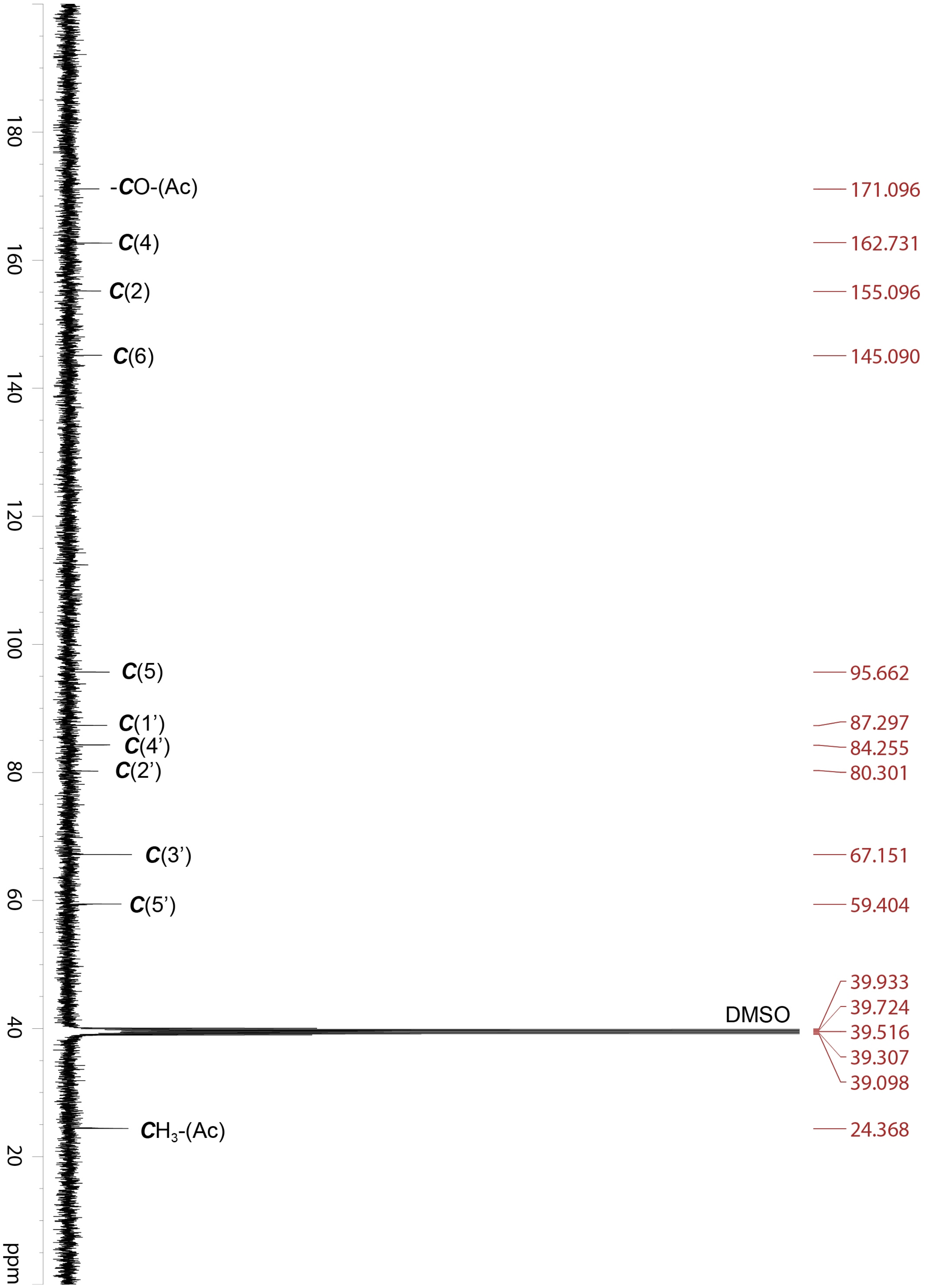

^{19}F-NMR (376 MHz, DMSO-d_6, 25 °C) of compound **C5a**

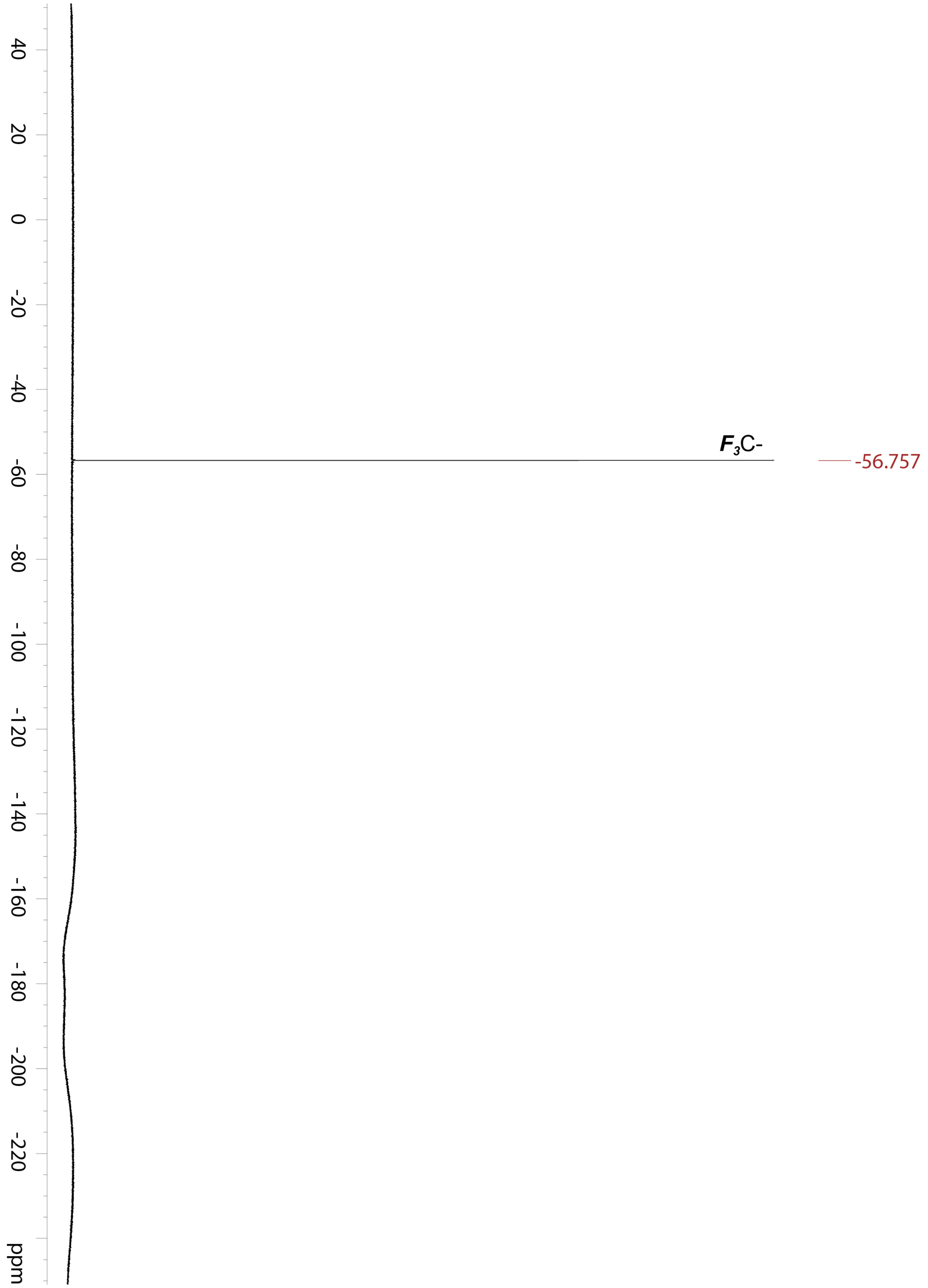

N^4-Acetyl-5'-O-(4,4-dimethoxytrityl)-2'-O-(trifluoromethyl)cytidine

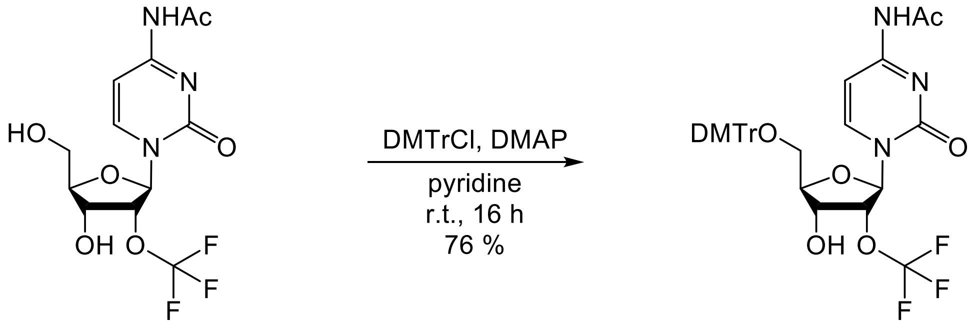

C5a **C6a**

Compound **C5a** (81 mg, 0.23 mmol) was dissolved in dry pyridine (1.4 mL) under argon atmosphere and treated with 4,4'-dimethoxytrityl chloride (DMTrCl, 109 mg, 0.32 mmol) and 4-dimethylaminopyridine (DMAP, 11.2 mg, 0.09 mmol) in four portions over a period of 1 hour at ambient temperatures. The reaction mixture was stirred overnight, quenched with methanol (0.2 mL) and diluted with diethyl ether. Organic phases were washed twice with water, thrice with citric acid solution (5 %) and twice with saturated sodium bicarbonate solution. Combined phases were dried over sodium sulfate, concentrated and coevaporated with toluene two times. Crude product was purified by column chromatography on silica gel, eluting with 1 – 4 % methanol in dichloromethane + 2 % triethylamine. Yield: 114 mg of compound **C6a** (76 %) as white foam. TLC: (dichloromethane/methanol, 19/1): R_F = 0.56. ESI-MS: (m/z) $[M+H]^+$ calcd. 656.2214 found: 656.2196. ^{1}H-NMR (400 MHz, $CDCl_3$, 25 °C) δ 2.22 (3H, s, ***H*₃**C-(Ac)), 3.62 (2H, m, J=5.47 Hz, $\boldsymbol{H_2}$-C(5')), 3.82 (6H, d, J=1.90 Hz, $\boldsymbol{H_3}$C-O(DMTr)), 4.29 (1H, d, J=7.66 Hz, ***H***-C(4')), 4.65 (1H, q, J=4.03 Hz, ***H***-C(3')), 5.12 (1H, q, J=2.08 Hz, ***H***-C(2')), 6.17 (1H, d, J=1.90 Hz, ***H***-C(1')), 6.88 (4H, d, J=8.95 Hz, ***H***-C(DMTr)), 7.18 (1H, d, J=7.56 Hz, ***H***-C(5)), 7.33 (7H, m, J=3.17 Hz, ***H***-C(DMTr)), 7.44 (2H, t, J=4.29 Hz, ***H***-C(DMTr)), 8.39 (1H, d, J=7.58 Hz, ***H***-C(6)), 9.92 (1H, b, ***H***N-C(4)). ^{13}C-NMR (100 MHz, $CDCl_3$, 25 °C) δ 24.79 (1C, s, ***C***H_3-(Ac)), 55.28 (2C, s, ***C***H_3-O(DMTr)), 61.33 (1C, s, ***C***(5')), 67.96 (1C, s, ***C***(3')), 80.11 (1C, s, ***C***(2')), 82.49 (1C, s, ***C***(4')), 87.30 (1C, s, ***C***(DMTr)), 88.58 (1C, s, ***C***(1')), 97.34 (1C, s, ***C***(5)), 113.43 (4C, s, ***C***(DMTr)), 122.88 (1C, s, -***C***F_3), 127.28 (1C, s, ***C***(DMTr)), 128.18 (4C, d, J=6.71 Hz, ***C***(DMTr)), 130.14 (4C, d, J=2.24 Hz, ***C***(DMTr)), 135.29 (2C, d, J=27.17 Hz, ***C***(DMTr)), 144.42 (2C, d, J=48.78 Hz, ***C***(6), ***C***(DMTr)), 155.48 (1C, s, ***C***(2)), 158.79 (2C, d, J=1.08 Hz, ***C***(DMTr)), 163.17 (1C, s, ***C***(4)), 171.00 (1C, s, -***C***O-(Ac)). ^{19}F-NMR (376 MHz, $CDCl_3$, 25 °C) δ -58.17 (3F, s, $\boldsymbol{F_3}$C-).

^{1}H-NMR (400 MHz, $CDCl_3$, 25 °C) of compound **C6a**

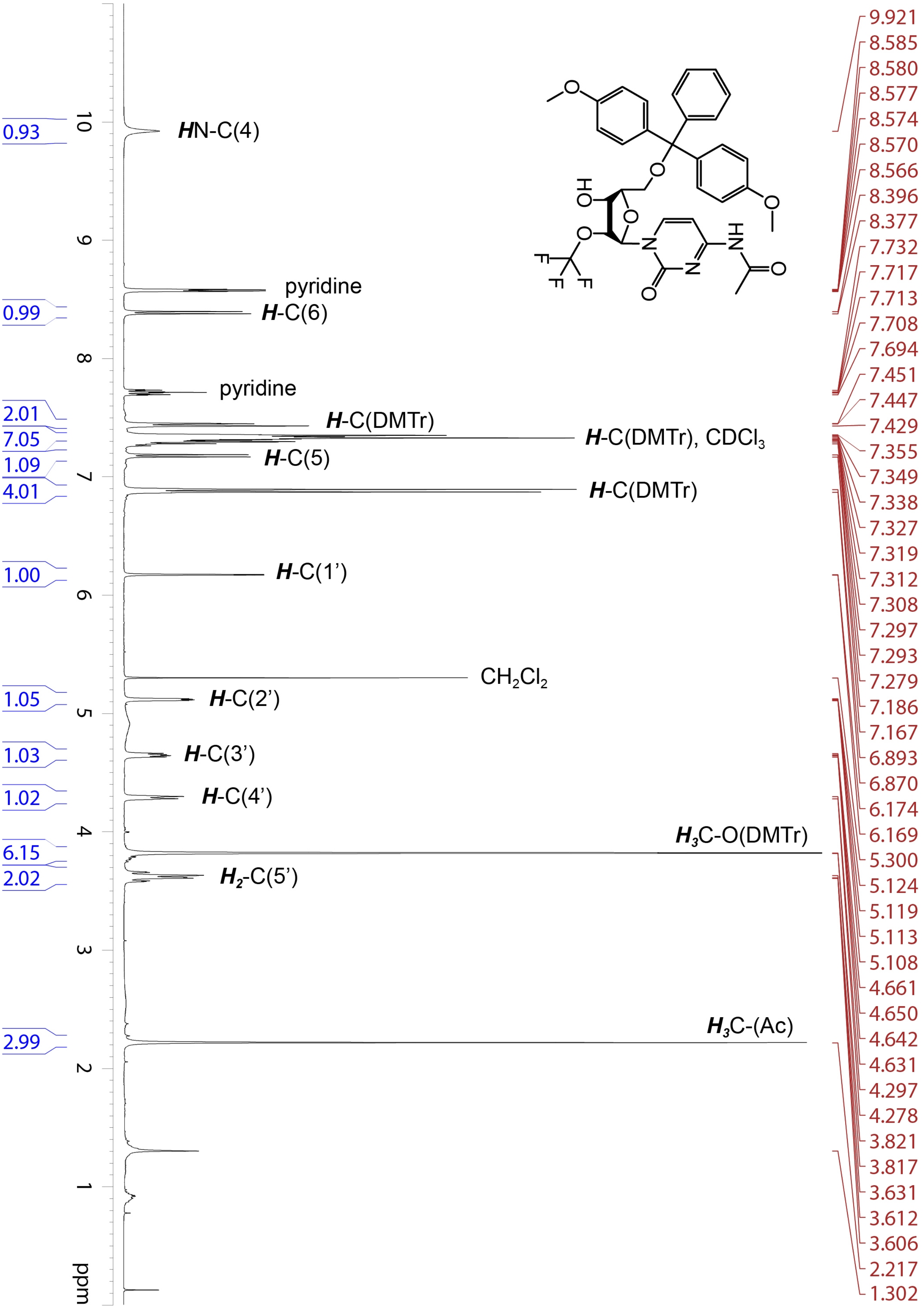

^{13}C-NMR (100 MHz, $CDCl_3$, 25 °C) of compound **C6a**

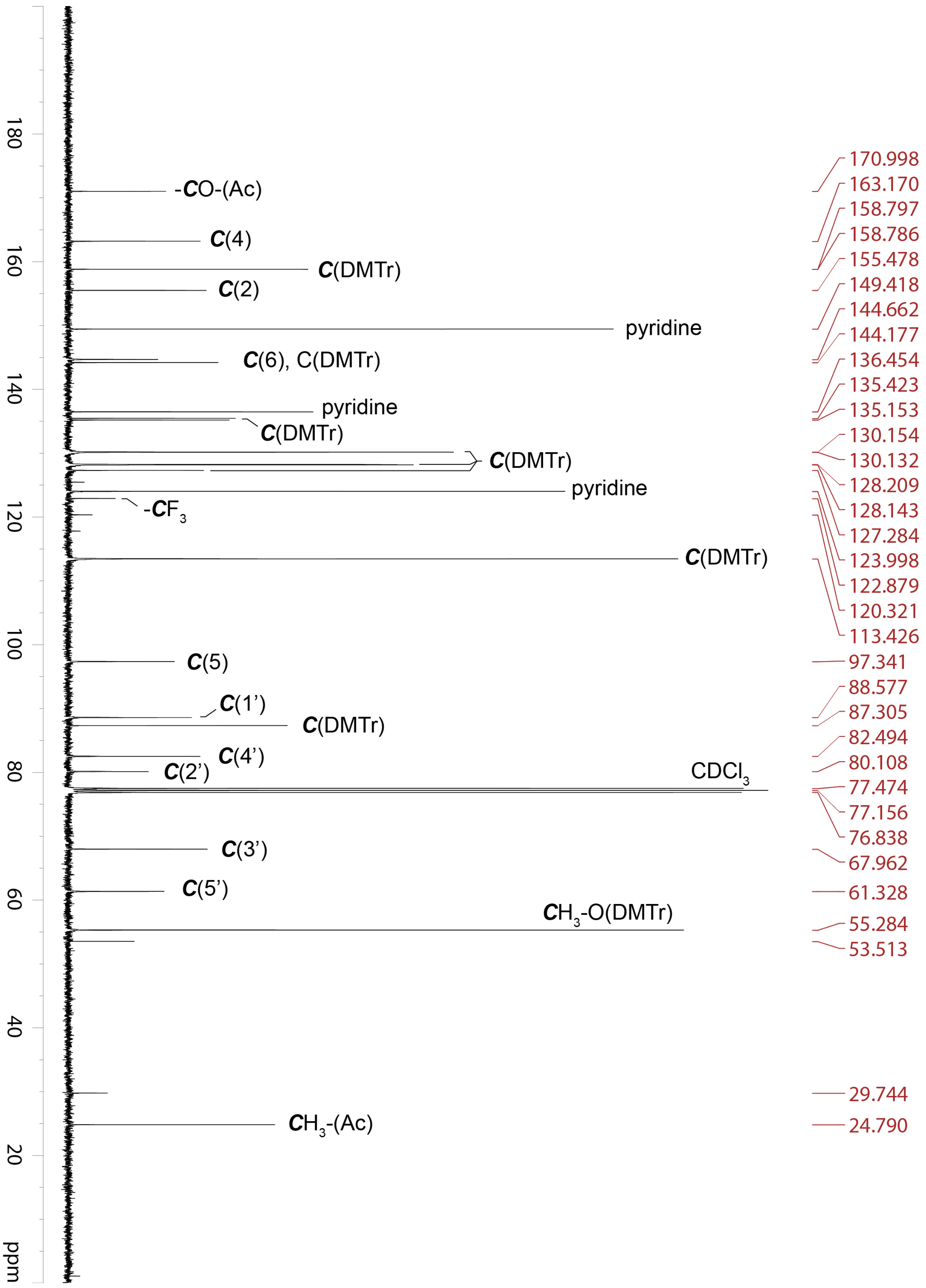

^{19}F-NMR (376 MHz, $CDCl_3$, 25 °C) of compound **C6a**

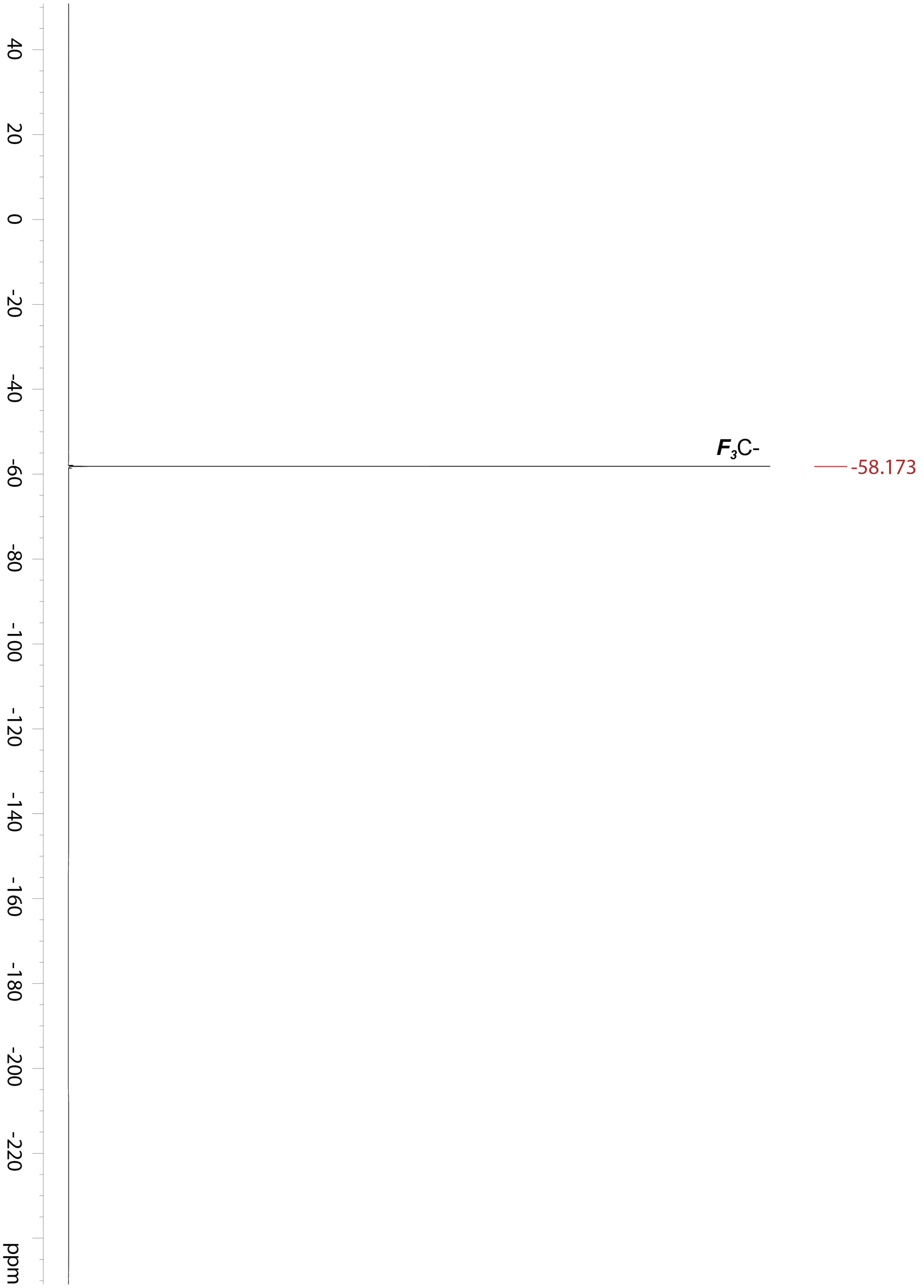

N^4-Acetyl-2'-*O*-trifluoromethyl-5'-*O*-(4,4-dimethoxytrityl)cytidine 3'-(2-cyanoethyl)-*N*,*N*-diisopropylphosphoramidite

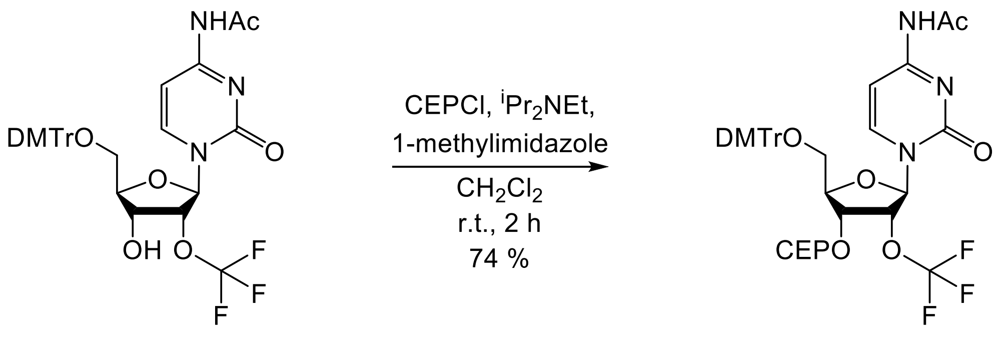

C6a **C7a**

Compound **C6a** (105 mg, 0.16 mmol) was dissolved in anhydrous dichloromethane (1.0 mL) and treated with diisopropylethylamine (0.19 mL, 138 mg, 1.20 mmol), 2-cyanoethyl-*N,N*-diisopropylchloro phosphoramidite (CEPCl, 89.4 µL, 94.8 mg, 0.40 mmol) and 1-methyl imidazole (6.4 µL, 6.57 mg, 0.08 mmol). The reaction mixture was stirred for 2 hours at ambient temperatures and TLC showed complete conversion of starting material. The reaction was quenched with methanol (0.2 mL) and all volatiles were evaporated. Crude product was purified by column chromatography on silica gel, using 25 - 75 % ethyl acetate in hexanes + 2 % triethylamine as eluent. Yield: 101 mg of a 3:2 mixture of diastereomers of compound **C7a** (74 %) as white foam. TLC: (ethyl acetate/hexanes, 2/1): R_F = 0.23, 0.29, 0.46. ESI-MS: (m/z) $[M+H]^+$ calcd. 856.3293 found: 856.3272. ^{1}H-NMR (400 MHz, $CDCl_3$, 25 °C) δ 1.13 (12H, m, J=13.91 Hz, ***H*₃**C-CNP), 2.27 (3H, d, J=2.28 Hz, $\boldsymbol{H_3}$C-(Ac)), 2.42 (2H, m, J=3.05 Hz, ***H(a)***C-COP), 2.61 (1H, t, J=6.16 Hz, ***H(b)***C-COP), 3.50 (1H, q, J=4.55 Hz, ***H(a)***-C(5')), 3.60 (2H, m, J=5.20 Hz, -***H***C-NP), 3.68 (1H, d, J=9.12 Hz, ***H(b)***-C(5')), 3.74 (1H, m, J=4.76 Hz, ***H(a)***C-OP), 3.83 (6H, d, J=2.64 Hz, $\boldsymbol{H_3}$C-O(DMTr)), 3.89 (1H, m, J=3.50 Hz, ***H(b)***C-OP), 4.30 (1H, q, J=11.39 Hz, ***H***-C(4')), 4.64 (1H, q, J=3.09 Hz, ***H***-C(3')), 4.97 (1H, q, J=4.01 Hz, ***H***-C(2')), 6.22 (1H, d, J=2.72 Hz, ***H***-C(1')), 6.88 (4H, q, J=4.25 Hz, ***H***-C(DMTr)), 7.09 (1H, q, J=6.90 Hz, ***H***-C(5)), 7.32 (7H, m, J=4.35 Hz, ***H***-C(DMTr)), 7.41 (2H, t, J=3.48 Hz, ***H***-C(DMTr)), 8.28 (1H, q, J=3.87 Hz, ***H***-C(6)), 10.39 (1H, d, J=19.41 Hz, ***H***N-C(4)). ^{13}C-NMR (100 MHz, $CDCl_3$, 25 °C) δ 20.36 (1C, q, J=6.75 Hz; -***C***H_2-COP), 24.66 (5C, m, J=8.67 Hz, ***C***H_3-CNP, ***C***H_3-(Ac)), 43.37 (2C, t, J=11.24 Hz, -***C***H-NP), 55.35 (2C, d, J=3.94 Hz, ***C***H_3-O(DMTr)), 58.22 (1C, q, J=16.94 Hz, -***C***H_2-OP), 61.14 (1C, d, J=13.47 Hz, ***C***(5')), 69.49 (1C, q, J=18.09 Hz, ***C***(3')), 78.81 (1C, d, J=104.40 Hz, ***C***(2')), 82.48 (1C, t, J=20.86 Hz, ***C***(4')), 87.40 (1C, s, ***C***(DMTr)), 88.33 (1C, d, J=16.53 Hz, ***C***(1')), 97.46 (1C, s, ***C***(5)), 113.45 (4C, s, ***C***(DMTr)), 117.55 (1C, d, J=12.18 Hz, -***C***N), 122.88 (1C, s, -***C***F_3), 127.40 (1C, d, J=4.35 Hz, ***C***(DMTr)), 128.28 (4C, t, J=9.16 Hz, ***C***(DMTr)), 130.29 (4C, d, J=4.84 Hz, ***C***(DMTr)), 135.09 (2C, q, J=11.28 Hz, ***C***(DMTr)), 144.07 (2C, d, J=12.32 Hz, ***C***(6), ***C***(DMTr)), 155.03 (1C, s, ***C***(2)), 158.91 (2C, d, J=1.79 Hz, ***C***(DMTr)), 163.52 (1C, d, J=4.28 Hz, ***C***(4)), 171.23 (1C, s, -***C***O-(Ac)). ^{19}F-NMR (376 MHz, $CDCl_3$, 25 °C) δ -58.26 (1F, d, J=6.51 Hz, $\boldsymbol{F_3(a)}$C-), -58.17 (1F, s, $\boldsymbol{F_3(b)}$C-). ^{31}P-NMR (172 MHz, $CDCl_3$, 25 °C) δ 151.02 (2/5 P, q, J=6.65 Hz, ***P***-OC(3')), 151.82 (3/5 P, d, J=1.77 Hz, ***P***-OC(3')).

^{1}H-NMR (400 MHz, $CDCl_3$, 25 °C) of compound **C7a**

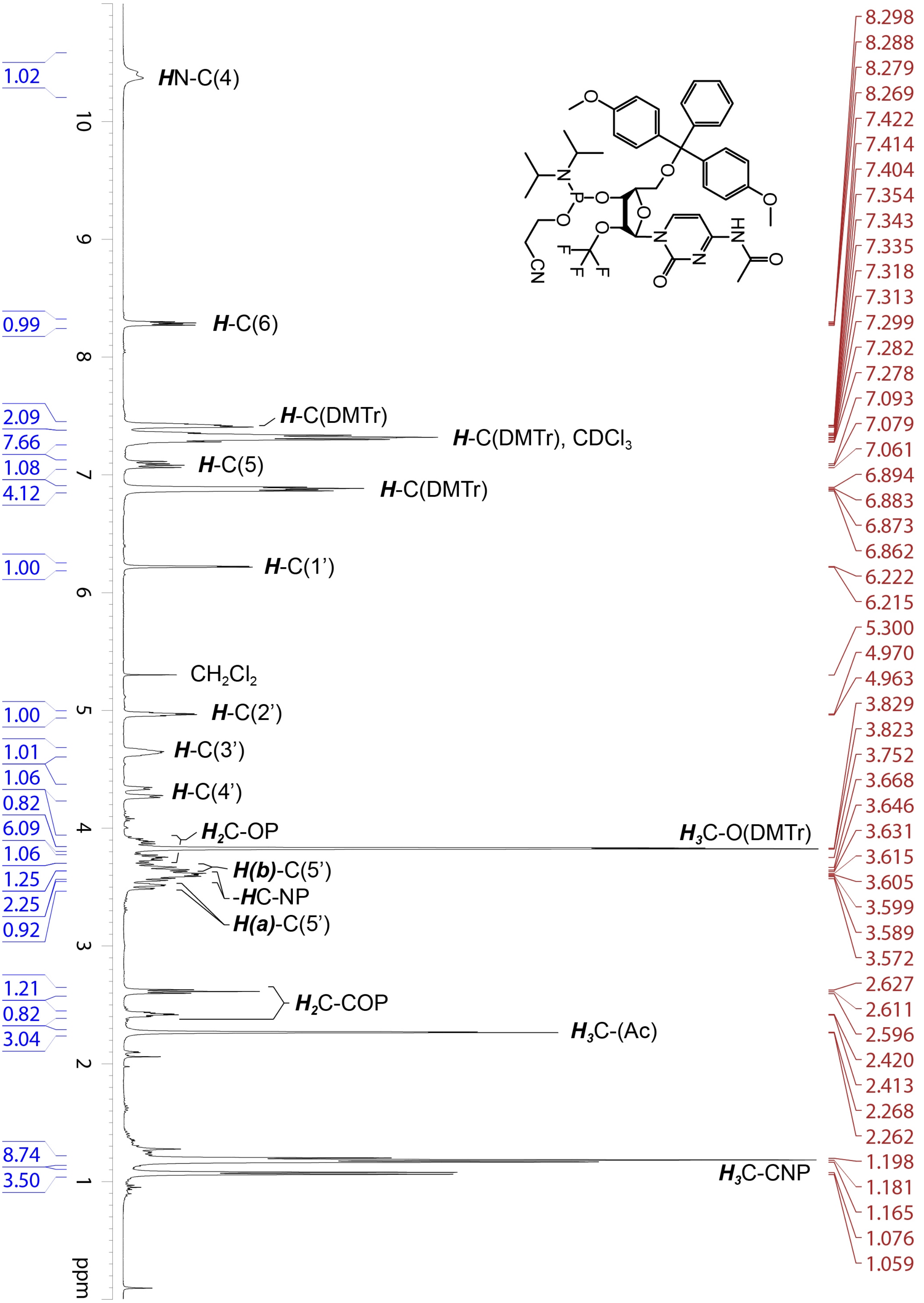

^{13}C-NMR (100 MHz, $CDCl_3$, 25 °C) of compound **C7a**

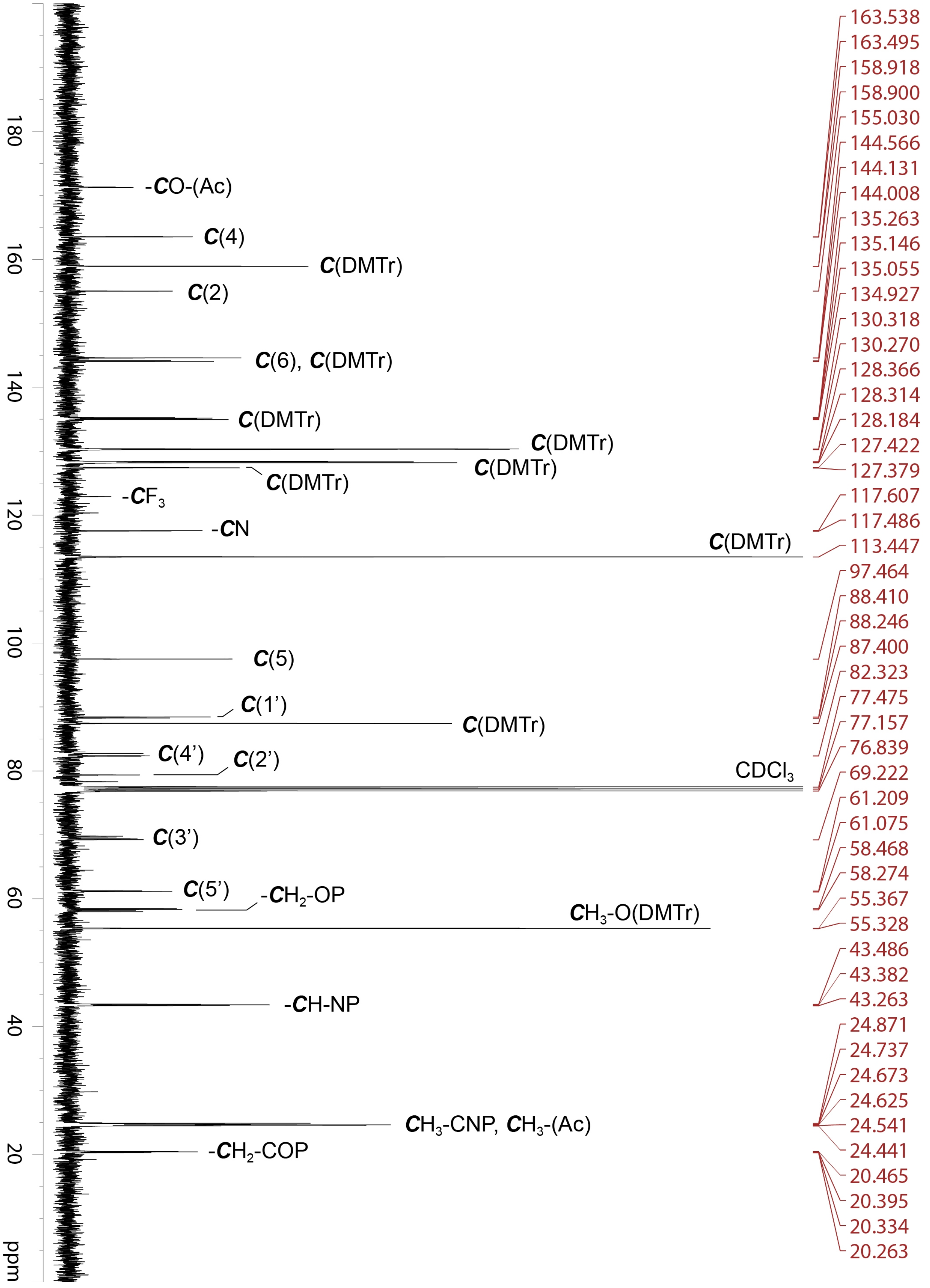

^{19}F-NMR (376 MHz, $CDCl_3$, 25 °C) of compound **C7a**

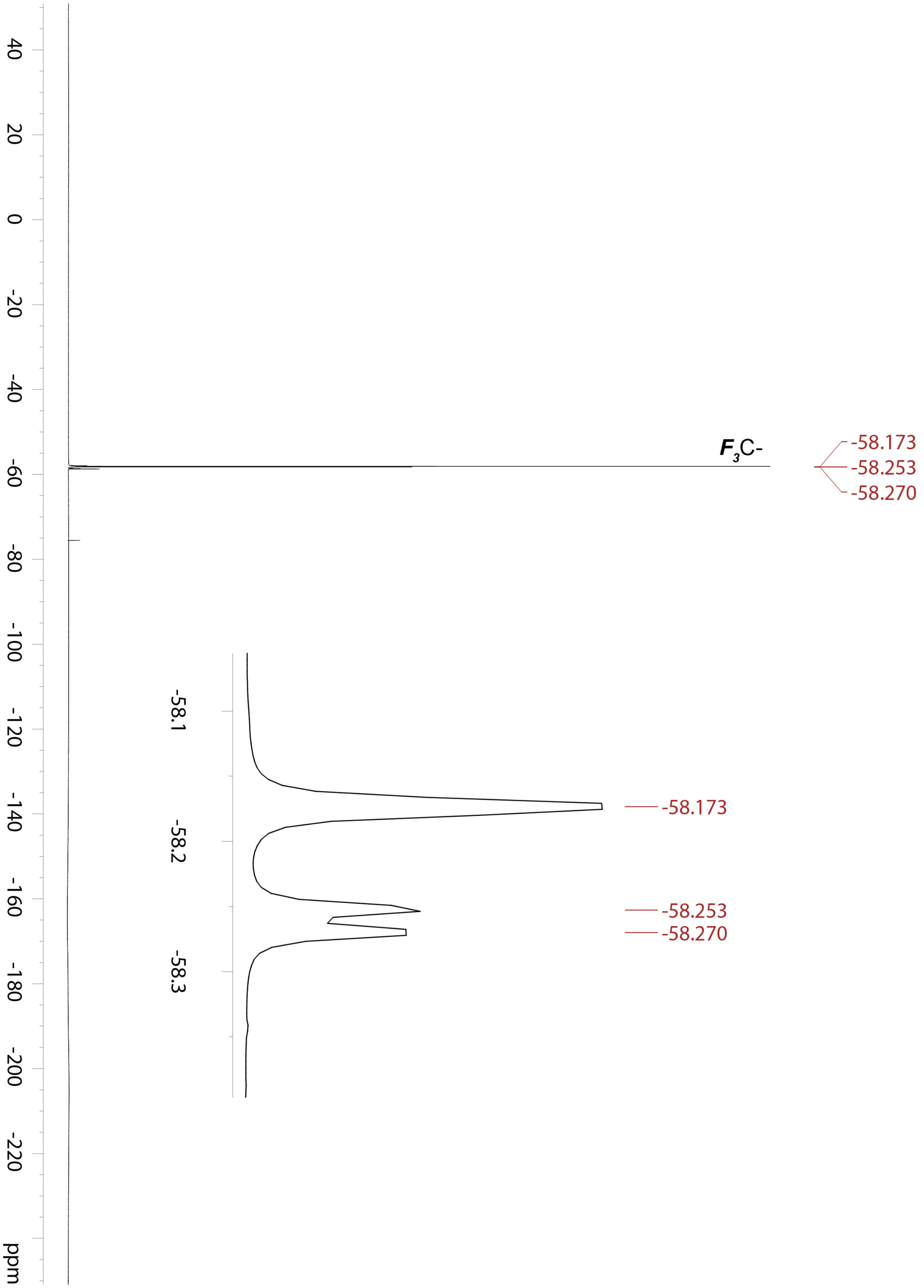

^{31}P-NMR (162 MHz, $CDCl_3$, 25 °C) of compound **C7a**

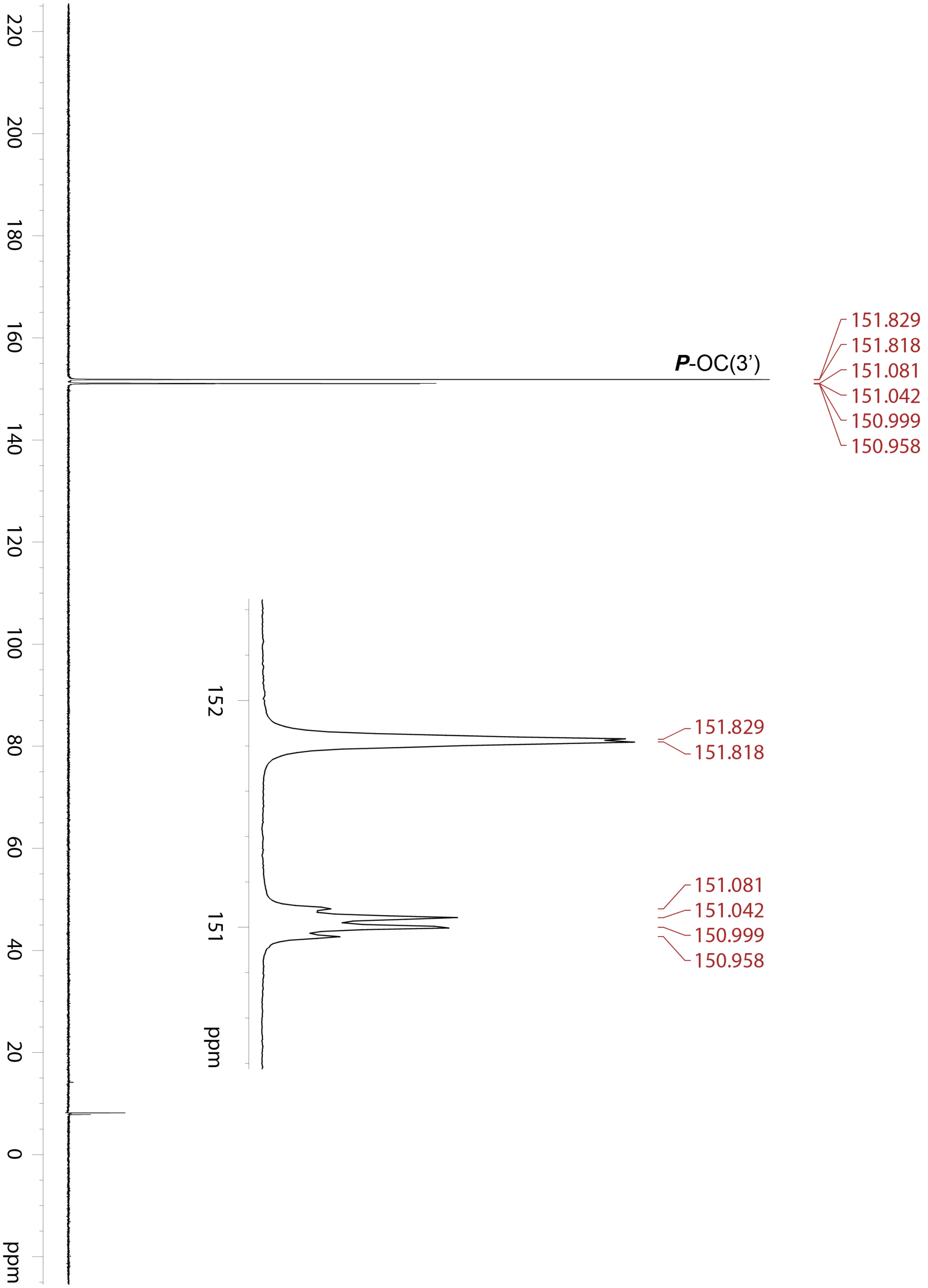

Synthesis of 2'-OCF$_3$ cytidine phosphoramidite C7b

N^4-Benzoyl-2'-*O*-[(methylthio)thiocarbonyl]-3',5'-*O*-(1,1,3,3-tetraisopropyldisiloxane-1,3-diyl)cytidine

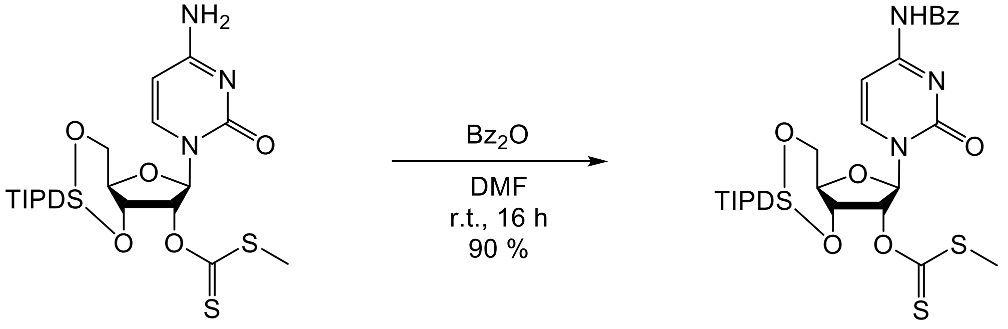

C3 **C4b**

Compound **C3** (2.50 g, 4.34 mmol) was dissolved in dry dimethylformamide (7.9 mL), treated with benzoic anhydride (Bz_2O, 1.08 g, 4.78 mmol) and stirred overnight at ambient temperatures. Upon completion, the reaction mixture was diluted with diethyl ether and washed thrice with sodium bicarbonate solution, twice with brine and once with water. Organic phases were combined, dried over sodium sulfate and concentrated. Crude product was purified by column chromatography on silica gel, eluting with 0 - 3 % methanol in dichloromethane. Yield: 2.64 g of compound **C4b** (90 %) as white foam. TLC: (dichloromethane/methanol, 19/1): R_F = 0.70. ESI-MS: (m/z) $[M+H]^+$ calcd. 680.2310 found: 680.2284. ^{1}H-NMR (400 MHz, $CDCl_3$, 25 °C) δ 1.05 (28H, m, J=8.85 Hz, ***H₃***C-, -***H***C-(TIPDS)), 2.59 (3H, s, ***H₃***C-S), 4.04 (1H, dd, J=2.64, 13.36 Hz, ***H(a)***-C(5')), 4.16 (1H, d, J=9.20 Hz, ***H***-C(4')), 4.29 (1H, d, J=13.44 Hz, ***H(b)***-C(5')), 4.54 (1H, q, J=4.65 Hz, ***H***-C(3')), 5.98 (1H, s, ***H***-C(1')), 6.40 (1H, d, J=4.72 Hz, ***H***-C(2')), 7.52 (3H, t, J=7.60 Hz, ***H***-C(5), ***H***-C(benzoyl)), 7.61 (1H, t, J=7.40 Hz, ***H***-C(benzoyl)), 7.90 (2H, d, J=7.48 Hz, ***H***-C(benzoyl)), 8.19 (1H, d, J=7.36 Hz, ***H***-C(6)), 8.82 (1H, b, ***H***N-C(4)). ^{13}C-NMR (100 MHz, $CDCl_3$, 25 °C) δ 13.15 (4C, q, J=21.43 Hz, -***C***H-(TIPDS)), 17.25 (8C, m, J=11.73 Hz, ***C***H_3-(TIPDS)), 19.38 (1C, s, ***C***H_3-S), 59.85 (1C, s, ***C***(5')), 68.54 (1C, s, ***C***(3')), 82.66 (2C, d, J=22.03 Hz, ***C***(2'), ***C***(4')), 89.92 (1C, s, ***C***(1')), 127.72 (1C, s, ***C***(benzoyl), 129.20 (2C, s, ***C***(benzoyl)), 133.38 (2C, s, ***C***(benzoyl)), 144.62 (1C, s, ***C***(6)), 162.75 (1C, s, ***C***(4)), 214.69 (1C, s, -***C***S_2-).

^{1}H-NMR (400 MHz, $CDCl_3$, 25 °C) of compound **C4b**

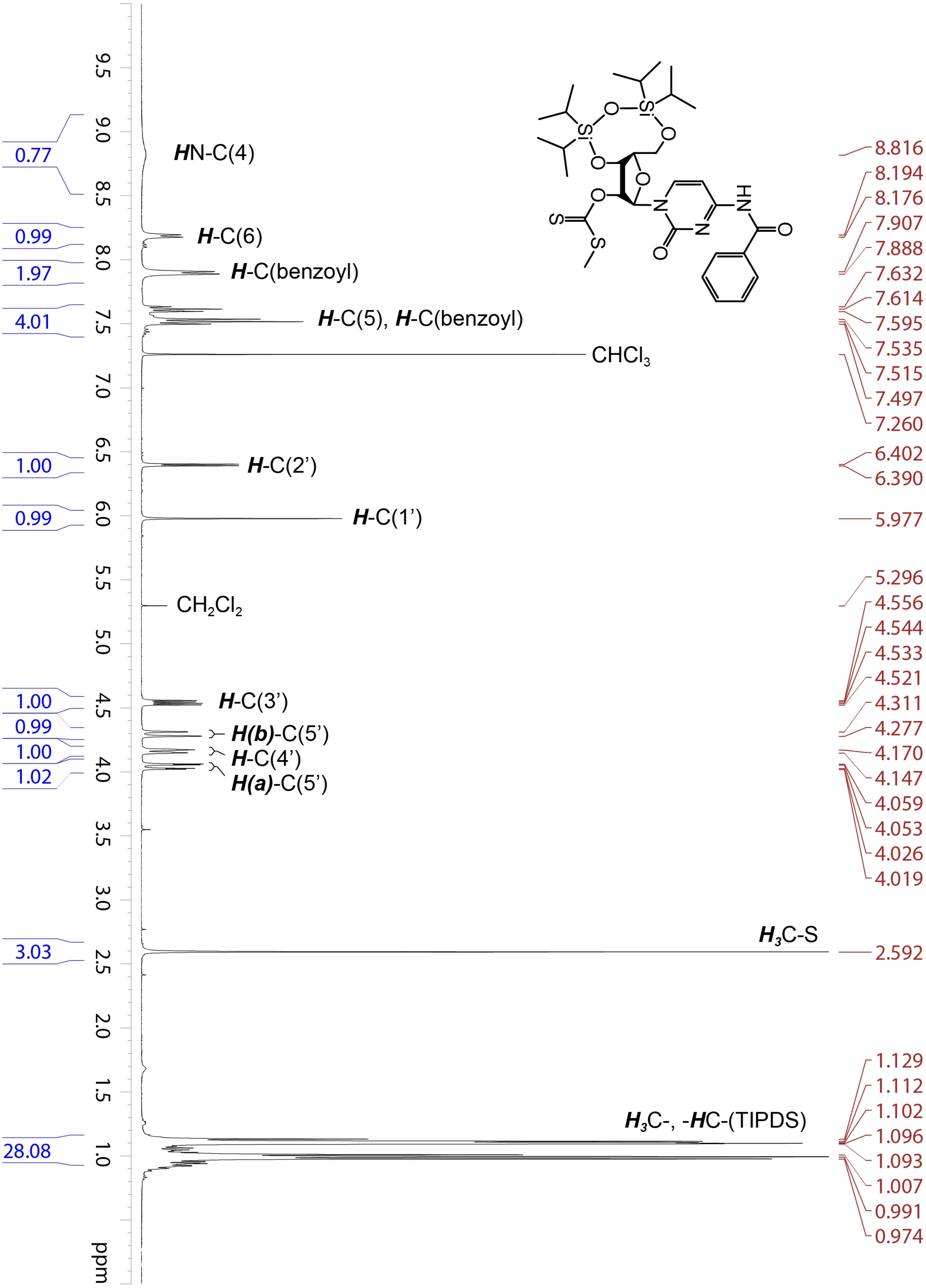

^{13}C-NMR (100 MHz, $CDCl_3$, 25 °C) of compound **C4b**

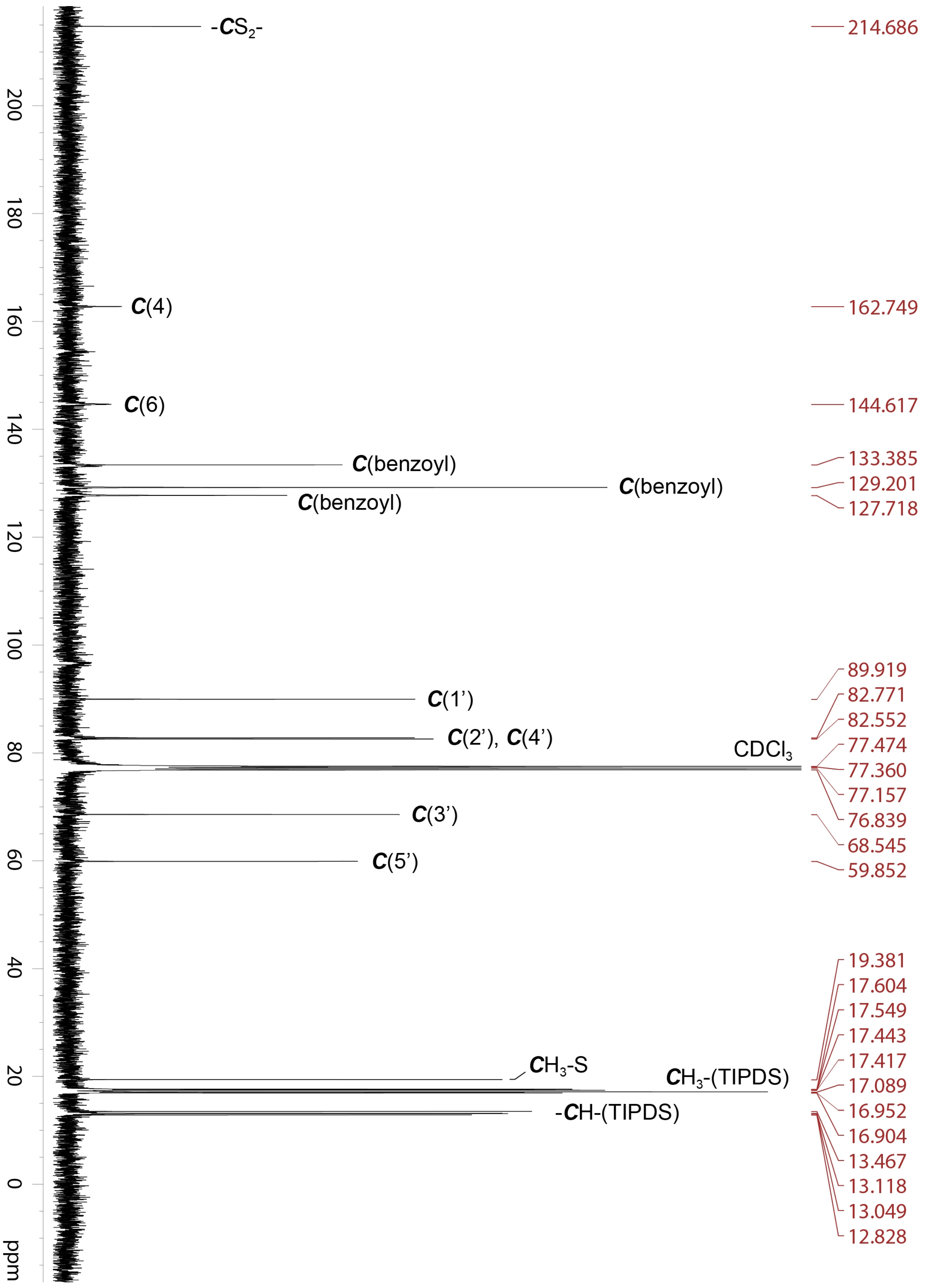

N^4-Benzoyl-2'-*O*-(trifluoromethyl)cytidine

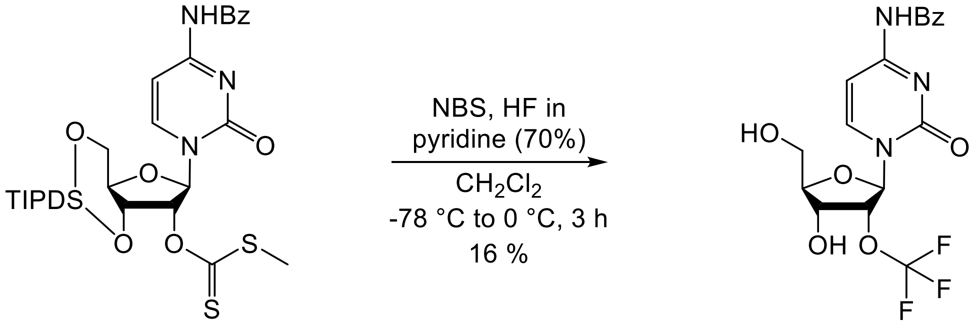

C4b **C5b**

To a suspension of *N*-bromosuccinimide (NBS, 3.46 g, 19.4 mmol) in dichloromethane (12.5 mL), hydrogen fluoride in pyridine complex (70 % HF, 25.2 g, 22.9 mL) was added at -78 °C and subsequently a solution of compound **C4b** (2.64 g, 3.88 mmol) in dichloromethane (12.5 mL) was added to the mixture. The reaction mixture was allowed to warm to 0 °C and stirred for three hours. Upon completion, the reaction mixture was poured carefully into a flask containing saturated bicarbonate solution (250 mL) and sodium thiosulfate solution (10 %, 180 mL) while vigorously stirring. Further bicarbonate solution was added until neutral pH was obtained. The aqueous layer was extracted several times with chloroform/isopropanol (3/1). Organic phases were combined, dried over sodium sulfate and evaporated. Crude product was purified by column chromatography on silica gel, using 1 - 5 % methanol in dichloromethane as eluent. Yield: 255 mg of compound **C5b** (16 %) as white foam. TLC: (dichloromethane/methanol, 9/1): R_F = 0.37. ESI-MS: (m/z) $[M+H]^+$ calcd. 416.1064 found: 416.1045. ^{1}H-NMR (400 MHz, DMSO-d_6, 25 °C) δ 3.72 (2H, qdd, J=2.53, 12.22, 63.82 Hz, ***H_2***-C(5')), 3.98 (1H, m, J=2.93 Hz, ***H***-C(4')), 4.26 (1H, q, J=5.79 Hz, ***H***-C(3')), 4.94 (1H, dd, J=3.58, 4.62 Hz, ***H***-C(2')), 5.33 (1H, t, J=4.98 Hz, ***H***O-C(5')), 5.80 (1H, d, J=6.04 Hz, ***H***O-C(3')), 6.07 (1H, d, J=3.36 Hz, ***H***-C(1')), 7.37 (1H, d, J=7.64 Hz, ***H***-C(5)), 7.52 (2H, t, J=7.64 Hz, ***H***-C(benzoyl)), 7.63 (1H, t, J=7.40 Hz, ***H***-C(benzoyl)), 8.00 (2H, d, J=7.28 Hz, ***H***-C(benzoyl), 8.49 (1H, d, J=7.68 Hz, ***H***-C(6)), 11.32 (1H, s, ***H***N-C(4)). ^{13}C-NMR (100 MHz, $CDCl_3/CD_3OD$ (9/1), 25 °C) δ 60.48 (1C, s, ***C***(5')), 68.21 (1C, s, ***C***(3')), 78.59 (1C, s, ***C***(2')), 85.11 (1C, s, ***C***(4')), 90.65 (1C, s, ***C***(1')), 97.89 (1C, s, ***C***(5)), 122.76 (1C, s, -***C***F_3), 127.99 (2C, s, ***C***(benzoyl)), 128.86 (2C, s, ***C***(benzoyl)), 133.09 (2C, d, J=31.85 Hz, ***C***(benzoyl)), 146.32 (1C, s, ***C***(6)), 156.07 (1C, s, ***C***(2)), 163.68 (1C, s, ***C***(4)), 167.60 (1C, s, -***C***O-(benzoyl). ^{19}F-NMR (376 MHz, DMSO-d_6, 25 °C) δ -56.71 (1F, s, ***F_3***C-).

^{1}H-NMR (400 MHz, DMSO-d_6, 25 °C) of compound **C5b**

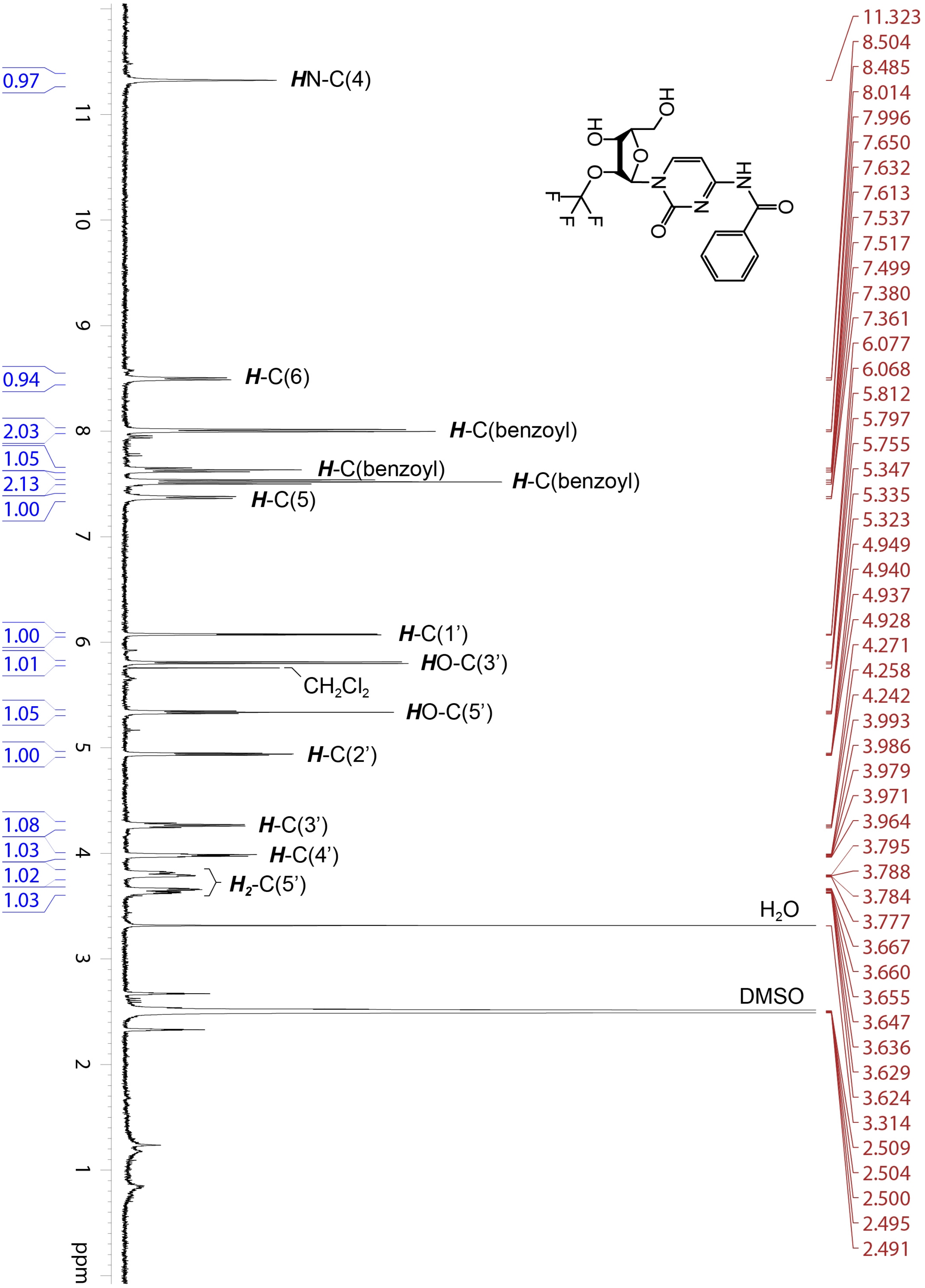

^{13}C-NMR (100 MHz, $CDCl_3/CD_3OD$ (9/1), 25 °C) of compound **C5b**

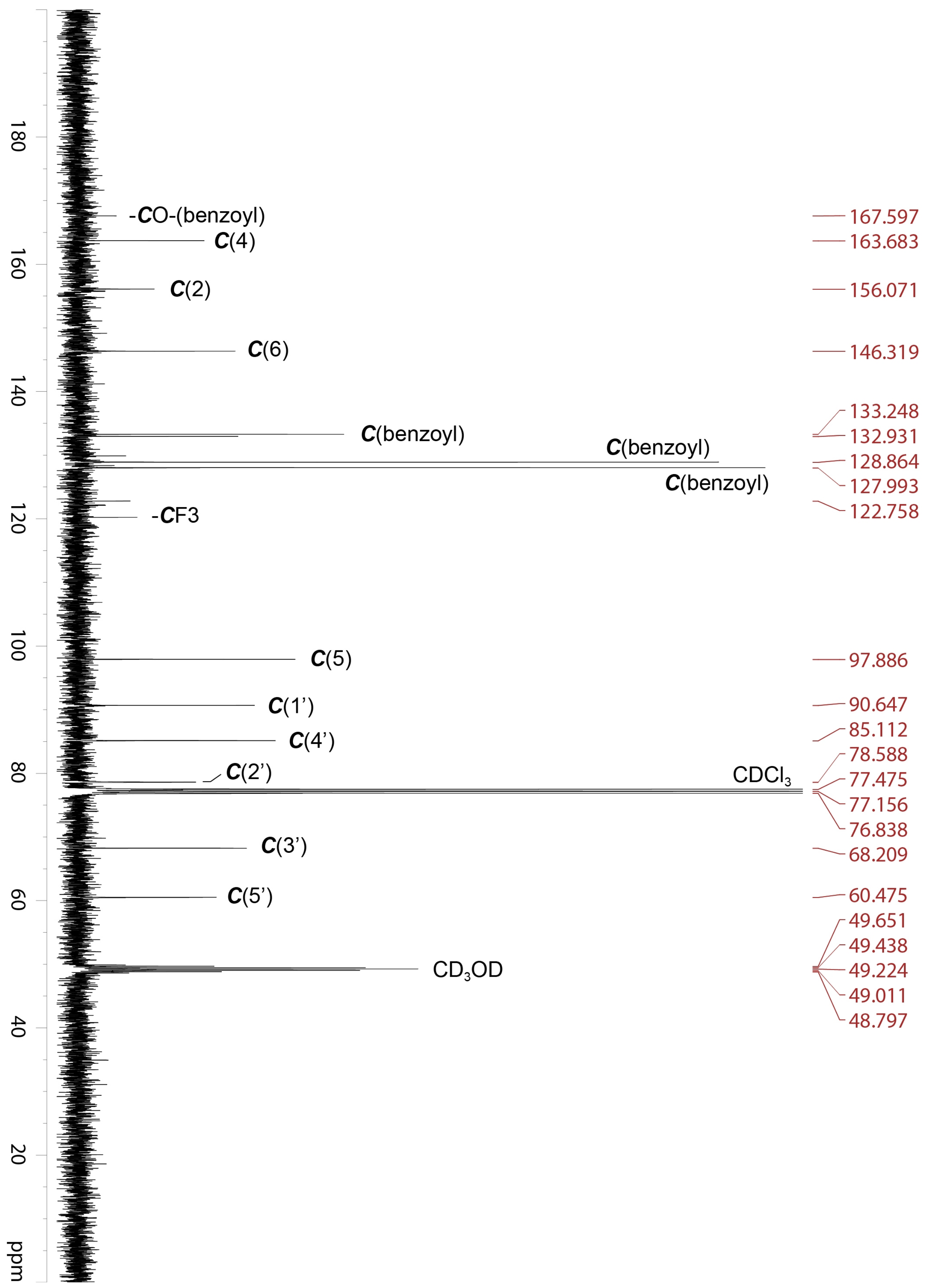

^{19}F-NMR (376 MHz, DMSO-d_6, 25 °C) of compound **C5b**

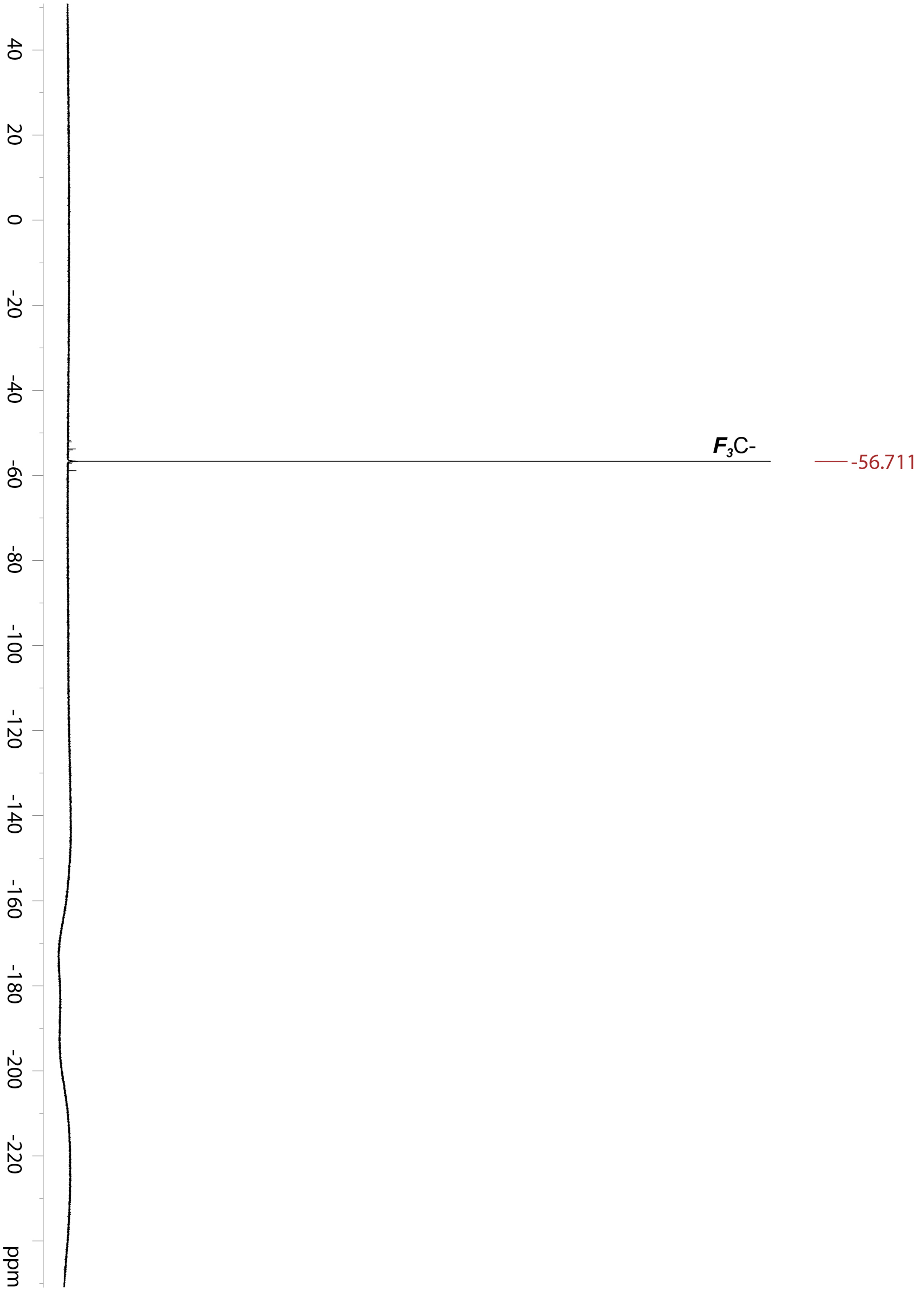

***N*⁴-Benzoyl-5'-*O*-(4,4-dimethoxytrityl)-2'-*O*-(trifluoromethyl)cytidine**

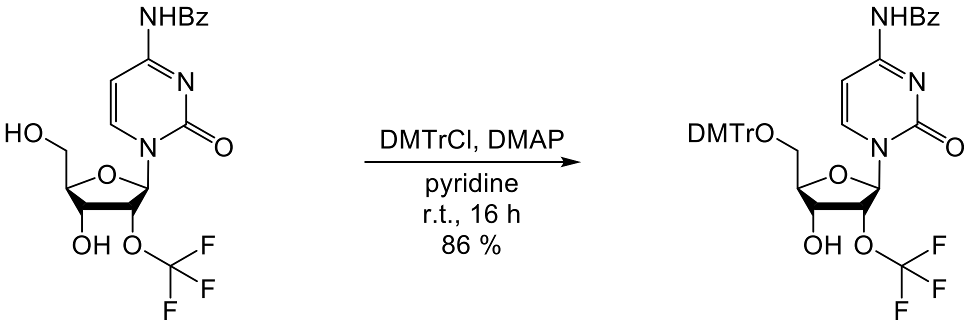

C5b **C6b**

Compound **C5b** (250 mg, 0.60 mmol) was dissolved in dry pyridine (3.6 mL) under argon atmosphere and treated with 4,4'-dimethoxytrityl chloride (DMTrCl, 286 mg, 0.84 mmol) and 4-dimethylaminopyridine (DMAP, 29.4 mg, 0.24 mmol) in four portions over a period of 1 hour at ambient temperatures. The reaction mixture was stirred overnight, quenched with methanol (0.5 mL) and diluted with diethyl ether. Organic phases were washed twice with water, thrice with citric acid solution (5 %) and twice with saturated sodium bicarbonate solution. Combined phases were dried over sodium sulfate, concentrated and coevaporated with toluene two times. Crude product was purified by column chromatography on silica gel, eluting with 20 - 50 % ethyl acetate in hexanes + 2 % triethylamine. Yield: 372 mg of compound **C6b** (86 %) as white foam. TLC: (ethyl acetate/hexanes, 2/1): R_F = 0.57. ESI-MS: (m/z) $[M+H]^+$ calcd. 718.2371 found: 718.2338. ^{1}H-NMR (400 MHz, $CDCl_3$, 25 °C) δ 3.61 (2H, m, J=8.01 Hz, ***H*₂**C-(5')), 3.81 (6H, d, J=2.07 Hz, ***H*₃**C-O(DMTr)), 4.21 (1H, d, J=1.84 Hz, ***H***-C(4')), 4.61 (1H, q, J=6.00 Hz, ***H***-C(3')), 5.06 (1H, s, ***H***-C(2')), 6.18 (1H, q, J=2.16 Hz, ***H***-C(1')), 6.87 (4H, m, J=2.40 Hz, ***H***-C(DMTr)), 7.32 (8H, d, J=7.86 Hz, ***H***-C(5), ***H***-C(DMTr)), 7.42 (2H, t, J=4.11 Hz, ***H***-C(DMTr)), 7.48 (2H, q, J=7.30 Hz, ***H***-C(benzoyl)), 7.59 (1H, m, J=3.33 Hz, ***H***-C(benzoyl)), 7.86 (2H, t, J=6.53 Hz, ***H***-C(benzoyl)), 8.37 (1H, t, J=7.04 Hz, ***H***-C(6)). ^{13}C-NMR (100 MHz, $CDCl_3$, 25 °C) δ 55.26 (2C, s, ***C***H_3-O(DMTr)), 61.28 (1C, s, ***C***(5')), 68.34 (1C, s, ***C***(3')), 79.82 (1C, s, ***C***(2')), 82.59 (1C, s, ***C***(4')), 87.41 (1C, d, J=3.37 Hz, ***C***(DMTr)), 88.30 (1C, s, ***C***(1')), 113.43 (4C, s, ***C***(DMTr)), 127.31 (1C, s, -***C***F_3), 127.72 (1C, s, ***C***(DMTr)), 128.19 (4C, d, J=8.18 Hz, ***C***(DMTr)), 128.97 (2C, d, J=3.19 Hz, ***C***(benzoyl)), 130.08 (4C, d, J=8.58 Hz, ***C***(DMTr), ***C***(benzoyl)), 133.16 (2C, s, ***C***(benzoyl)), 135.27 (2C, t, J=15.99 Hz, ***C***(DMTr)), 143.93 (1C, s, ***C***(DMTr)), 144.68 (1C, s, ***C***(6)), 158.79 (2C, s, ***C***(DMTr)), 162.71 (1C, s, ***C***(4)). ^{19}F-NMR (376 MHz, $CDCl_3$, 25 °C) δ -58.26 (3F, d, J=14.80 Hz, ***F*₃**C-).

^{1}H-NMR (400 MHz, $CDCl_3$, 25 °C) of compound **C6b**

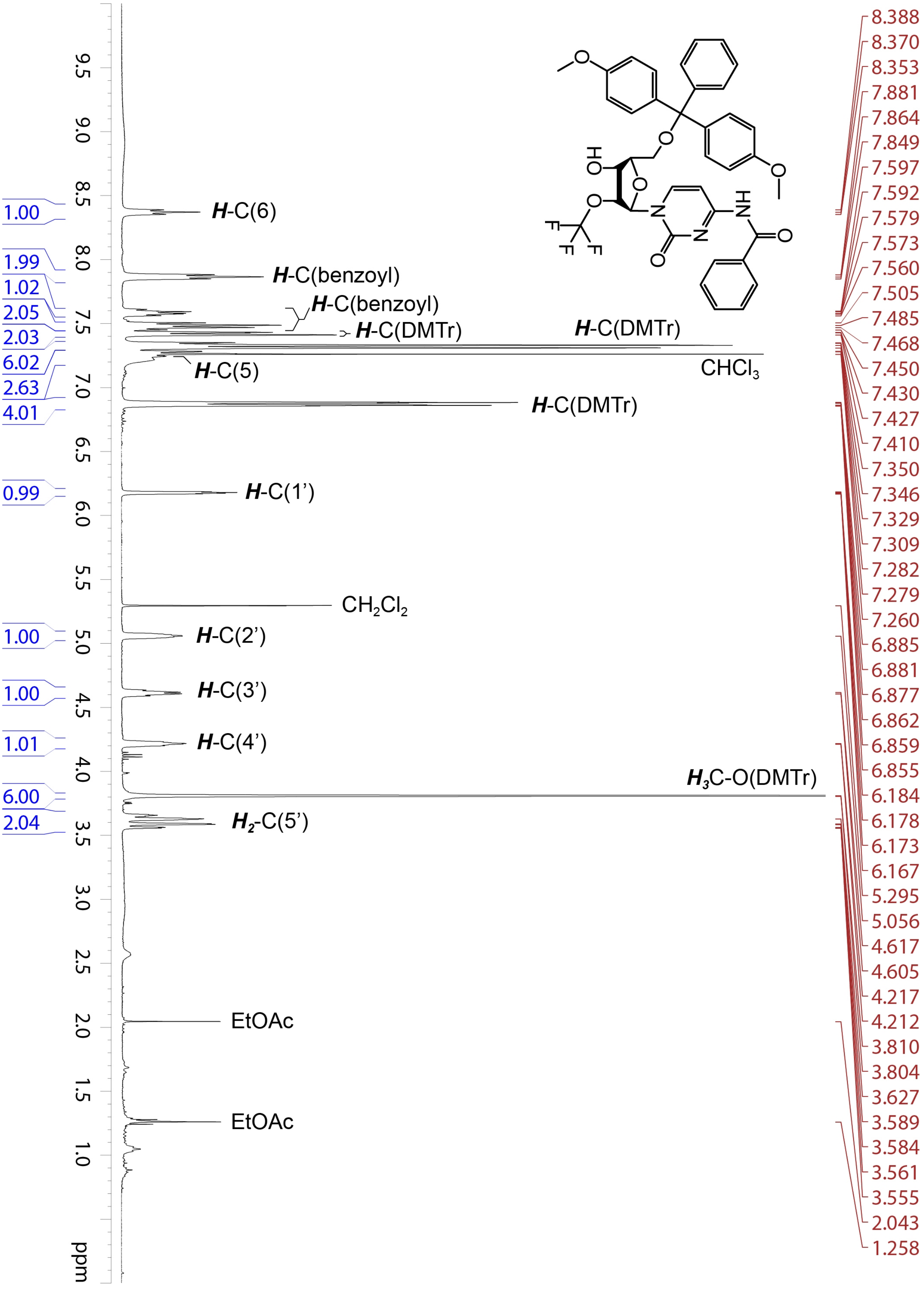

^{13}C-NMR (100 MHz, $CDCl_3$, 25 °C) of compound **C6b**

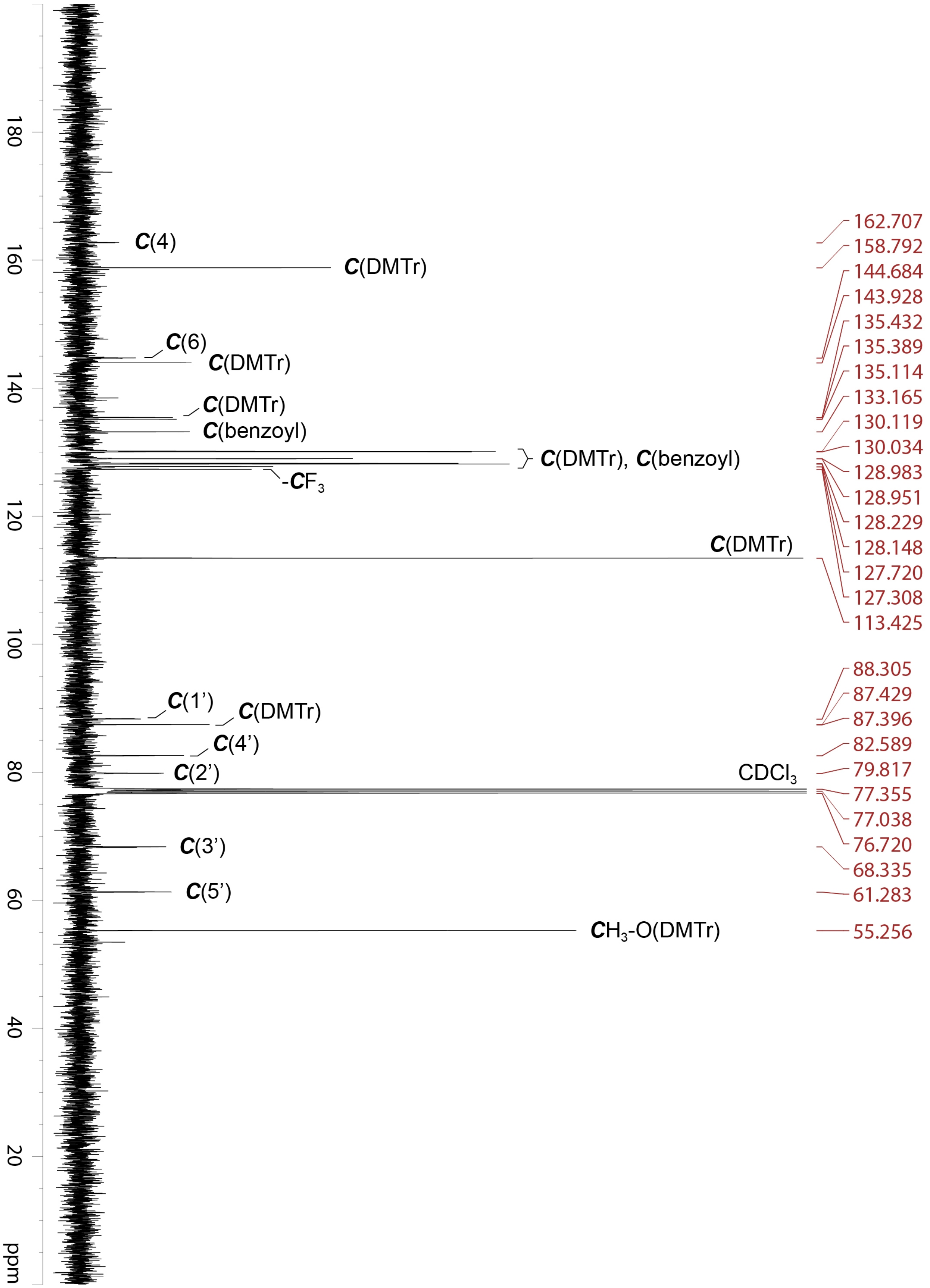

^{19}F-NMR (376 MHz, $CDCl_3$, 25 °C) of compound **C6b**

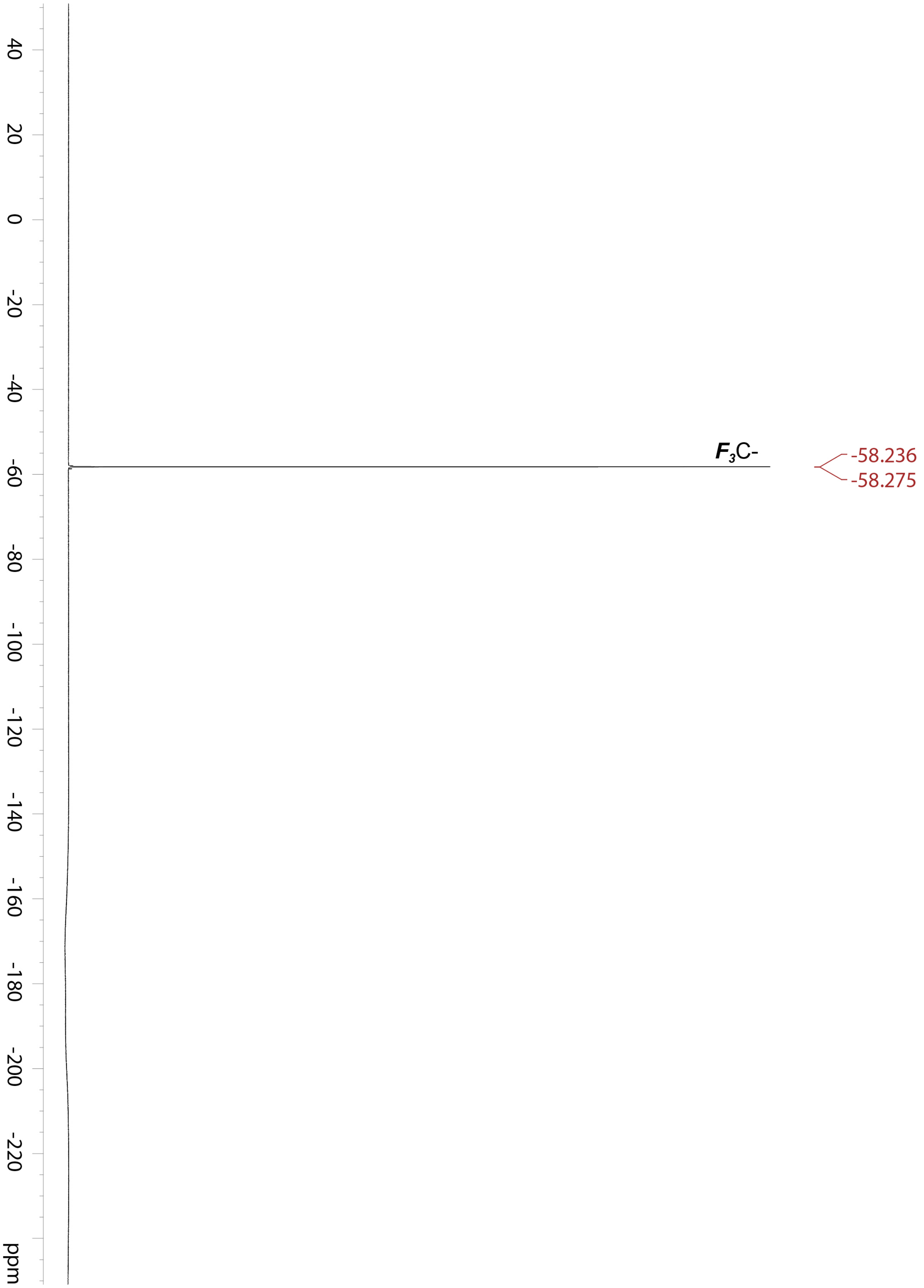

***N*⁴-Benzoyl-2'-*O*-trifluoromethyl-5'-*O*-(4,4-dimethoxytrityl)cytidine 3'-(2-cyanoethyl)-*N,N*-diisopropylphosphoramidite**

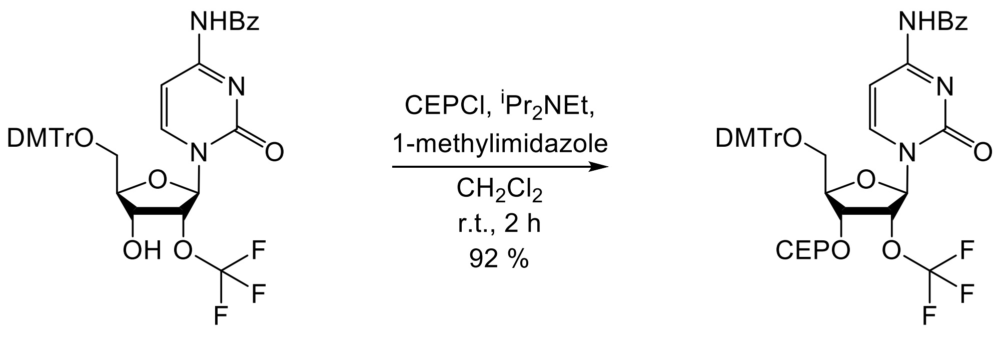

C6b **C7b**

Compound **C6b** (360 mg, 0.50 mmol) was dissolved in anhydrous dichloromethane (3.2 mL) and treated with diisopropylethylamine (0.58 mL, 433 mg, 3.76 mmol), 2-cyanoethyl-*N,N*-diisopropylchloro phosphoramidite (CEPCl, 0.28 mL, 297 mg, 1.25 mmol) and 1-methyl imidazole (20.0 µL, 20.6 mg, 0.25 mmol). The reaction mixture was stirred for 2 hours at ambient temperatures and TLC showed complete conversion of starting material. The reaction was quenched with methanol (0.5 mL) and all volatiles were evaporated. Crude product was purified by column chromatography on silica gel, using 25 - 75 % ethyl acetate in hexanes + 2 % triethylamine as eluent. Yield: 424 mg of a 5:4 mixture of diastereomers of compound **C7b** (92 %) as white foam. TLC: (ethyl acetate/hexanes, 1/1): R_F = 0.33, 0.46. ESI-MS: (m/z) $[M+H]^+$ calcd. 918.3449 found: 918.3404. ^{1}H-NMR (400 MHz, $CDCl_3$, 25 °C) δ 1.06 (3H, d, J=6.77 Hz, ***H₃***C-CNP), 1.17 (9H, m, J=2.40 Hz, ***H₃***C-CNP), 2.40 (1H, m, J=2.88 Hz, -***H₂***C-COP), 2.61 (1H, q, J=4.33 Hz, -***H₂***C-COP), 3.52 (2H, m, J=4.08 Hz, ***H₂***-C(5')), 3.61 (2H, m, J=3.35 Hz, -***H***C-NP), 3.73 (1H, m, J=3.61 Hz, -***H₂***C-OP), 3.82 (6H, d, J=3.80 Hz, ***H₃***C-O(DMTr)), 3.89 (1H, m, J=4.00 Hz, -***H₂***C-OP), 4.30 (1H, dd, J=6.49, 29.46 Hz, ***H***-C(4')), 4.67 (1H, m, J=5.60 Hz, ***H***-C(3')), 5.01 (1H, m, J=2.38 Hz, ***H***-C(2')), 6.24 (1H, q, J=3.32 Hz, ***H***-C(1')), 6.87 (4H, m, J=3.36 Hz, ***H***-C(DMTr)), 7.11 (1H, m, J=3.49 Hz, ***H***-C(5)), 7.31 (7H, m, J=2.68 Hz, ***H***-C(DMTr)), 7.42 (2H, t, J=3.90 Hz, ***H***-C(DMTr)), 7.50 (2H, t, J=7.55 Hz, ***H***-C(benzoyl)), 7.60 (1H, t, J=7.37 Hz, ***H***-C(benzoyl)), 7.89 (2H, d, J=7.40 Hz, ***H***-C(benzoyl)), 8.30 (1H, d, J=7.35 Hz, ***H***-C(6)), 8.65 (1H, b, ***H***N-C(4)). ^{13}C-NMR (100 MHz, $CDCl_3$, 25 °C) δ 20.40 (1C, q, J=8.30 Hz, -***C***H_2-COP), 24.62 (4C, m, J=4.91 Hz, ***C***H_3-CNP), 43.42 (2C, t, J=12.46 Hz, -***C***H-NP), 55.37 (2C, d, J=3.85 Hz, ***C***H_3-O(DMTr)), 58.22 (1C, q, J=17.83 Hz, -***C***H_2-OP), 61.27 (1C, d, J=24.26 Hz, ***C***(5')), 69.67 (1C, q, J=23.05 Hz, ***C***(3')), 78.73 (1C, d, J=104.88 Hz, ***C***(2')), 82.58 (1C, t, J=24.64 Hz, ***C***(4')), 87.46 (1C, d, J=2.81 Hz, ***C***(DMTr)), 88.39 (1C, d, J=24.95 Hz, ***C***(1')), 113.48 (4C, s, ***C***(DMTr)), 117.56 (1C, d, J=17.52 Hz, -***C***N), 127.45 (1C, d, J=6.42 Hz, -***C***F_3), 127.72 (1C, s, ***C***(DMTr)), 128.21 (2C, d, J=2.12 Hz, ***C***(benzoyl), 128.45 (2C, d, J=6.44 Hz, ***C***(DMTr)), 129.12 (2C, s, ***C***(DMTr)), 130.33 (4C, q, J=3.99 Hz, ***C***(DMTr), ***C***(benzoyl)), 133.25 (2C, d, J=4.08 Hz, ***C***(benzoyl), 135.22 (2C, q, J=11.89 Hz, ***C***(DMTr)), 143.99 (1C, d, J=11.58 Hz, ***C***(6)), 158.94 (2C, d, J=4.12 Hz, ***C***(DMTr)), 162.48 (1C, s, ***C***(4)). ^{19}F-NMR (376 MHz, $CDCl_3$, 25 °C) δ -58.21 (3F, d, J=43.65 Hz, ***F₃***C-). ^{31}P-NMR (162 MHz, $CDCl_3$, 25 °C) δ 151.01 (5/9 P, q, J=6.34 Hz, ***P***-OC(3')), 151.81 (4/9 P, d, J=1.83 Hz, ***P***-OC(3')).

^{1}H-NMR (400 MHz, $CDCl_3$, 25 °C) of compound **C7b**

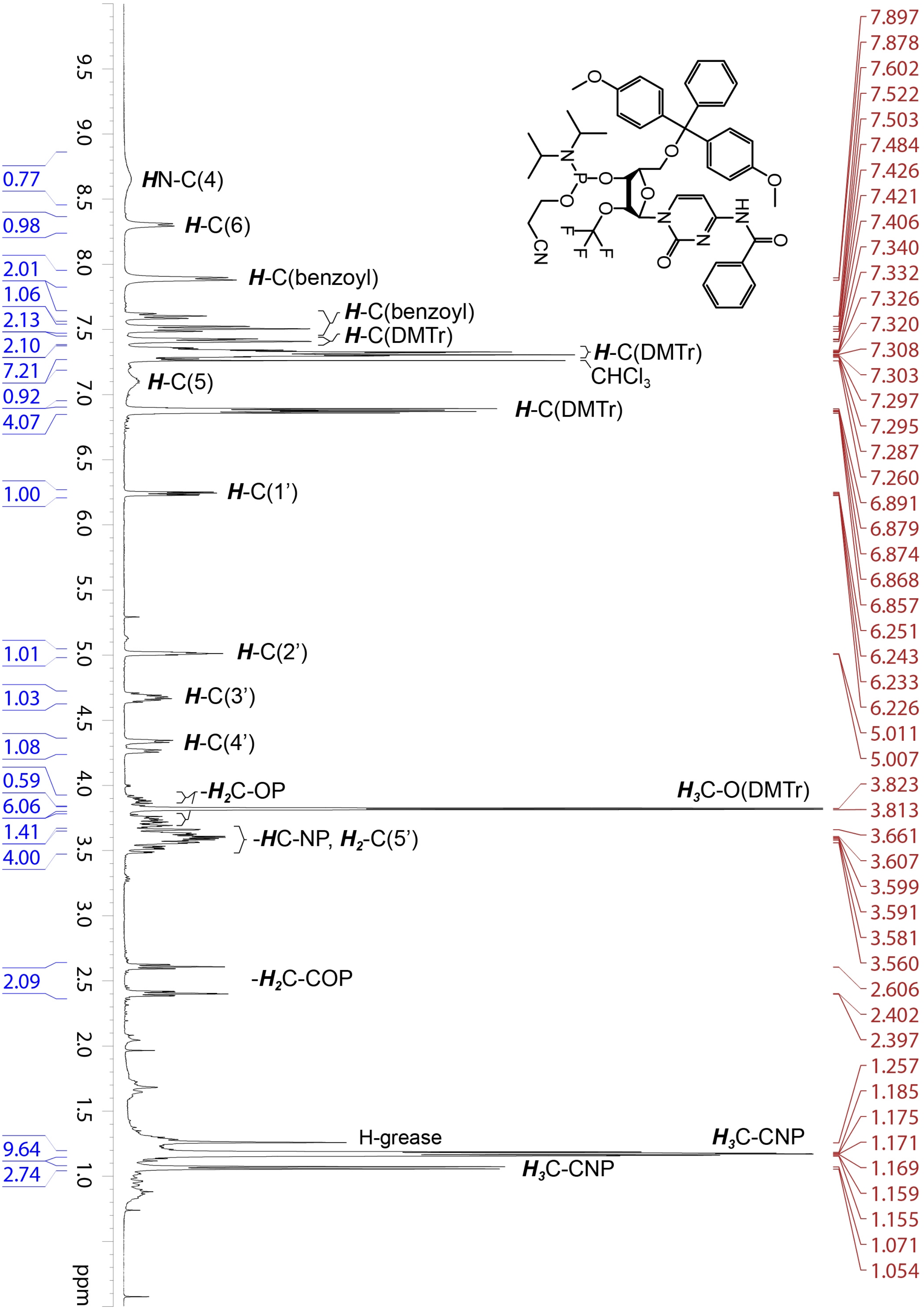

^{13}C-NMR (100 MHz, $CDCl_3$, 25 °C) of compound **C7b**

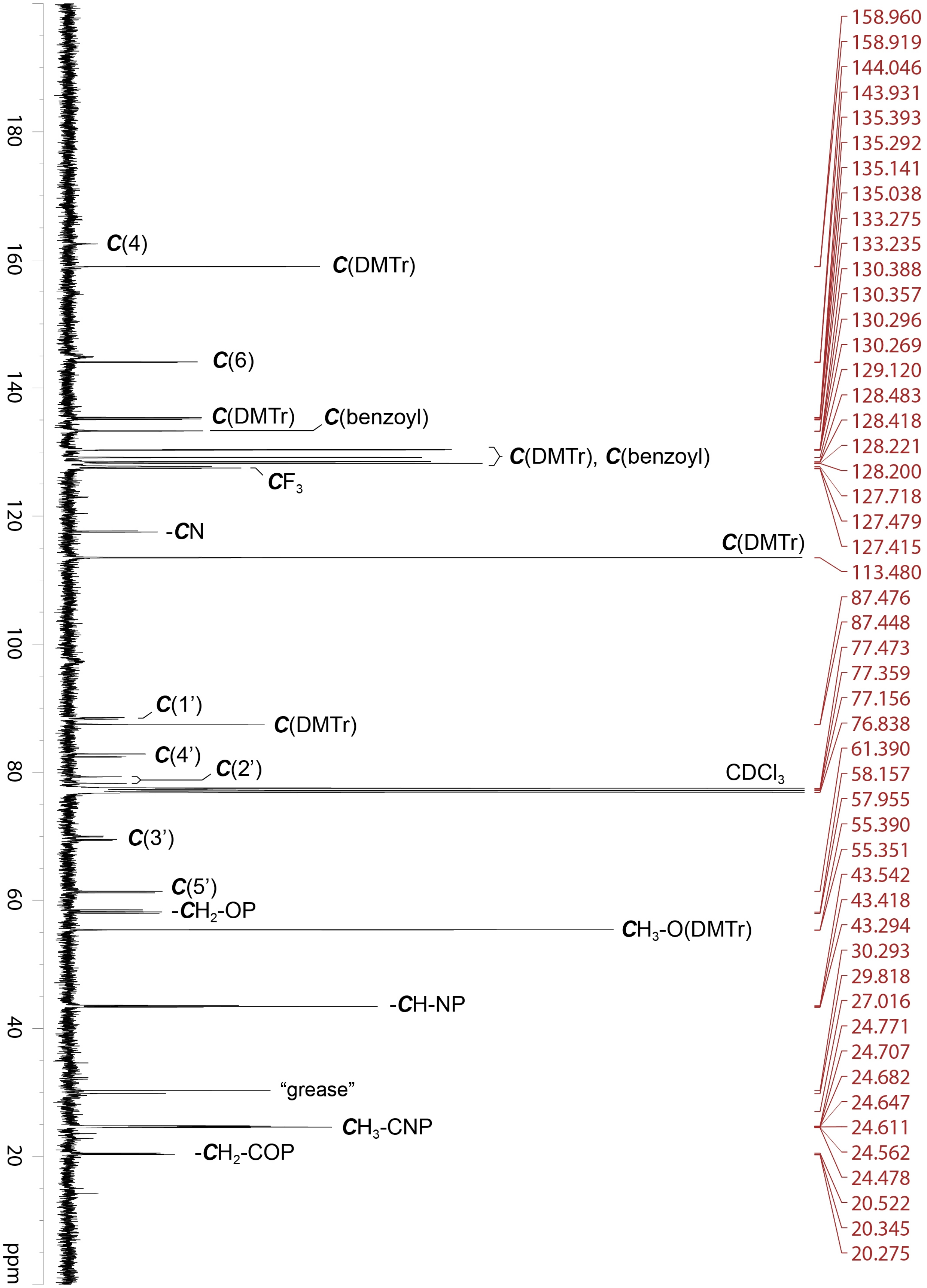

^{19}F-NMR (376 MHz, $CDCl_3$, 25 °C) of compound **C7b**

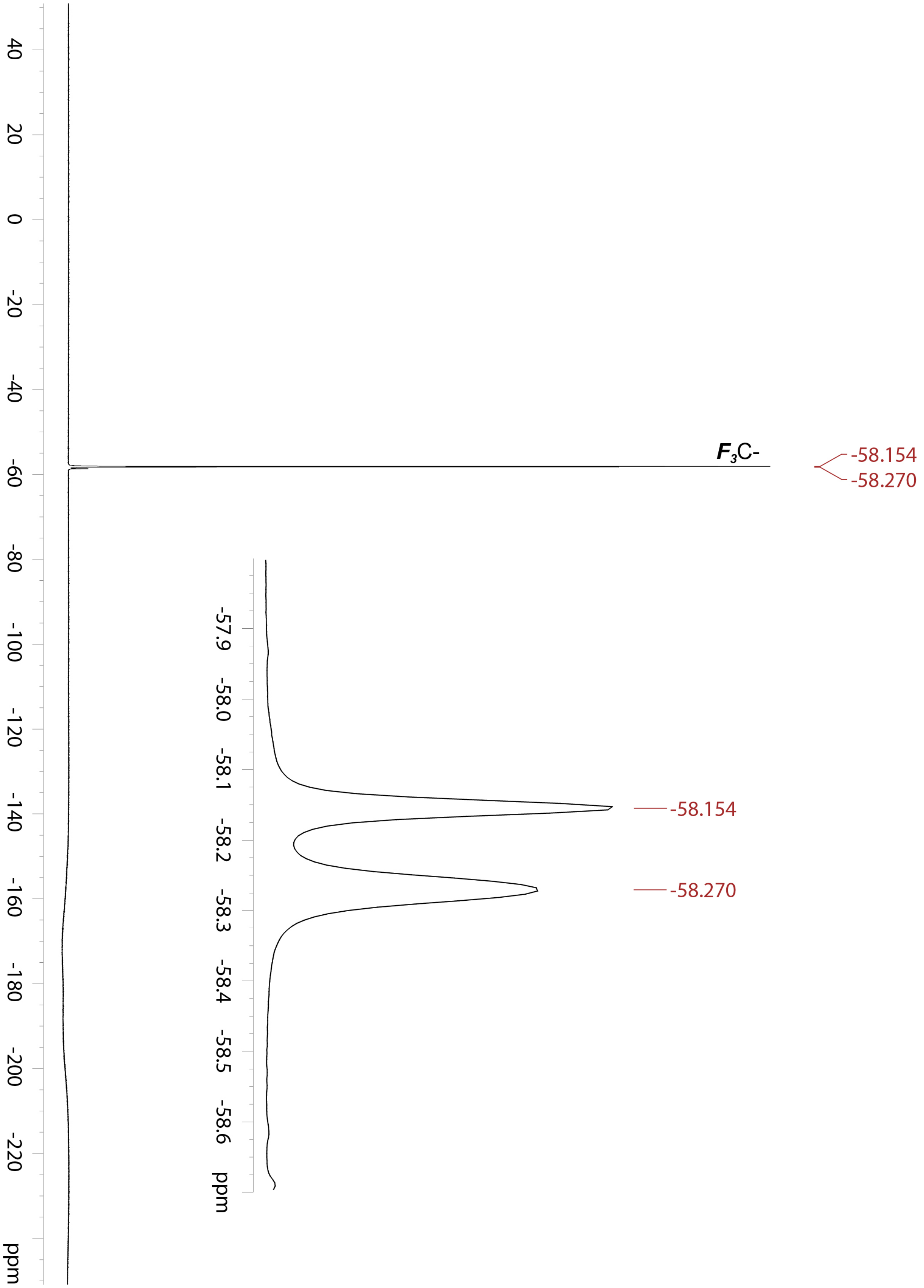

^{31}P-NMR (162 MHz, $CDCl_3$, 25 °C) of compound **C7b**

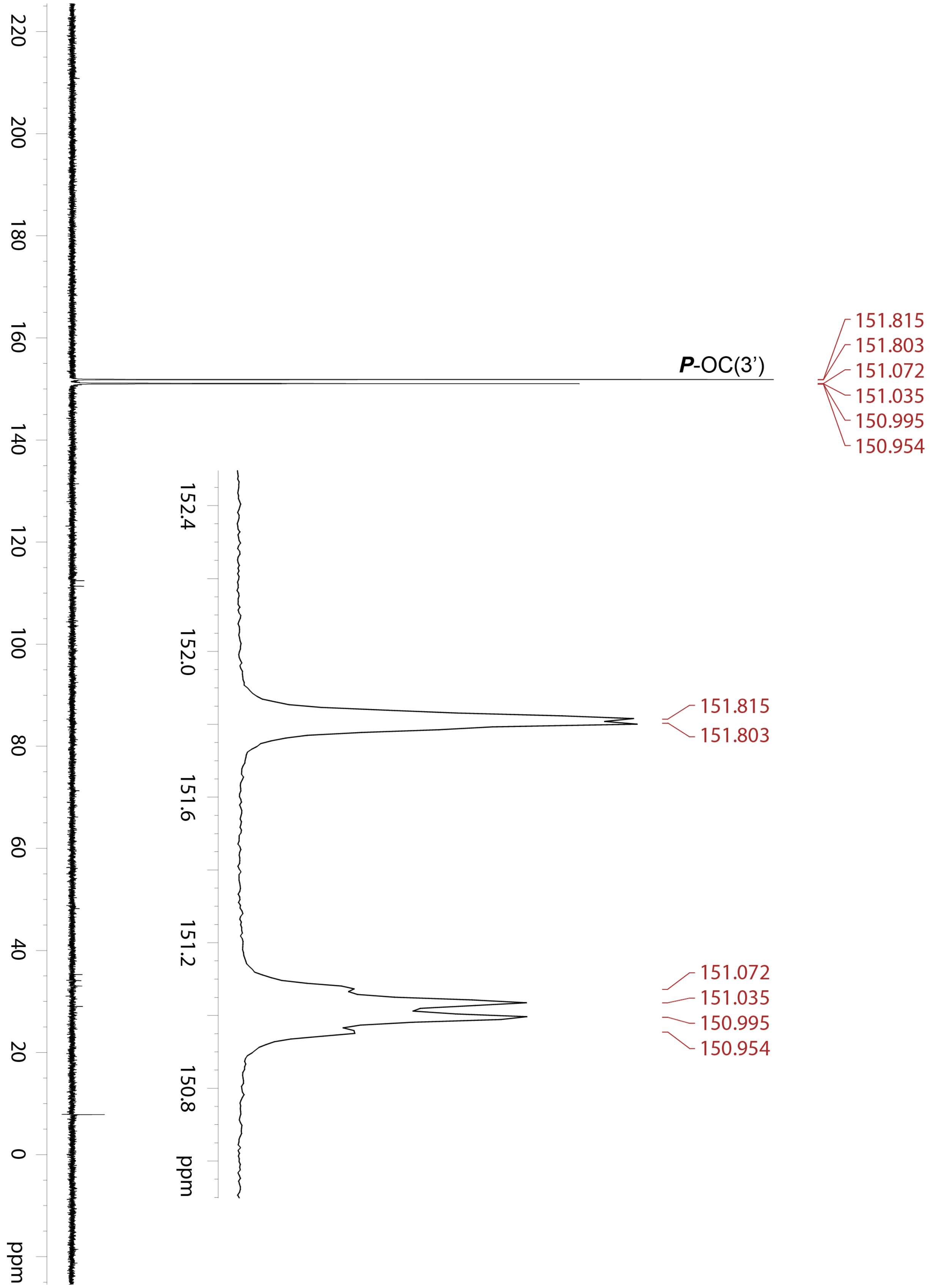

Supporting Table 1. Selection of synthesized 2'-OCF_3 & 2'-F modified RNAs.

Sequence (5' → 3')	nt	Molecular weight	
		calc.	found
UUA$^{2'-OCF3}$GCG	6	1943.2	1943.1
GGUCGA$^{2'-OCF3}$CC	8	2592.6	2592.5
GGC$^{2'-OCF3}$UAGCC	8	2592.6	2592.4
GGCAGA$^{2'-OCF3}$GGC	9	3000.9	3001.0
GGCAGA$^{2'-F}$GGC	9	2934.9	2935.0
GAA$^{2'-OCF3}$GGGCAACCUUCG	15	4882.0	4882.8
GAAGGGCA$^{2'-OCF3}$ACCUUCG	15	4882.0	4882.5
GAAGGGCA$^{2'-F}$ACCUUCG	15	4816.0	4816.3
GAAGGGCAACC$^{2'-OCF3}$UUCG	15	4882.0	4882.1
GAA$^{2'-OCF3}$GGGCAACCUUCGGGUUG	21	6530.0	6531.4
GAAGGGCAAC$^{2'-OCF3}$CUUCGGGUUG	21	6530.0	6530.5
UGCUCCUAGUACGAGAGGACC$^{2'-OCF3}$GGAGUG	27	8795.3	8796.4
UGCUCCUAGUACGAGAGGACCGGA$^{2'-OCF3}$GUG	27	8795.3	8796.4
CUGGGUCGCAGUAACCCCAGUUAACAA$^{2'-OCF3}$AACAAGG	34	10995.7	10997.0
CUGGGUCGCAGUAACCCCAGUUAACAAAACAA$^{2'-OCF3}$GG	34	10995.7	10997.0
AGAUGUGCCAGCAAAA$^{2'-OCF3}$CCAUCUUUAAAAAACUGG	34	10950.7	10949.8
AGAUGUGCCAGCAAAACCAUCUUUAAAAAA$^{2'-OCF3}$CUGG	34	10950.7	10950.0

Supporting Table 2. X-ray data collection and crystallographic refinement statistics

		SRL-C2667-OCF3	SRL-A2670-OCF3
PDB ID		6ZYB	6ZXZ
Space group		$P4_3$	C2
a (Å)		29.50	62.4
b (Å)		29.50	75.27
c (Å)		77.13	52.98
ß		90.00°	105.60°
Beamline		PX III - X06DA	PX III – X06DA
Resolution range (Å)		35.0 – 0.9	35.0 – 2.4
Number of frames		5400	1800
Oscillation angle		0.2°	0.2°
Wavelength (Å)		1.00	1.00
Average redundancy		39.1	3.5
Completeness [1]		100.0% (100.0%)	99.5% (98.6%)
CC1/2 [1]		100.0 (55.5)	99.0 (35.9)
Average I/σ [1]		45.0 (1.4)	6.2 (1.0)
ISa		24.6	19.2
R/Rfree		13.1 / 14.9	21.2 / 23.2
Coordinate error (Å)		0.13	0.39
Atoms	RNA	663	1698
	Water	146	51
	Ions	0	8
Mean B (Å²)	RNA	17.5	36.2
	Water	32.2	32.1
	Ions	-	61.0

[1] Values for last resolution shell are shown in parenthesis

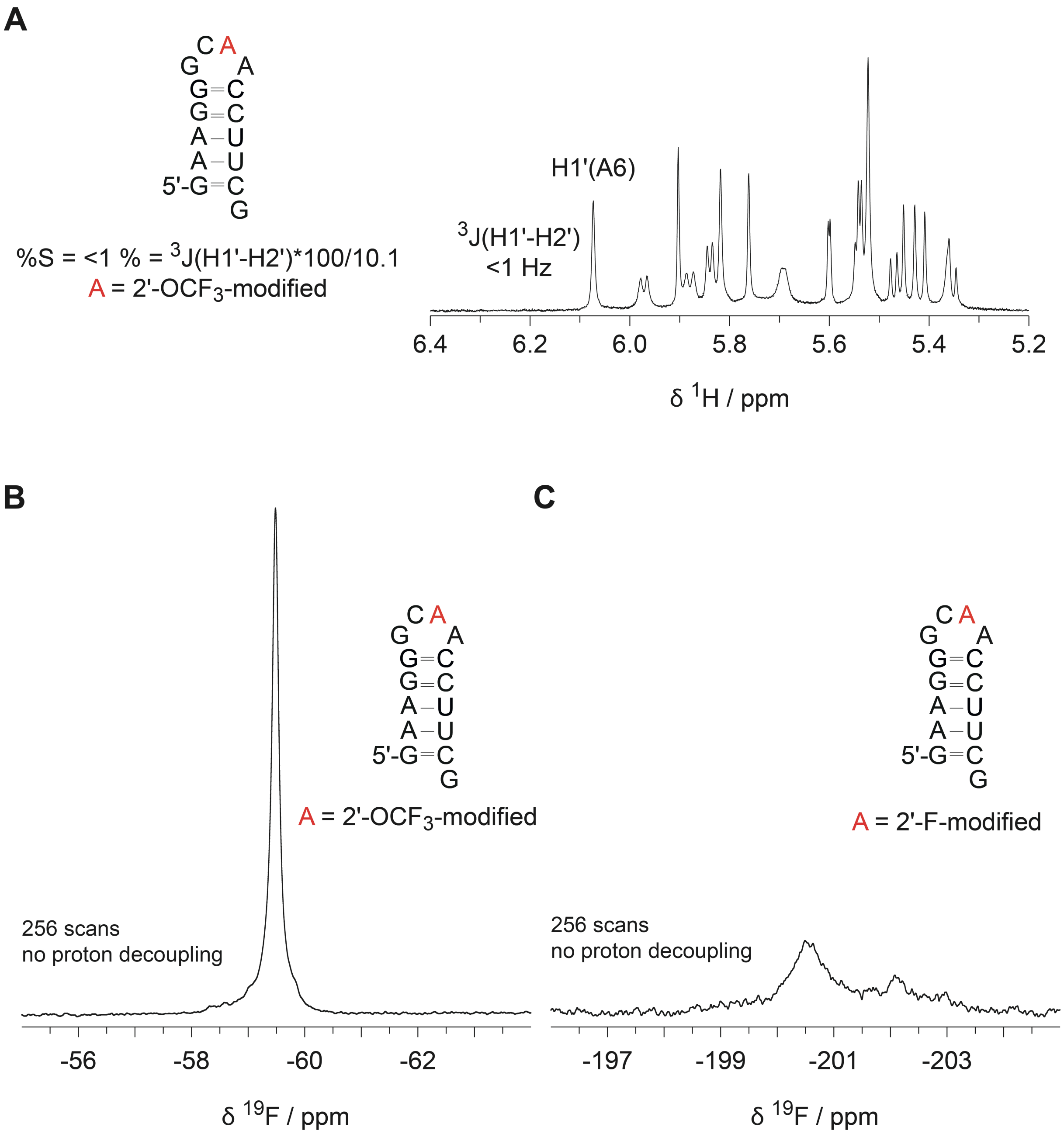

Supporting Figure 1. NMR spectra of fluorine modified hairpin RNA (600 MHz Bruker). ^{1}H-NMR of 2'-OCF_3 modified hairpin (**A**); ^{3}J(H1'-H2') is smaller than 1 Hz, characteristic of C3'-endo conformation in the loop. ^{19}F-NMR sensitivity comparison of RNA hairpin with 2'-OCF_3 modification (**B**) and with 2'-F modification (**C**). c_{RNA} = 0.3; 15 mM sodium cacodylate, 25 mM NaCl, 3 mM NaN_3, H_2O/D_2O = 9/1, pH 6.5., 298 K. (S (South), C2'-endo; N (North), C3'-endo).

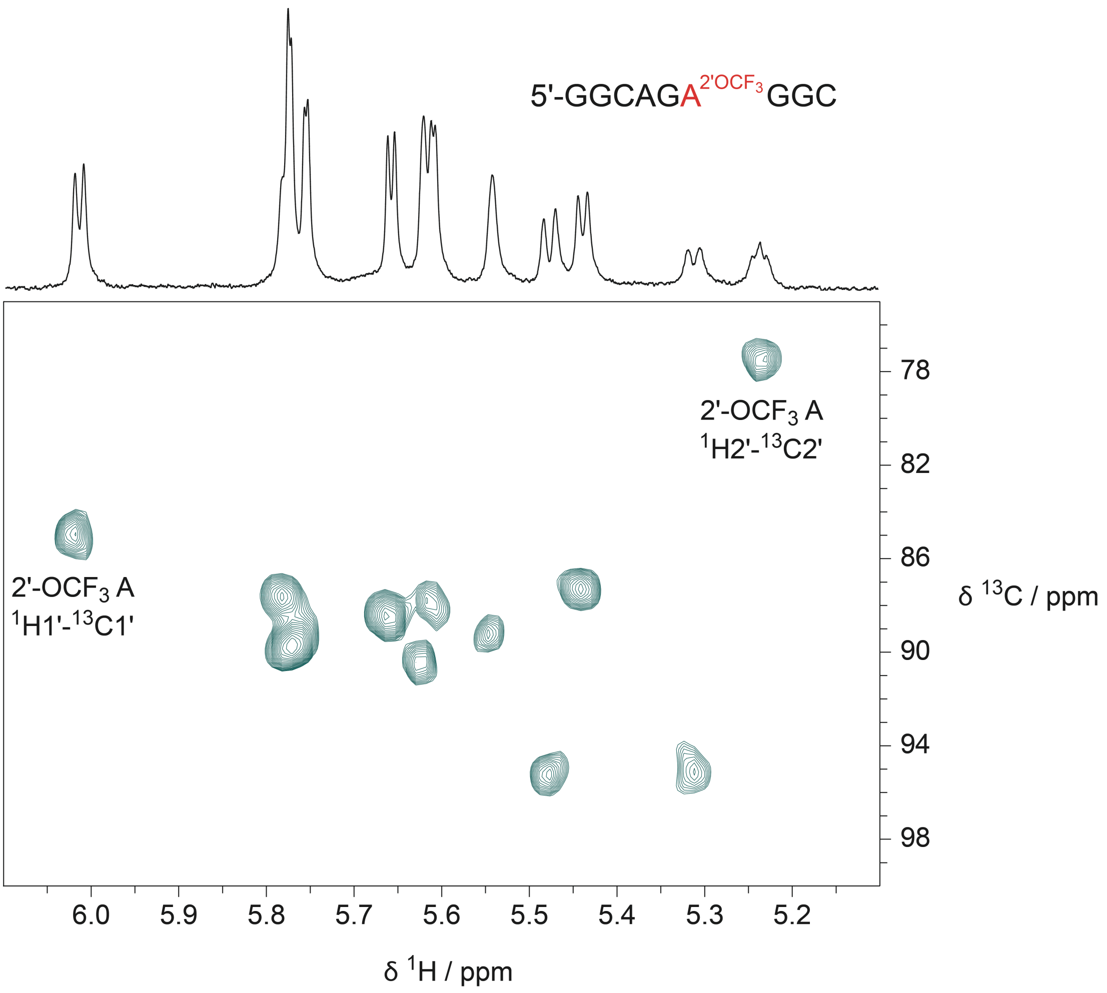

Supporting Figure 2. ^{1}H-^{13}C HSQC spectrum of 2'-OCF_3 modified single-stranded RNA (600 MHz Bruker). c_{RNA} = 0.3 mM; 15 mM sodium cacodylate, 25 mM NaCl, 3 mM NaN_3, 100 % D_2O, pH 6.5., 298 K.

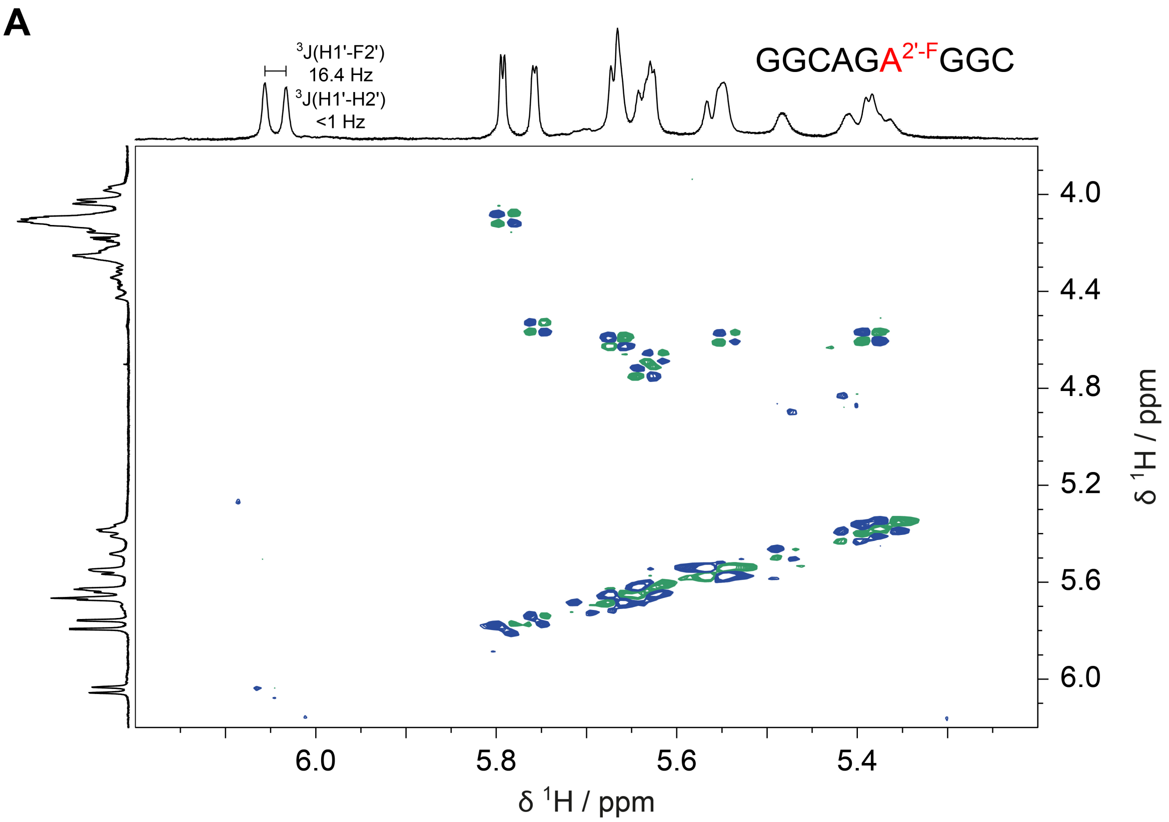

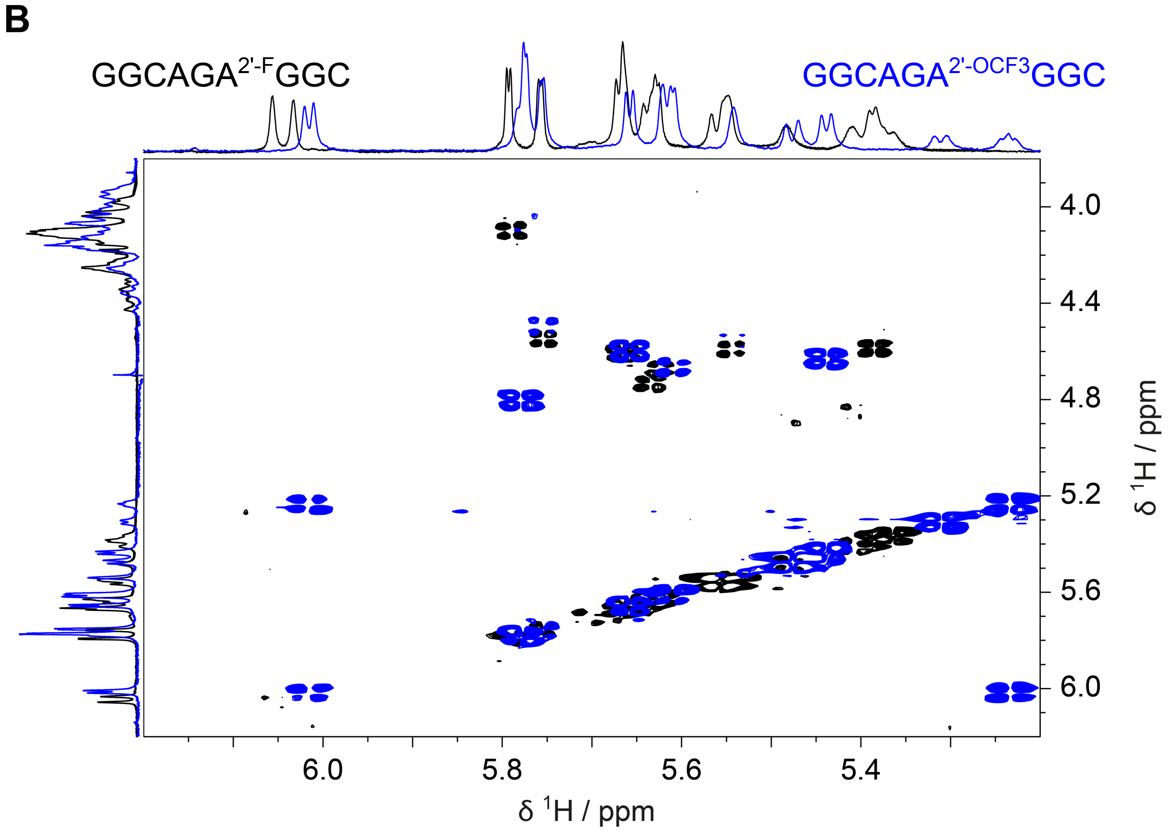

Supporting Figure 3. $^1H/^1H$ ECOSY NMR spectrum of single-stranded RNA 5‘-GGCAG(2’-F-A)GGC (**A**) and overlay of 2’-F-A & 2’-OCF_3-A modified 9 nt RNA (**B**). c_{RNA} = 0.3 mM, in 15 mM sodium cacodylate, 25 mM NaCl, 3 mM NaN_3, 100 % D_2O, pH = 6.5, 298 K.

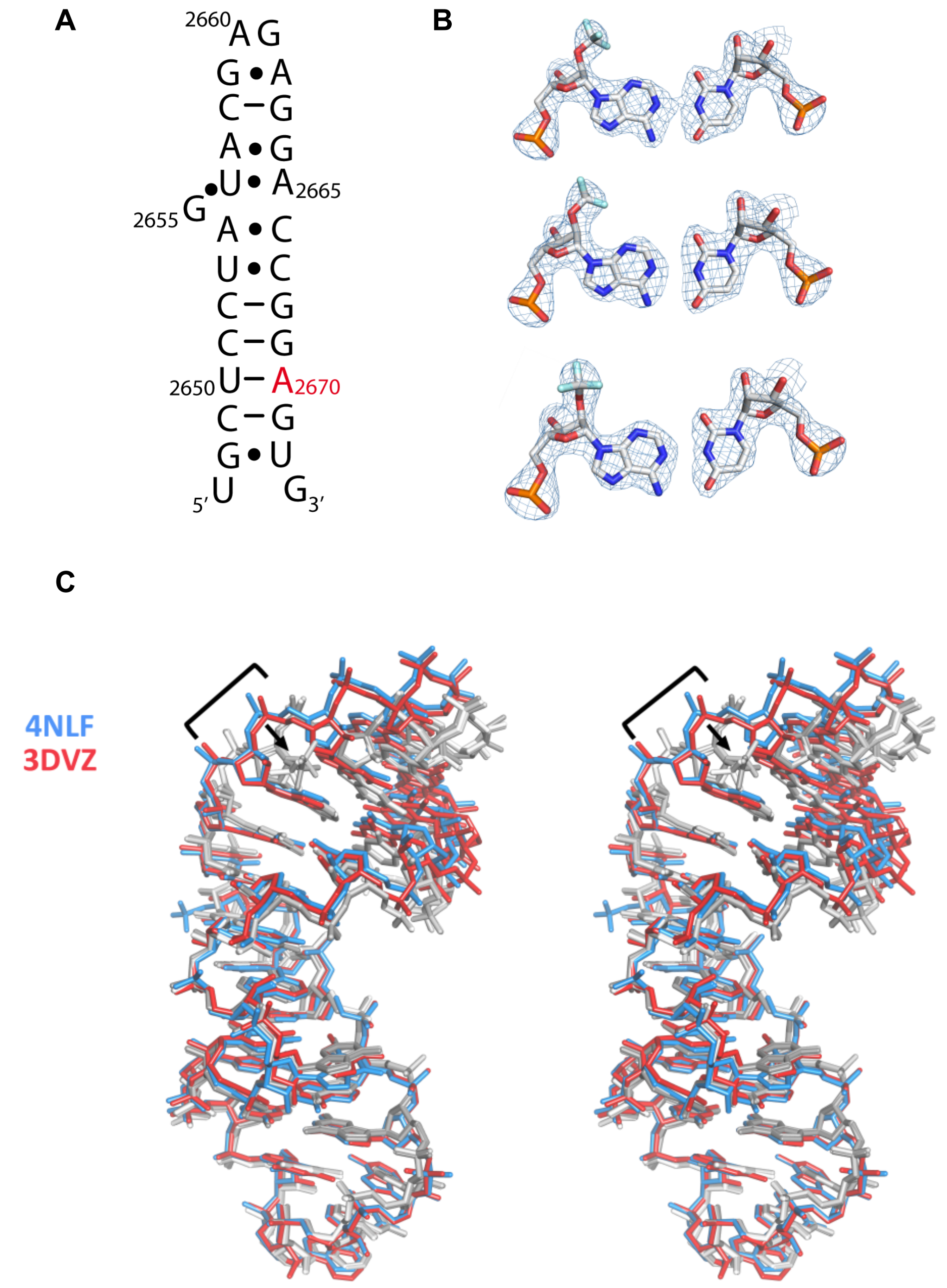

Supporting Figure 4. X-ray structure of the A2670-2′-OCF_3 modified RNA at 2.4 Å resolution (monoclinic form). (**A**) Secondary structure of the *E. coli* Sarcin-ricin stem-loop (SRL) RNA used for crystallization. (**B**) $2F_{obs} - F_{calc}$ electron density map contoured at 1.5 σ level showing the three U2650/2′-OCF_3-A2670 base pairs from the asymmetric unit. Note the slightly different orientation of the 2′-OCF_3 between base-pairs in the bottom vs middle and top. (**C**) Crossed-eyes stereo view showing a superposition of unmodified (red, PDB ID 3DVZ) and all three 2′-OCF_3-A2670 RNA from the asymetric unit (grey, this work). The 2′-SCF_3-modified RNA is also shown for comparison (blue, PDB 4NLF), with the modification visible in the middle-left part of the duplex. Arrow indicates position of the 2′-OCF_3 modification in the current structure. The kink induced by the modification in this crystal form is highlighted by on opened bracket.

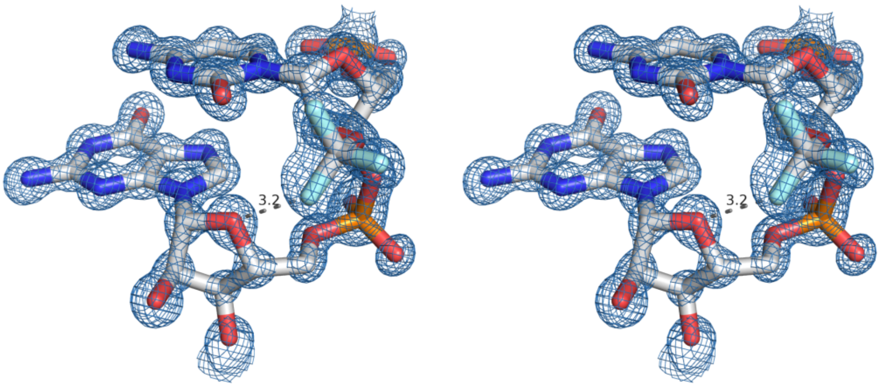

Supporting Figure 5. Detailed cross-eyes stereo view of the C2667-OCF_3 modified RNA tetragonal form showing the G2667-G2668 step and the close approach of fluorine and oxygen atoms (numbers are distances in Å). The $2F_{obs} - F_{calc}$ electron density map contoured at 1.5 σ level is shown.

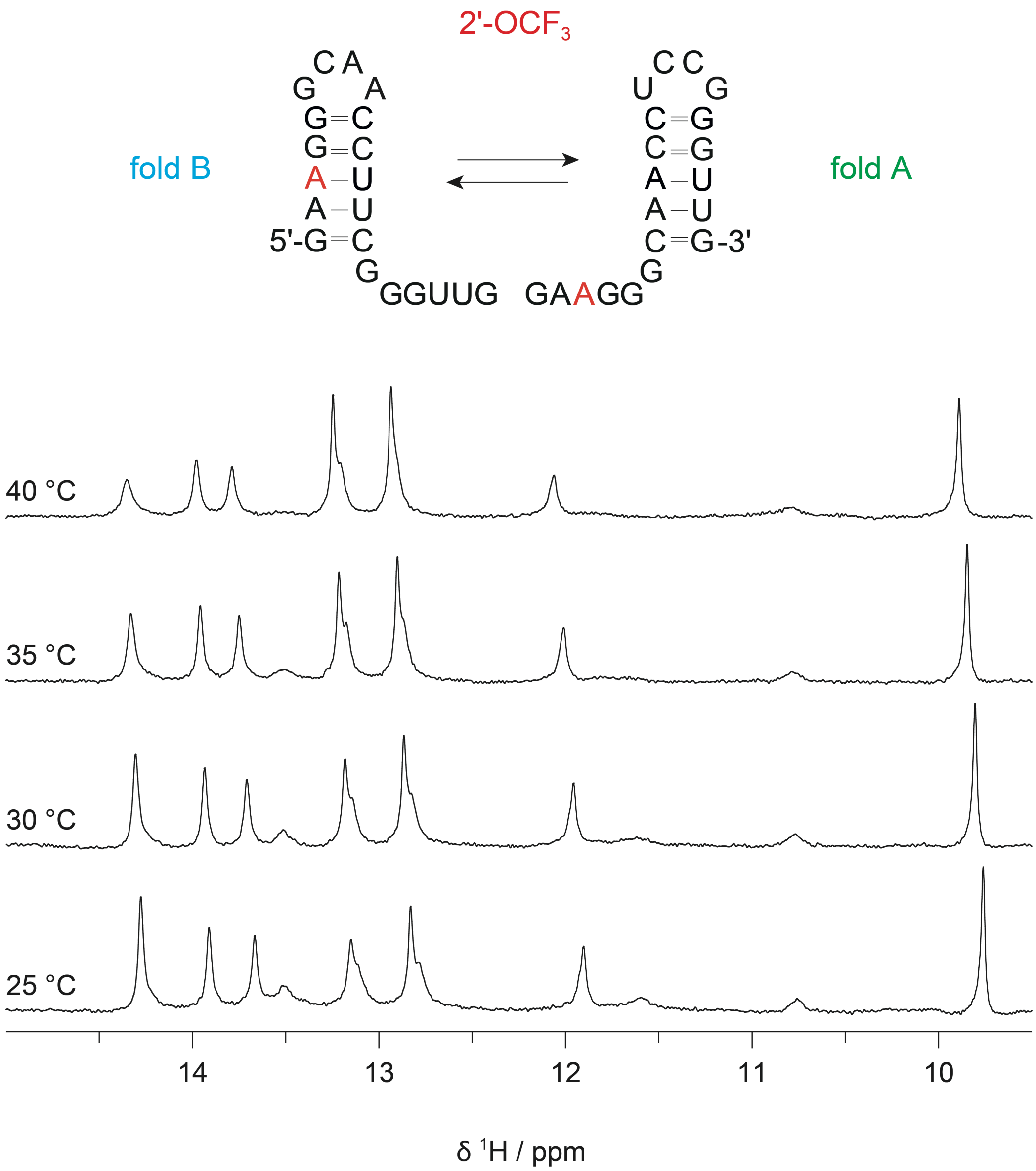

Supporting Figure 6. ^{1}H-NMR imino spectra of 2'-OCF_3 modified bistable RNA (600 MHz Bruker) at varying temperatures. c_{RNA} = 0.5 mM; 15 mM sodium cacodylate, 25 mM NaCl, 3 mM NaN_3, H_2O/D_2O = 9/1, pH 6.5. Nucleotides in red color indicate the positions for 2′-OCF_3 modification.

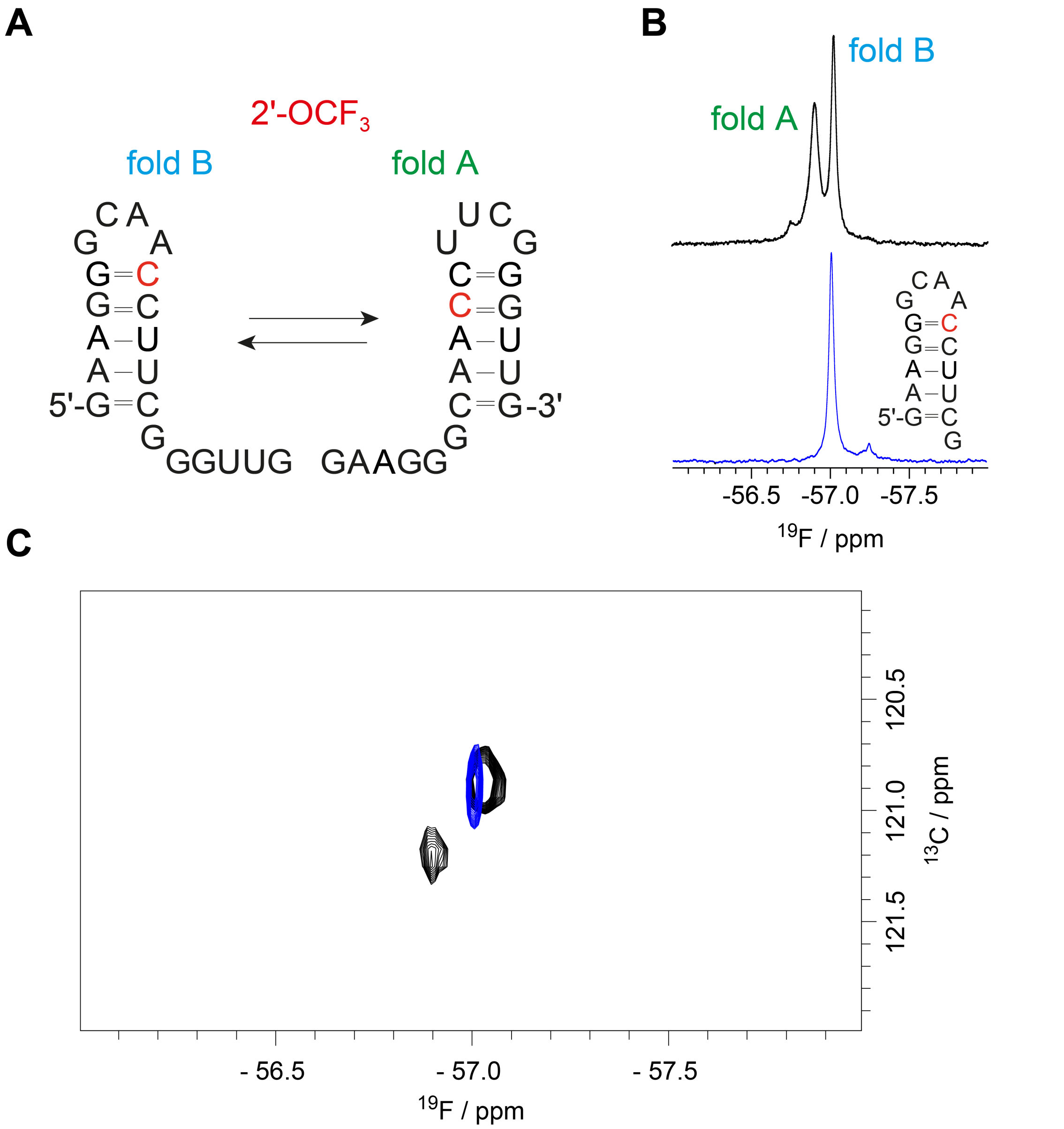

Supporting Figure 7. ^{19}F-NMR 1D and 2D spectra of 2'-OCF_3 modified bistable RNA (600 MHz Bruker). (**A**) 2'-OCF_3 modified bistable RNA equilibrium; (**B**) ^{19}F-NMR spectra of bistable RNA (top) compared to reference hairpin (bottom), (**C**) ^{13}C-^{19}F HSQC NMR spectrum of bistable RNA (black) and reference hairpin (blue). c_{RNA} = 0.3 mM; 15 mM sodium cacodylate, 25 mM NaCl, 3 mM NaN_3, H_2O/D_2O = 9/1, pH 6.5, 298 K. Nucleotides in red color indicate the positions for 2′-OCF_3 modification.

References

[1] Spitzer, R.; Kloiber, K.; Tollinger, M.; Kreutz, C. Kinetics of DNA refolding from longitudinal exchange NMR spectroscopy. *Chembiochem* **2011**, *12*, 2007–2010.

[2] Olieric, V.; Rieder, U.; Lang, K.; Serganov, A.; Schulze-Briese, C.; Micura, R.; Dumas, P.; Ennifar, E. A fast selenium derivatization strategy for crystallization and phasing of RNA structures. *RNA* **2009**, *15*, 707-715.

[3] Kabsch, W. Integration, scaling, space-group assignment and post-refinement. *Acta Cryst*. **2010**, *D66*, 133–144.

[4] Vagin, A., Teplyakov, A. Molecular replacement with MOLREP, *Acta Cryst*. **2010**, *D66*, 22-25.

[5] Afonine, P. V.; Grosse-Kunstleve, R. W.; Echols, N.; Headd, J. J.; Moriarty, N. W.; Mustyakimov, M.; Terwilliger, T. C.; Urzhumtsev, A.; Zwart, P. H; Adams, P. D. Towards automated crystallographic structure refinement with *phenix.refine*, *Acta Cryst*. **2012**, *D68*, 352-367.

[6] Emsley, P.; Lohkamp, B.; Scott, W. G.; Cowtan, K. Features and development of Coot. *Acta Cryst*. **2010**, *D66*, 486-501.
